# Comparative analysis of the human hepatic and adipose tissue transcriptomes during LPS-induced inflammation leads to the identification of differential biological pathways and candidate biomarkers

**DOI:** 10.1186/1755-8794-4-71

**Published:** 2011-10-06

**Authors:** Ewa Szalowska, Martijn Dijkstra, Marieke GL Elferink, Desiree Weening, Marcel de Vries, Marcel Bruinenberg, Annemieke Hoek, Han Roelofsen, Geny MM Groothuis, Roel J Vonk

**Affiliations:** 1 Centre for Medical Biomics, University Medical Centre Groningen (UMCG), University of Groningen, Antonius Deusinglaan 1, 9713 AV Groningen, The Netherlands; 2 Division of Pharmacokinetics, Toxicology and Targeting; Department of Pharmacy University of Groningen, Antonius Deusinglaan 1, 9713 AV Groningen, The Netherlands; 3 Department of Genetics, University Medical Centre Groningen (UMCG), P.O. Box 30001, 9700 RB Groningen, The Netherlands; 4 Department of Obstetrics and Gynecology, University Medical Centre Groningen (UMCG), PO Box 30.001, 9700 RB Groningen, The Netherlands; 5 Wageningen University and Research Centre/RIKILT, Cluster of Toxicology and Effect Analysis, Postbus 230, 6700AE Wageningen, The Netherlands

## Abstract

**Background:**

Insulin resistance (IR) is accompanied by chronic low grade systemic inflammation, obesity, and deregulation of total body energy homeostasis. We induced inflammation in adipose and liver tissues *in vitro *in order to mimic inflammation *in vivo *with the aim to identify tissue-specific processes implicated in IR and to find biomarkers indicative for tissue-specific IR.

**Methods:**

Human adipose and liver tissues were cultured in the absence or presence of LPS and DNA Microarray Technology was applied for their transcriptome analysis. Gene Ontology (GO), gene functional analysis, and prediction of genes encoding for secretome were performed using publicly available bioinformatics tools (DAVID, STRING, SecretomeP). The transcriptome data were validated by proteomics analysis of the inflamed adipose tissue secretome.

**Results:**

LPS treatment significantly affected 667 and 483 genes in adipose and liver tissues respectively. The GO analysis revealed that during inflammation adipose tissue, compared to liver tissue, had more significantly upregulated genes, GO terms, and functional clusters related to inflammation and angiogenesis. The secretome prediction led to identification of 399 and 236 genes in adipose and liver tissue respectively. The secretomes of both tissues shared 66 genes and the remaining genes were the differential candidate biomarkers indicative for inflamed adipose or liver tissue. The transcriptome data of the inflamed adipose tissue secretome showed excellent correlation with the proteomics data.

**Conclusions:**

The higher number of altered proinflammatory genes, GO processes, and genes encoding for secretome during inflammation in adipose tissue compared to liver tissue, suggests that adipose tissue is the major organ contributing to the development of systemic inflammation observed in IR. The identified tissue-specific functional clusters and biomarkers might be used in a strategy for the development of tissue-targeted treatment of insulin resistance in patients.

## Background

Adipose tissue is an important metabolic and endocrine organ that secretes numerous biologically active proteins (adipokines) such as leptin, adiponectin, many cytokines, and chemokines [1]. During the development of obesity, adipose tissue undergoes a switch from being mainly a metabolic organ towards an organ that shows substantial pro-inflammatory activity, associated with decreased insulin sensitivity, declined expression of adiponectin and enhanced production of pro-inflammatory cytokines and chemokines. These processes are believed to lead to low-grade inflammation and eventually systemic insulin resistance (IR) and type 2 diabetes (T2D) [2]. However, it is not yet understood how the change in the inflamed adipose tissue transcriptome and secretome leads to the development of IR. In addition to adipose tissue, the liver as an important metabolic and endocrine organ secreting many hormones, chemokines and cytokines, is also affected in obesity [3,4]. In a fatty liver, inflammation with activated NF-κB signaling and upregulated cytokines (IL-6, TNFα, and IL-1β) seems to be a pivotal event leading to the development of liver insulin resistance and non-alcoholic fatty liver disease (NAFLD) which both strongly predispose to the development of systemic IR and T2D. Except for the few proteins known to be produced and secreted by the liver during inflammation little is known about other protein factors which alone or by interacting with the secretome of inflamed adipose tissue could contribute to the development of systemic inflammation and insulin resistance in humans [5-8].

Lipopolysachcaride (LPS) is a compound of the cell wall of Gram-negative bacteria which induces inflammatory reactions and upregulates many cytokines and chemokines via TLRs. Besides its role in inflammation it was shown several times that LPS triggers hyperglycemia and IR in rats and humans [9-12] and induces weight gain and liver IR in mice [13,14].

In our studies, we aimed to identify molecular processes affected during inflammation in human AT and LT in order to better understand their roles in the inflammation- related development of IR/T2D *in vivo*. Therefore, we challenged human adipose tissue (omentum) and liver tissue slices with LPS and analyzed gene expression changes by DNA microarray technology and performed Gene Ontology (GO), gene functional classification/clustering analysis by means of publicly available bioinformatics tools: Database for Annotation, Visualization, and Integrated Discovery (DAVID) and Search Tool for the Retrieval of Interacting Genes/Proteins (STRING).

Additionally, we aimed to compare the secretomes of adipose and liver tissues during inflammation in order to better understand how these two organs can contribute to the development of systemic inflammation and IR. The transcriptome data were used to predict genes encoding for secreted proteins, by means of SecretomeP. The comparative analysis of the predicted secretomes led to the identification of differential candidate biomarkers for the inflamed adipose tissue and the inflamed liver tissue. Significantly changed genes detected in the adipose tissue secretome, but not in the inflamed liver tissue secretome were considered as the top candidate biomarkers related to inflammation of adipose tissue and these transcriptome data were confirmed by proteomics analysis of the inflamed adipose tissue culture medium.

The identified biological processes and biomarkers indicative for the inflamed adipose tissue or the inflamed liver tissue might be used for tissue-specific diagnosis of insulin resistance related to inflammation and thereby facilitate more targeted treatment of insulin resistant patients.

## Methods

### Human liver tissue

Human liver tissue (n = 5) was obtained and prepared as described previously [15]. The donors of livers were healthy males aged 16-34 years, with BMI 23.1-27.7. The information about the medical history was not available. The research protocols conformed the Helsinki Declaration, were approved by the local Medical Ethical Committee of the UMCG, and patients gave written informed consent to participate in the study.

### Preparation and incubation of liver slices

Human liver slices were prepared and incubated as described previously [15]. Liver slices were incubated at 37°C in Williams Medium E in the presence or absence of 100 μg/ml LPS. 24 h after incubation, slices were frozen in liquid nitrogen and stored at -80°C.

### Human adipose tissue

Omentum AT biopsies used for the transcriptome analysis were obtained from 7 Caucasian women undergoing surgery because of benign gynecological problems. The women were in general good health, had no history or symptoms of T2D or inflammatory diseases. The subjects were aged between 30 and 45 years, with BMI ranging from 23 to 29. The omentum biopsies were taken at the lower edge of the omentum using scissors. The omentum AT biopsy used in proteomics experiment was obtained from a healthy woman aged 59 years, with a BMI of 35.5 The research protocols conformed the Helsinki Declaration, were approved by the local Medical Ethical Committee of the UMCG, and patients gave written informed consent to participate in the study.

### Preparation and incubation of adipose tissue biopsies

The human AT surgical biopsies were processed as described previously [16]. In our studies AT was cultured in the absence/presence of LPS (100 μg/ml) for 24 hours. After the culture time the fat tissue was snap-frozen in liquid nitrogen and stored in -80°C until further processing.

### RNA isolation

RNA was extracted from adipose tissue using RNeasy Lipid Tissue Mini Kit (Qiagen, Venlo, The Netherlands) according to the manufacturer's instructions. RNA extraction from human liver slices was performed as described previously [15]. The RNA concentration was determined by Nano Drop ND-1000 Spectrophotometer (Isogen Ijsselstein, The Netherlands). The quality of total RNA was evaluated by capillary electrophoresis using an Agilent 2100 Bioanalyzer (Agilent Technologies, Palo Alto, Calif.).

### Illumina Human WG8-v2 Microarray Analysis

The Illumina platform was used for the gene expression analysis in adipose tissue. Biotin- labeled cRNA was generated from high-quality total RNA with the Illumina TotalPrep RNA amplification kit (Ambion). Briefly, 50 ng of total RNA was reversely transcribed with an oligo(dT) primer containing a T7 promoter. The first- strand cDNA was used to make the second strand. The purified second-strand cDNA, along with biotin UTPs, was subsequently used to generate biotinylated, antisense RNA of each mRNA in an *in vitro *transcription reaction. The size distribution profile for the labeled cRNA samples was evaluated by Bioanalyzer. After RNA labeling, 1.5ug of purified, labeled cRNA from each sample was hybridized at 55°C overnight with a Human-8 v2 expression Illumina Beadchip targeting 22000 transcripts. The beadchip was washed the following day. A signal was developed during incubation with Streptavidin-Cy3, and each chip was scanned with an Illumina Bead Array Reader.

The preprocessing of Illumina data was performed using the BeadStudio package with default settings. The background was subtracted and quantile normalization performed. Probes with "absent" signals in all samples (lower than or near to background levels) were removed from further analysis. To identify the differentially expressed genes in LPS treated samples versus controls eBayes test was performed and Benjamini Hochberg test corrected false discovery rate (FDR) ≤ 0.05. Probes with fold change ≥ 2 were used for further analysis. The calculations were performed in R, a language for statistical computing and graphics http://www.R-project.org.

### Affymetrix Human Genome U133 Plus 2.0 Array Analysis

The Affymetrix platform (55000 transcripts) was used for the liver tissue gene expression analysis. Double-stranded cDNA was synthesized from 1.5 μg total RNA using the One-Cycle Target Labeling Kit (Affymetrix Santa Clara, CA), and used as a template for the preparation of biotin-labeled cRNA using the GeneChip IVT Labeling Kit (Affymetrix Santa Clara, CA). Biotin-labeled cRNA was fragmented at 1 μg/μl following the manufacturer's protocol. After fragmentation, cRNA (10 μg) was hybridized at 45°C for 16 hours to the Human Genome U133 Plus 2.0 array (Affymetrix, Santa Clara, CA). Following hybridization, the arrays were washed, stained with phycoerythrin-streptavidin conjugate (Molecular Probes, Eugene, OR), and the signals were amplified by staining the array with biotin-labeled anti-streptavidin antibody (Vector Laboratories, Burlingame, CA) followed by phycoerythrin-streptavidin. The arrays were laser scanned with a GeneChip Scanner 3000 7G (Affymetrix, Santa Clara, CA) according to the manufacturer's instructions. Data was saved as raw image file and quantified using GCOS (Affymetrix).

Probe set summarization was performed using the RMA algorithm. Subsequently, baseline subtraction was performed setting the baseline to the median of all samples. To identify the differentially expressed genes in LPS treated samples versus controls an eBayes test was performed and Benjamini Hochberg test corrected false discovery rate (FDR) ≤ 0.05. Probes with fold change ≥ 2 were used for further analysis. The calculations were performed in R, a language for statistical computing and graphics http://www.R-project.org.

### Gene Functional Classification Analysis

The significant transcriptomes of AT and LT were uploaded to Database for Annotation, Visualization, and Integrated Discovery (DAVID) Bioinformatics Resource where the Gene Functional Classification tool was applied to generate clusters of functionally related genes. Additionally, the Functional Annotation Clustering tool was used to generate clusters of overrepresented Gene Ontology (GO) terms [17,18]. The HG-U133 Plus 2 and HUMANREF-8 V2 0 R3 11223162A were used as a background for the GO analysis of liver tissue and adipose tissue respectively. The GO terms after correction for FDR at p ≤ 0.05 (Benjamini Hochberg) were selected for further analysis and interpretation.

### Gene networks and pathways identification

The significant transcriptomes of adipose and liver tissues were uploaded to Search Tool for the Retrieval of Interacting Genes/Proteins 8.2 (STRING) where networks based on known and predicted protein-protein interactions were built and clustered into functional categories [19].

### Secretome prediction

From the significant transcriptome data obtained for adipose and liver tissues, the secretome prediction was performed with in-house developed software, which retrieved the information about the predicted secretomes from SecretomeP [20]. Genes were considered to belong to the secretome when they encoded for proteins with a predicted signal peptide (present in proteins that are secreted via the classical endoplasmic reticulum/Golgi-dependent pathway) or when their Neuronal Network (NN) score exceeded the value of 0.5, which classifies them as secreted via the non-classical pathway. Genes encoding for proteins which did not have a signal peptide nor had the NN-score below 0.5 were considered as genes encoding for intracellular proteins and were discarded from the final secretome analysis.

### Adipose tissue culture for the quantitative proteomics analysis

Quantitative secretome analysis was performed by Isotope-Labeled Amino Acid Incorporation Rates (CILAIR) as described previously [21]. Briefly, 6 g of fat tissue was used from one patient and divided into six Petri dishes containing 10 ml of lysine-free M199 medium (reference number 22340 Lys-free, Invitrogen) to deplete lysine from other sources (blood in the tissue) and supplemented with 50 μg/ml gentamicin. The tissue was incubated for 24 h. After this period, fresh M199 containing 70 mg/liter 13C-labeled lysine (L-[13C6, 14N2]lysine (Invitrogen) was added to all dishes for the next 24 hours to allow incorporation of the label into newly synthesized proteins, in the absence (3 dishes) or presence (3 dishes) of LPS (100 μg/ml). CILAIR is based on the incorporation rate of 13C-labeled lysine in newly synthesized secreted proteins. If this rate is different between two conditions for a specific protein the change in expression of this protein can be calculated by comparing the heavy/light ratios for the two conditions. After the 24 h incubation, media were collected and stored at -80°C until further processing. The sample preparation and protein identification by liquid chromatography coupled to mass spectrometry was performed as described previously [21]. ProteinPilot 2.0 software (Applied Biosystems) was used to analyze the mass spectra using the UniprotKB/Swiss-Prot database (release 54, January 2008, 276, 256 entries). The settings used in the analysis were the same as described previously [21].

### CILAIR data analysis

The statistical analysis to detect differences in the secretome of LPS-treated vs. control adipose tissue cultures was performed with in-house generated software that was developed using the open source MOLGENIS toolbox [22]. A two-sided unpaired Student's t-test was applied, and multiple testing correction was performed to control the false discovery rate (FDR) at FDR < 0.05.

The applied criteria for the proteins predicted to be secreted were the same as described above for the transcriptome data.

## Results

### Functional gene annotation analysis

**Figure 1:**
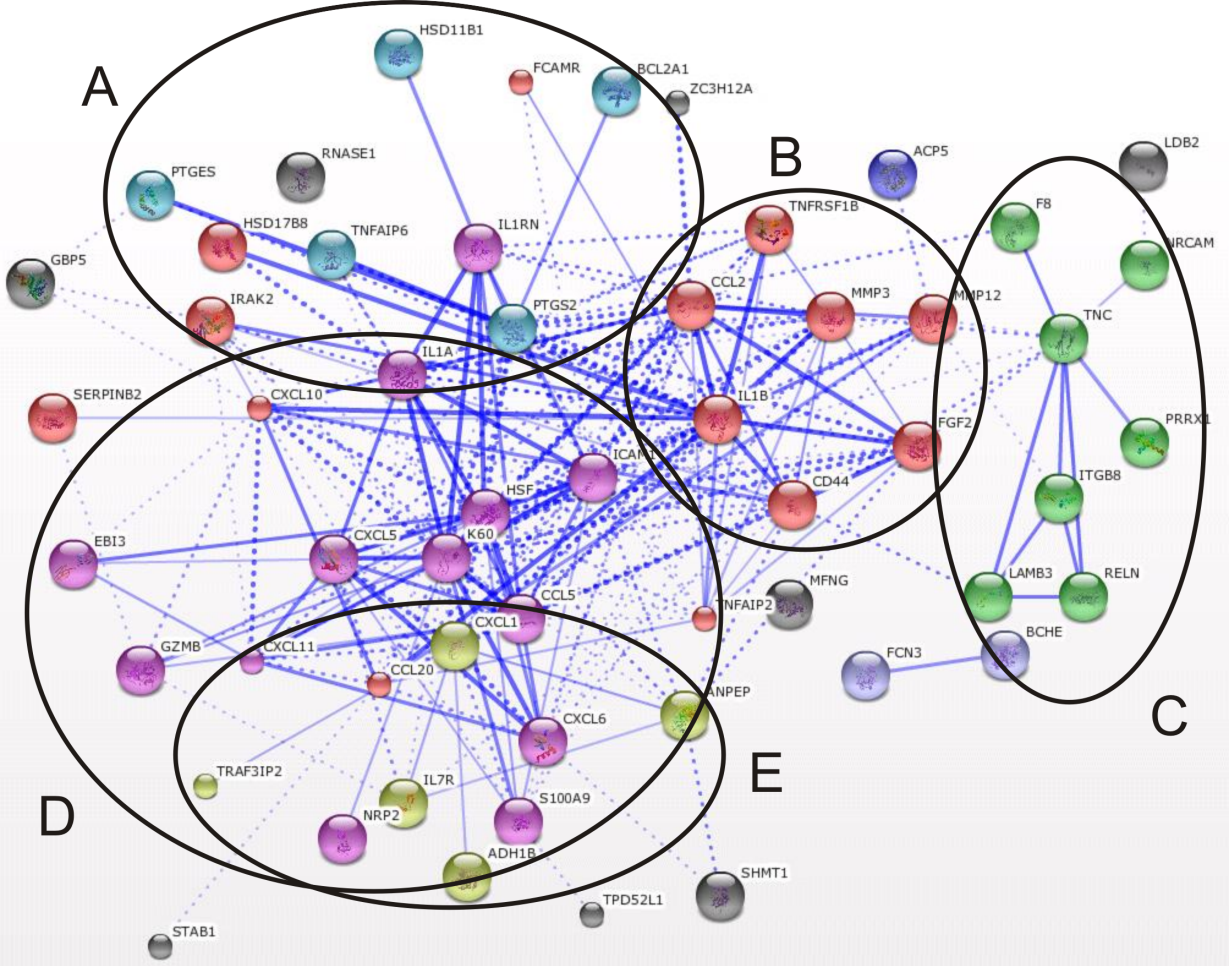
**The gene functional clusters identified for the significant, overlapping adipose and liver tissue transcriptomes**. The overlapping (shared) upregulated adipose tissue (n = 7) and liver tissue (n = 5) significantly changed transcriptome. The overlapping (shared) upregulated adipose tissue and liver tissue significantly changed transcriptome. Within the overlapping network we identified functional clusters related to: (A) (anti)apoptosis/inflammation; (B) mobilization of T-lymphocytes and monocytes; (C) matrix remodelling; (D) T and B cell activation and functioning; (E) interleukin 7 receptor activity.

**Figure 2:**
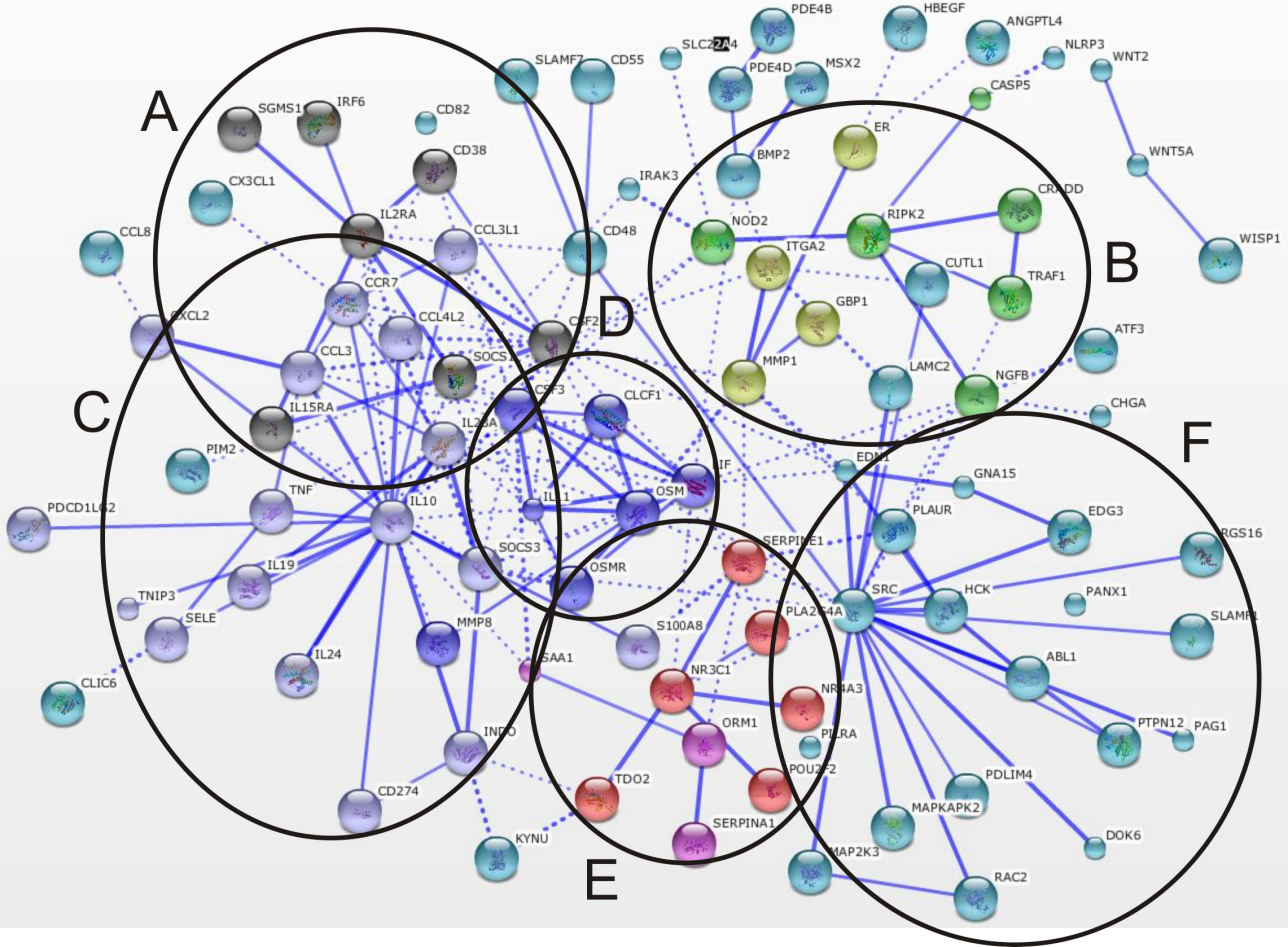
**The gene functional clusters identified for the significant, upregulated adipose tissue transcriptome**. The upregulated adipose tissue network (n = 7) contained 6 functional clusters:: (A) regulation of cytokine signaling; (B) cell adhesion & apoptosis; (C) IL-10 signaling; (D) growth and differentiation of hematopoietic cells; (E) glucocorticoid receptor signaling & acute phase response; (F) plasminogen activation system.

**Figure 3:**
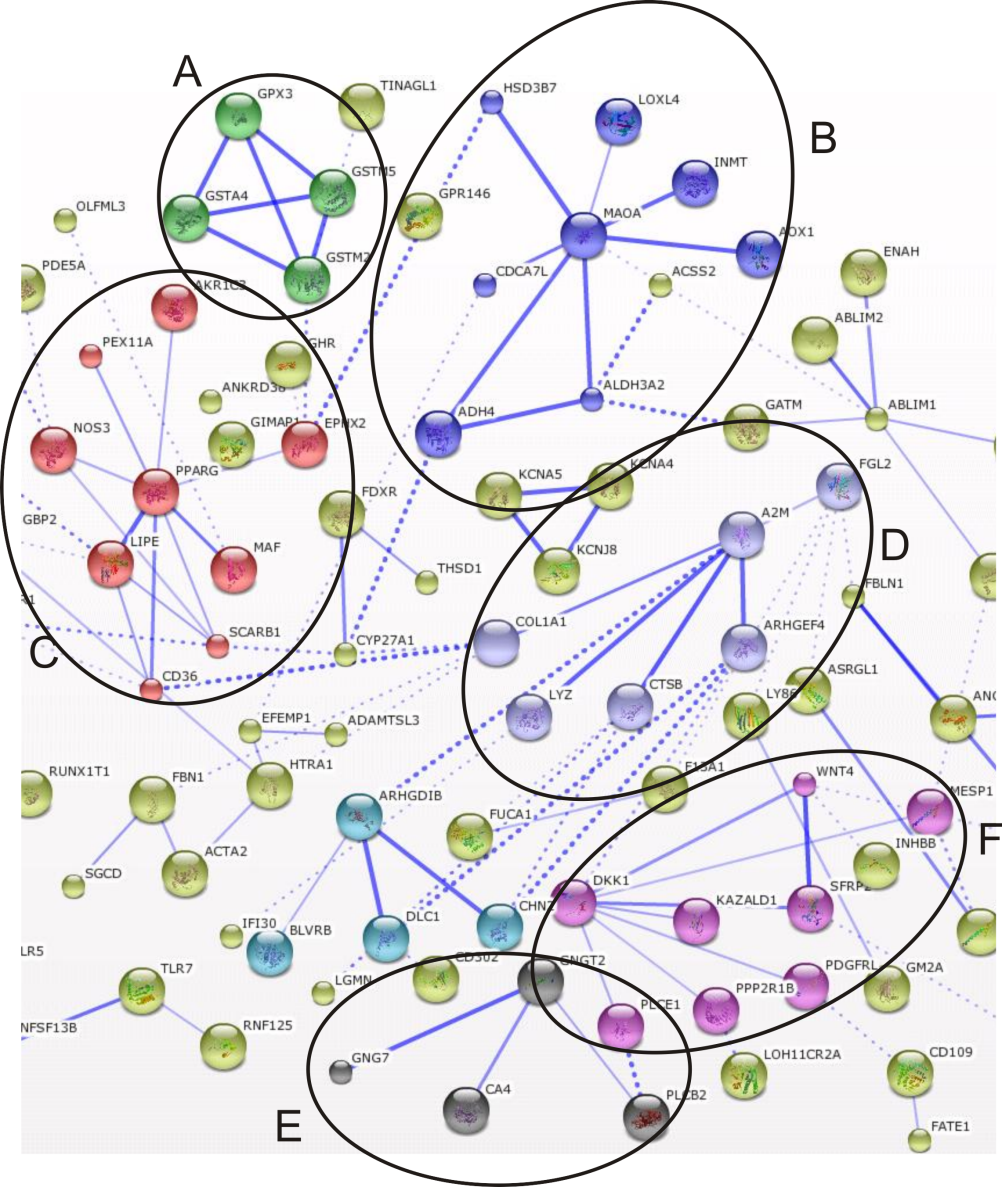
**The gene functional clusters identified for the significant, downregulated adipose tissue transcriptome**. The downregulated adipose tissue network(n = 7) had 6 functional clusters: (A) cellular defense against toxic compounds; (B) redox reactions; (C) PPARγ signaling; (D) innate immune system; (E) G-receptor signaling; (F) Wnt-signaling.

**Figure 4:**
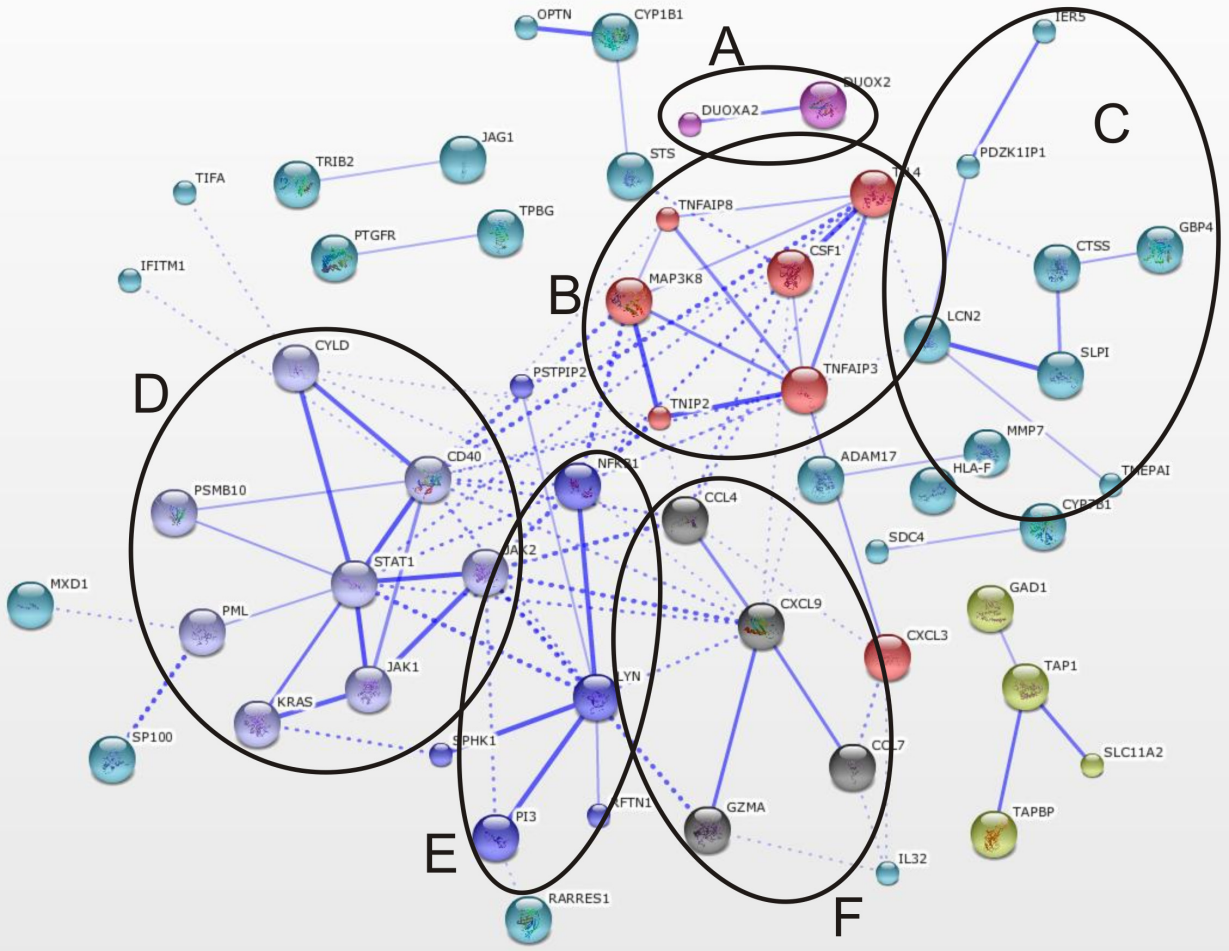
**The gene functional clusters identified for the significant, upregulated liver tissue transcriptome**. The liver tissue upregulated network (n = 5) consisted of 6 clusters: (A) ROS production; (B) innate immune system; (C) extracellular matrix remodelling; (D) JAK-STAT signaling; (E) NFκB signaling; (F) chemo-attraction of T- and NK-cells.

**Figure 5:**
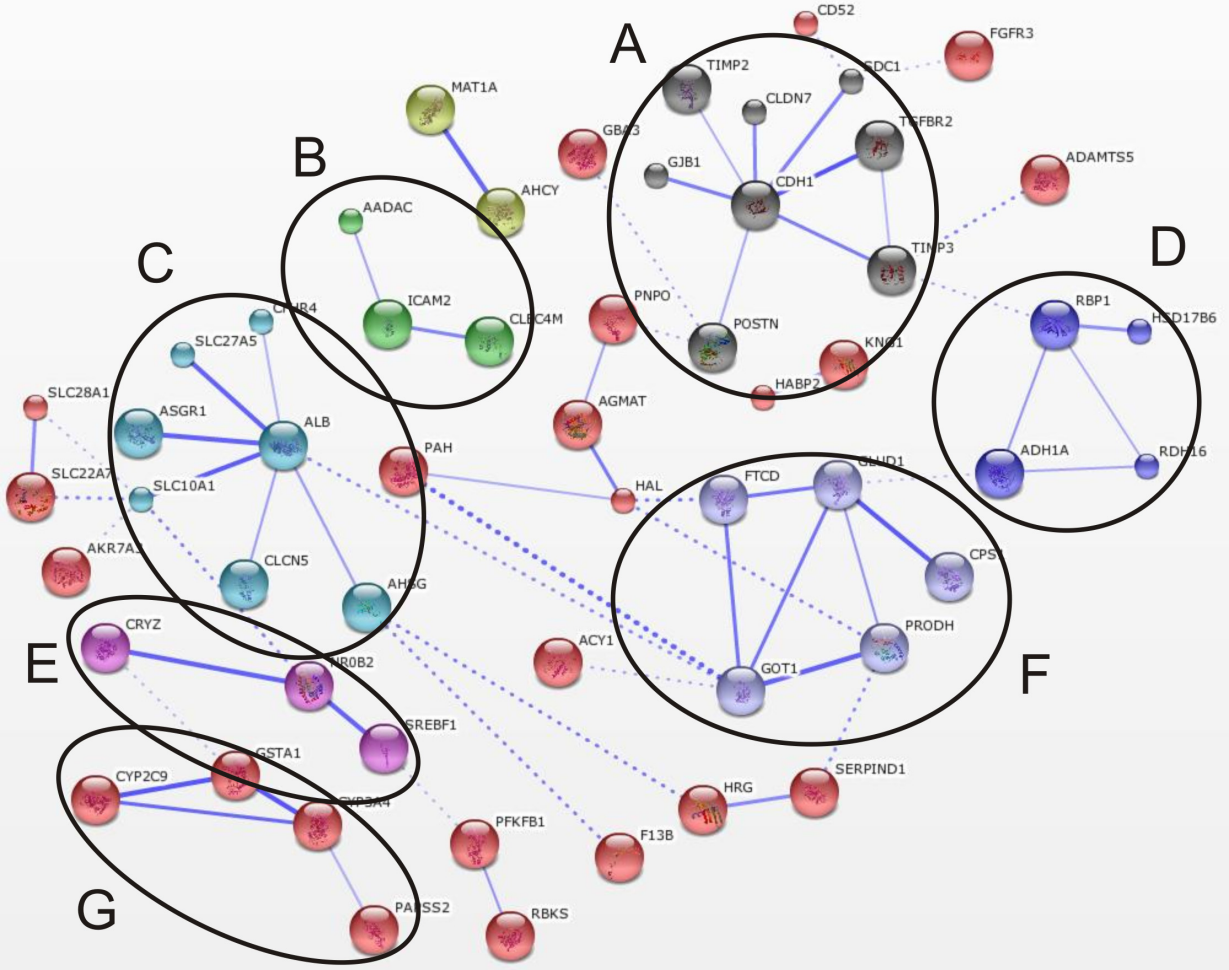
**The gene functional clusters identified for the significant, downregulated liver tissue transcriptome**. The downregulated liver tissue network (n = 5) contained 7 functional clusters: (A) cell-cell adhesion; (B) leukocytes functioning; (C) amino acids metabolism; (D) redox reactions; (E) sterol metabolism; (F) amino acids and nucleotide metabolism; (G) cytochrome P450.

The transcriptome data analysis revealed that in adipose tissue 667 genes were significantly affected (322 were upregulated and 345 were downregulated) after exposure to LPS. In liver tissue we detected 483 significantly changed genes (283 were upregulated and 200 were downregulated). The overlapping significant transcriptome shared by both tissues consisted of 82 transcripts. The significantly changed genes found in adipose tissue and liver tissue which were not present on both platforms were discarded from further analysis (47 and 42 respectively). Functional gene annotation analysis of significantly upregulated genes in adipose tissue (including the overlapping genes with the liver tissue significant transcriptome) led to the identification of functional groups such as: chemokines; growth and differentiation of hematopoietic precursors; (anti)apoptosis; modulation of immune response; T-, B-, leukocytes, and NK-cells activation, suppression of cytokine signaling (SOCS), extracellular matrix remodeling, and upregulation of numerous transporters, (Additional file 1, Table S1). Within the downregulated gene functional groups we identified: lysosomal/endosomal system activity, basement membrane components, extracellular matrix components, cell adhesion and migration, deoxy-ribonucleases activity, and detoxification, (Additional file 1, Table S2). A similar analysis was performed for liver tissue and within the upregulated gene functional groups we identified: chemokines; matrix remodeling; (anti)apoptosis; cell adhesion and migration; T- and NK- cell activity; and breakdown of extracellular matrix/tissue remodeling, (Additional file 1, Table S3). The functional classification of the downregulated genes led to identification of groups such as: amino acid metabolism, membrane activity, redox/detoxification reactions, cell adhesion and mitochondrial functions, (Additional file 1, Table S4). Additionally, in order to better visualize the similarities and differences between the adipose tissue and liver tissue transcriptomes during inflammation we performed gene functional network reconstruction in STRING. The identified gene functional clusters such as: chemokine signaling, matrix remodelling, SOCS signaling, PPARγ and others are depicted in Figures 1, 2, 3, 4, and 5: the gene functional clusters identified for the significant, overlapping adipose and liver tissue transcriptomes (Figure 1), the significant, upregulated adipose tissue transcriptome (Figure 2), for the significant, downregulated adipose tissue transcriptome (Figure 3), the significant, upregulated liver tissue transcriptome (Figure 4), and the significant, downregulated liver tissue transcriptome (Figure 5).

### Gene Ontology analysis

**Figure 6:**
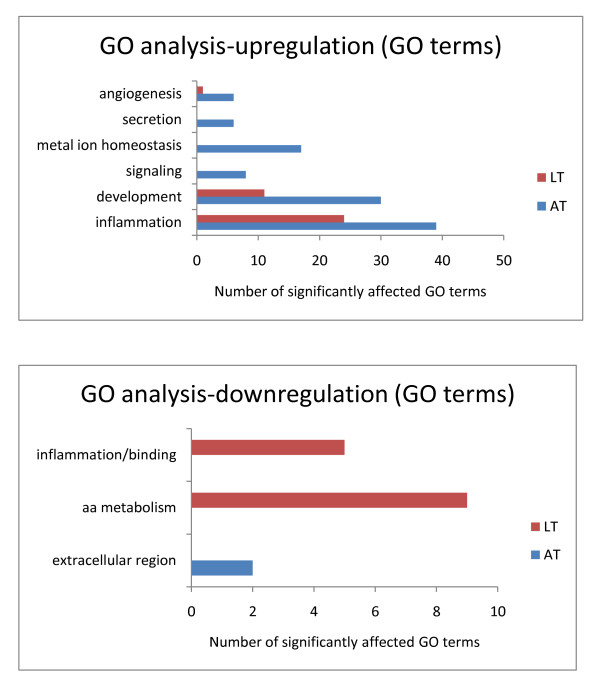
**GO analysis of the significant adipose and liver tissues transcriptomes**. The number of significantly enriched upregulated and downregulated GO terms in adipose tissue (AT), n = 7 and liver tissue (LT), n = 5 upon LPS treatment. The GO terms were categorized into broader GO categories such as: angiogenesis, secretion, metal ion homeostasis, signaling, development, inflammation, amino acid (aa) metabolism, and extracellular region.

Additionally, we performed GO ontology analysis. In adipose tissue we identified more upregulated GO terms compared to liver tissue (106 vs. 36) and for the down-regulated GO terms we detected 2 and 19 in adipose tissue and liver tissue respectively. The significantly upregulated GO terms were divided into broad categories such as "inflammation", "development", "signaling", "metal ion homeostasis", 'secretion' and "angiogenesis" and within the downregulated GO categories we distinguished: "extracellular region", "amino acid metabolism", and "polysaccharide binding". The GO terms identities within the GO categories are presented in the Additional file 2, Table S1, Additional file 2, Table S2, Additional file 2, Table S3, and Additional file 2, Table S4. Adipose tissue had more upregulated GO terms belonging to "inflammation", "development" and "angiogenesis" compared to liver tissue and had additional terms such as: "signaling", "metal ion homeostasis" and "secretion", (Figure 6; GO analysis of the significant adipose and liver tissues transcriptomes). Within the downregulated GO categories in adipose tissue we detected "extracellular region while in liver tissue- "amino acid metabolism" and "polysaccharide binding", (Figure 6; GO analysis of the significant adipose and liver tissues transcriptomes).

**Figure 7:**
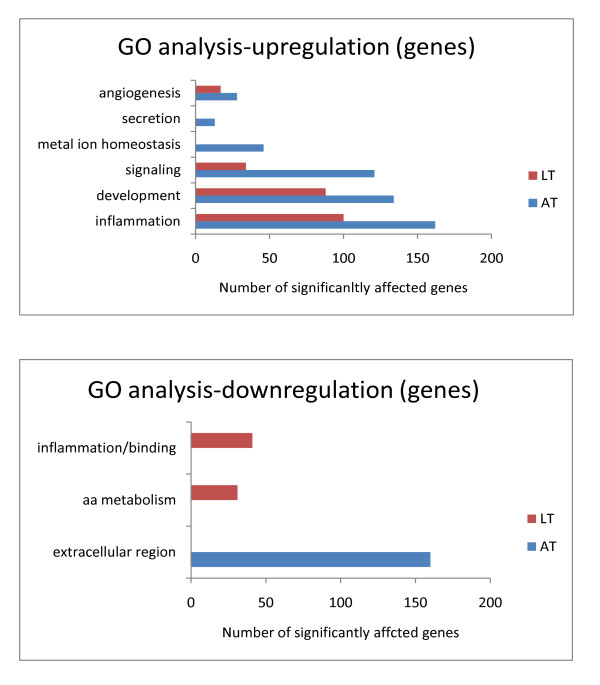
**Gene count analysis for the identified GO categories**. Number of genes significantly upregulated and downregulated in adipose tissue (AT), n = 7, and liver tissue (LT), n = 5 within GO categories (angiogenesis, secretion, metal ion homeostasis, signaling, development, inflammation, amino acid (aa) metabolism, and extracellular region).

When analyzing individual genes within the GO categories, a similar picture emerged -in general a larger number of genes belonging to the identified GO categories was altered in adipose tissue compared to liver tissue (Figure 7 Gene count analysis for the identified GO categories).

The names and Entrez IDs of genes up- and down- regulated in both tissues for each GO category are given in Additional file 3, Table S1, Additional file 3, Table S2.

### The differentially expressed genes and secretome prediction

Subsequent analysis of the significant transcriptome data was performed in order to select genes predicted to encode for secreted proteins (the predicted secretome). The analysis revealed that adipose tissue and liver tissue share 66 genes predicted to encode for secreted proteins (46 were upregulated and 20 were downregulated). In the adipose tissue predicted secretome we identified additional 333 significantly changed genes encoding for secreted proteins (138 transcripts were upregulated and 195 -were downregulated) and within the liver tissue predicted secretome we identified 170 different genes encoding for secreted proteins (80 were upregulated and 90 were downregulated).

In our studies we were mostly interested in the upregulated genes as they could be the best candidate biomarkers measurable in human serum. The information about gene expression of the highest upregulated genes in adipose and liver tissues is summarized in Table 1. The presented genes were subdivided in three categories: the first category contained genes which were significantly upregulated in both tissues (p ≤ 0.05, FC ≥ 2) as the best candidate biomarkers for the inflamed adipose and liver tissues. The second category contained genes significantly upregulated in adipose tissue (p ≤ 0.05, FC ≥ 2), but not changed in liver tissue, as the best candidate biomarkers for the inflamed adipose tissue. The third category contained genes significantly upregulated in liver tissue (p ≤ 0.05, FC ≥ 2) and unchanged in adipose tissue (p > 0.05) as the best source of candidate biomarkers for the inflamed liver tissue. The entire list of genes encoding for the predicted inflammatory secretomes of adipose and liver tissues is given in Additional file 4, Table S1, Additional file 4, Table S2, Additional file 4, Table S3.

**Table 1:** The most differential predicted secretome of adipose and liver tissues

**ACC**.	NAME4	GENE	AT FC	AT p value	AT avg - LPS	AT avg + LPS	AT std - LPS	AT std + LPS	LT FC	LT p value	LT avg - LPS	LT avg + LPS	LT std - LPS	LT std + LPS
P01584	INTERLEUKIN 1, BETA	**IL1B**	**20**	**1, 18E-06**	**584, 3**	**11733, 8**	**496, 8**	**3295, 7**	**100**	**0, 0002**	**9, 5**	**957, 6**	**17, 6**	**305, 3**
P10147	CHEMOKINE (C-C MOTIF) LIGAND 3	**CCL3**	**19.8**	**2, 0E-07**	**239, 3**	**4732, 6**	**160, 7**	**2239, 1**	**10.7**	**0, 0088**	**48, 7**	**521, 6**	**55, 4**	**226, 4**
Q96DR8	SMALL BREAST EPITHELIAL MUCIN	**MUCL1**	**17.4**	**5, 5E-05**	**36, 4**	**607, 9**	**20, 9**	**759, 2**	**5.8**	**0, 0008**	**5, 1**	**30, 0**	**2, 3**	**14, 8**
P18510	INTERLEUKIN 1 RECEPTOR ANTAGONIST	**IL1RN**	**12.5**	**9, 4E-06**	**16, 6**	**207, 4**	**6, 3**	**164, 7**	**6.5**	**0, 0148**	**118, 4**	**768, 3**	**127, 8**	**1132, 3**
P78556	CHEMOKINE (C-C MOTIF) LIGAND 20	**CCL20**	**11.4**	**7, 2E-06**	**1602, 6**	**18245, 7**	**1844, 3**	**2070, 8**	**7.5**	**0, 0199**	**1149, 2**	**8631, 9**	**306, 4**	**1263, 2**
P16619	CHEMOKINE (C-C MOTIF) LIGAND 3-LIKE 1	**CCL3L1**	**10.7**	**3, 4E-07**	**49, 7**	**1715, 4**	**38, 6**	**894, 1**	**34.5**	**0, 0088**	**48, 7**	**521, 6**	**55, 4**	**226, 4**
P42830	CHEMOKINE (C-X-C MOTIF) LIGAND 5	**CXCL5**	**7.8**	**1, 5E-04**	**735, 9**	**5489, 8**	**1215, 8**	**2385, 3**	**30.3**	**0, 0000**	**13, 9**	**330, 5**	**1, 9**	**23, 3**
P35354	PROSTAGLANDIN-ENDOPEROXIDE SYNTHASE 2	**PTGS2**	**6.8**	**5, 1E-05**	**1530, 5**	**10373, 9**	**1772, 5**	**2532, 4**	**7.9**	**0, 0478**	**39, 9**	**315, 1**	**3, 9**	**63, 5**
P13501	CHEMOKINE (C-C MOTIF) LIGAND 5/RANTES	**CCL5**	**6.3**	**1, 4E-06**	**252, 5**	**1588, 3**	**190, 4**	**458, 9**	**68**	**0, 0002**	**15, 8**	**1074, 0**	**17, 0**	**1425, 3**
P05120	SERPIN PEPTIDASE INHIBITOR	**SERPINB2**	**6**	**1, 1E-06**	**1825, 8**	**10909, 0**	**856, 7**	**2503, 1**	**8**	**0, 0022**	**25, 0**	**200, 7**	**37, 0**	**104, 9**
P01583	INTERLEUKIN 1, ALPHA	**IL1A**	**5.8**	**4, 4E-04**	**343, 2**	**1979, 4**	**211, 1**	**820, 6**	**4.3**	**0, 0043**	**7, 3**	**32, 1**	**2, 3**	**4, 2**
O14625	CHEMOKINE (C-X-C MOTIF) LIGAND 11	**CXCL11**	**5.4**	**1, 3E-03**	**19, 1**	**102, 5**	**15, 9**	**63, 2**	**20.4**	**0, 0269**	**10, 5**	**214, 6**	**17, 2**	**1379, 4**
P05231	INTERLEUKIN 6 (INTERFERON, BETA 2)	**IL6**	**4.5**	**2, 1E-05**	**4702, 1**	**21313, 8**	**3820, 2**	**1972, 7**	**17.8**	**0, 0097**	**40, 6**	**722, 6**	**52, 8**	**703, 0**
P08254	MATRIX METALLOPEPTIDASE 3	**MMP3**	**4**	**5, 5E-04**	**1333, 4**	**5331, 4**	**1458, 8**	**1982, 5**	**20.6**	**0, 0377**	**12, 2**	**251, 5**	**115, 5**	**725, 9**
P09038	FIBROBLAST GROWTH FACTOR 2 (BASIC)	**FGF2**	**3.7**	**7, 1E-04**	**173, 4**	**642, 5**	**165, 2**	**200, 0**	**3.5**	**0, 0020**	**13, 7**	**48, 4**	**5, 5**	**32, 0**
P39900	MATRIX METALLOPEPTIDASE 12 (MACROPHAGE ELASTASE)	**MMP12**	**3.7**	**1, 5E-04**	**22, 1**	**81, 7**	**8, 9**	**41, 1**	**5.8**	**0, 0220**	**19, 0**	**110, 8**	**31, 8**	**102, 9**
P09341	CHEMOKINE (C-X-C MOTIF) LIGAND 1	**CXCL1**	**3**	**1, 0E-03**	**4666, 1**	**14183, 2**	**5268, 4**	**3237, 7**	**23.5**	**0, 0013**	**70, 1**	**1648, 1**	**45, 5**	**615, 2**
P02778	CHEMOKINE (C-X-C MOTIF) LIGAND 10	**CXCL10**	**2.9**	**1, 4E-03**	**361, 5**	**1045, 7**	**248, 5**	**461, 4**	**17**	**0, 0431**	**72, 3**	**1234, 9**	**4543, 9**	**297, 8**
P80162	CHEMOKINE (C-X-C MOTIF) LIGAND 6	**CXCL6**	**2.8**	**1, 3E-03**	**2632, 1**	**7420, 4**	**2720, 0**	**1531, 1**	**22**	**0, 0033**	**20, 4**	**447, 3**	**49, 8**	**174, 3**
P10144	GRANZYME B	**GZMB**	**2.8**	**5, 1E-04**	**31, 8**	**87, 9**	**8, 5**	**38, 9**	**6.1**	**0, 0253**	**11, 9**	**73, 1**	**7, 6**	**91, 3**
O60462	NEUROPILIN 2	**NRP2**	**2.6**	**7, 0E-06**	**25, 0**	**65, 5**	**9, 0**	**7, 5**	**2.6**	**0, 0211**	**27, 9**	**72, 7**	**4, 8**	**15, 3**
P10145	INTERLEUKIN 8	**IL8**	**2.5**	**2, 1E-03**	**9738, 0**	**23999, 7**	**7532, 4**	**1532, 3**	**6**	**0, 0038**	**358, 9**	**4271, 3**	**480, 9**	**1244, 2**
P13500	CHEMOKINE (C-C MOTIF) LIGAND 2	**CCL2**	**2.3**	**5, 1E-06**	**5528, 4**	**12745, 4**	**1561, 6**	**1659, 7**	**3.7**	**0, 0435**	**726, 0**	**2675, 4**	**321, 4**	**366, 3**
P16581	SELECTIN E (ENDOTHELIAL ADHESION MOLECULE 1)	**SELE**	**105.1**	**1, 9E-06**	**5, 3**	**558, 7**	**4, 9**	**191, 7**	*4, 6*	0, 6116	6, 9	31, 4	4, 7	31, 9
P04141	COLONY STIMULATING FACTOR 2	**CSF2**	**82.5**	**1, 7E-07**	**9, 0**	**742, 5**	**12, 3**	**402, 4**	*1, 6*	0, 4180	1, 6	2, 7	5, 2	2, 5
Q9BYE3	LATE CORNIFIED ENVELOPE 3D	**LCE3D**	**46**	**1, 2E-05**	**-2, 7**	**70, 3**	**4, 1**	**103, 9**	*-1, 2*	0, 7820	8, 2	7, 0	5, 1	6, 7
P02763	OROSOMUCOID 1	**ORM1**	**26.6**	**2, 3E-03**	**12, 4**	**329, 5**	**8, 8**	**470, 6**	*1, 0*	0, 7173	4506, 7	4609, 2	1108, 8	582, 4
O14944	EPIREGULIN	**EREG**	**25**	**1, 8E-06**	**-3, 1**	**17, 1**	**4, 0**	**8, 7**	*-2, 0*	0, 8984	2, 8	1, 3	2, 0	4, 5
P22894	MATRIX METALLOPEPTIDASE 8 (NEUTROPHIL COLLAGENASE)	**MMP8**	**22.2**	**1, 3E-03**	**2, 3**	**51, 3**	**5, 7**	**48, 1**	*1, 5*	0, 4560	10, 6	15, 9	9, 7	18, 5
Q00604	NORRIE DISEASE (PSEUDOGLIOMA)	**NDP**	**19.5**	**3, 8E-07**	**15, 1**	**295, 4**	**13, 4**	**165, 2**	*1, 3*	0, 9185	1, 5	1, 9	1, 6	2, 5
P07357	COMPLEMENT COMPONENT 8, ALPHA POLYPEPTIDE	**C8A**	**18.6**	**3, 6E-04**	**18, 6**	**345, 7**	**21, 4**	**179, 7**	*-1, 6*	0, 5130	780, 6	481, 8	421, 3	165, 0
P78423	FRACTALCINE	**CX3CL1**	**6.4**	**5, 6E-07**	**97, 1**	**617, 8**	**29, 9**	**285, 0**	*9.9*	0, 6235	23.4	232.5	14.3	431.9
P01375	TUMOR NECROSIS FACTOR (TNF SUPERFAMILY, MEMBER 2)	**TNF**	**6**	**3, 5E-10**	**28, 8**	**173, 4**	**5, 5**	**30, 3**	*1, 6*	0, 9980	7, 8	12, 8	4, 4	7, 5
Q9UHD0	INTERLEUKIN 19	**IL19**	**5.6**	**9, 7E-06**	**6, 2**	**34, 9**	**3, 0**	**13, 1**	*1, 8*	0, 5200	-2, 0	-3, 4	3, 0	4, 8
P26022	PENTRAXIN-RELATED GENE, RAPIDLY INDUCED BY IL-1 BETA	**PTX3**	**4**	**1, 0E-04**	**992, 2**	**4000, 0**	**614, 4**	**1504, 4**	*2, 1*	0, 6562	7, 5	15, 5	7, 2	7, 8
P03956	MATRIX METALLOPEPTIDASE 1 (INTERSTITIAL COLLAGENASE)	**MMP1**	**3.4**	**3, 0E-04**	**2662, 1**	**8995, 1**	**3099, 1**	**1471, 0**	*1, 0*	0, 5981	928, 4	968, 5	1473, 1	441, 9
Q9BY76	ANGIOPOIETIN-LIKE 4	**ANGPTL4**	**3.2**	**2, 9E-03**	**83, 8**	**268, 7**	**45, 7**	**139, 2**	*2, 0*	0, 5980	70, 6	137, 9	34, 0	156, 1
P19875	CHEMOKINE (C-X-C MOTIF) LIGAND 2	**CXCL2**	**3.1**	**7, 3E-04**	**1753, 9**	**5471, 3**	**1121, 4**	**1569, 5**	*1, 4*	0, 4725	13, 7	19, 1	5, 3	5, 8
P05121	SERPIN PEPTIDASE INHIBITOR/PLASMINOGEN ACTIVATOR INHIBITOR TYPE 1) MEMBER 1	**SERPINE1/** **pai1**	**3**	**6, 1E-04**	**3110, 0**	**9426, 1**	**2165, 8**	**2364, 8**	*1, 7*	0, 7450	64, 6	108, 5	29, 1	81, 8
P10124	PROTEOGLYCAN 1, SECRETORY GRANULE	**SRGN**	**2.7**	**3, 2E-05**	**3141, 4**	**8426, 4**	**1107, 6**	**2008, 1**	*1, 1*	1, 0000	3, 4	3, 6	3, 0	1, 5
Q96RQ9	INTERLEUKIN 4 INDUCED 1	**IL4I1**	**2.4**	**2, 4E-03**	**132, 4**	**318, 9**	**73, 6**	**149, 7**	*-1, 6*	0, 7500	-1, 2	-0, 8	3, 5	5, 1
Q9Y5U4	INSULIN INDUCED GENE 2	**INSIG2**	**2.3**	**8, 5E-04**	**316, 7**	**722, 0**	**129, 6**	**225, 9**	*-1, 2*	0, 8880	94, 3	79, 9	38, 4	11, 1
P12643	BONE MORPHOGENETIC PROTEIN 2	**BMP2**	**2.2**	**3, 4E-04**	**1131, 3**	**2465, 7**	**472, 0**	**655, 5**	*1, 7*	0, 6915	91, 6	158, 3	103, 2	87, 3
P02735	SERUM AMYLOID A1	**SAA1**	**2.2**	**2, 2E-03**	**355, 1**	**778, 1**	**165, 9**	**246, 5**	*1, 1*	0, 9120	4896, 1	5144, 0	564, 6	238, 5
Q07325	CHEMOKINE (C-X-C MOTIF) LIGAND 9	**CXCL9**	*1.8*	5, 5E-02	207, 1	376, 4	231, 9	233, 9	**69.3**	**0, 0009**	**33, 0**	**2288, 5**	**66, 7**	**3090, 5**
P19876	CHEMOKINE (C-X-C MOTIF) LIGAND 3	**CXCL3**	*3.6*	9, 6E-03	15, 5	56, 0	7, 9	29, 7	**20.6**	**0, 0050**	**28, 1**	**587, 5**	**8, 9**	**300, 0**
O95633	FOLLISTATIN-LIKE 3 (SECRETED GLYCOPROTEIN)	**FSTL3**	*1*	9, 3E-01	687, 0	665, 4	224, 2	136, 2	**15.1**	**0, 0036**	**32, 8**	**495, 5**	**86, 8**	**378, 1**
Q13113	PDZK1 INTERACTING PROTEIN 1	**PDZK1IP1**	*1.9*	2, 3E-01	43, 0	84, 2	25, 9	54, 7	**12.9**	**0, 0010**	**13, 0**	**169, 4**	**51, 6**	**168, 3**
Q9NRD8	DUAL OXIDASE 2	**DUOX2**	*-2*	3, 5E-01	4, 9	2, 3	2, 9	3, 8	**5.5**	**0, 0084**	**77, 2**	**428, 5**	**49, 2**	**154, 2**
Q8WWX9	SELENOPROTEIN M	**SELM**	*1.6*	5, 2E-03	2336, 5	3877, 9	840, 8	945, 4	**3.9**	**0, 0025**	**114, 1**	**445, 5**	**37, 0**	**79, 2**
O94808	GLUTAMINE-FRUCTOSE-6-PHOSPHATE TRANSAMINASE 2	**GFPT2**	*1.7*	1, 3E-04	2797, 3	4808, 3	419, 1	945, 3	**3.6**	**0, 0019**	**15, 1**	**54, 6**	**3, 3**	**2, 7**
P13164	INTERFERON INDUCED TRANSMEMBRANE PROTEIN 1 (9-27)	**IFITM1**	*-1.1*	7, 3E-01	2134, 1	1986, 2	785, 1	682, 9	**3.5**	**0, 0435**	**375, 2**	**1318, 2**	**462, 2**	**466, 0**
P09603	COLONY STIMULATING FACTOR 1 (MACROPHAGE)	**CSF1**	*-1.6*	3, 0E-02	128, 4	73, 4	53, 5	28, 0	**3.3**	**0, 0056**	**73, 4**	**242, 7**	**19, 3**	**118, 7**
P12544	GRANZYME A	**GZMA**	*-1.4*	2, 2E-01	123, 1	85, 7	55, 7	25, 5	**3.3**	**0, 0215**	**6, 7**	**21, 9**	**3, 6**	**27, 1**
P25774	CATHEPSIN S	**CTSS**	*1.4*	1, 2E-02	135, 5	194, 8	43, 5	31, 0	**3.2**	**0, 0168**	**270, 5**	**881, 9**	**86, 8**	**355, 9**
P24001	INTERLEUKIN 32	**IL32**	*1.6*	5, 0E-06	551, 7	888, 2	42, 1	121, 3	**3**	**0, 0061**	**2723, 6**	**8331, 0**	**668, 1**	**472, 2**
P31431	SYNDECAN 4 (AMPHIGLYCAN, RYUDOCAN)	**SDC4**	*1.9*	1, 5E-02	1049, 2	1962, 3	453, 7	796, 4	**2.7**	**0, 0111**	**1141, 2**	**3088, 8**	**160, 0**	**643, 0**
P03973	SECRETORY LEUKOCYTE PEPTIDASE INHIBITOR	**SLPI**	*-1.4*	1, 0E+00	1063, 8	755, 9	946, 2	387, 2	**2.7**	**0, 0050**	**1505, 1**	**4153, 1**	**255, 9**	**660, 6**
P09237	MATRIX METALLOPEPTIDASE 7 (MATRILYSIN, UTERINE)	**MMP7**	*-1.6*	4, 2E-01	7, 9	4, 5	13, 2	15, 1	**2.6**	**0, 0244**	**8, 7**	**22, 8**	**12, 3**	**34, 0**
Q5VY09	IMMEDIATE EARLY RESPONSE 5	**IER5**	*1.6*	4, 7E-03	471, 5	780, 8	99, 5	258, 3	**2.5**	**0, 0215**	**139, 5**	**347, 3**	**79, 7**	**90, 2**
O75976	CARBOXYPEPTIDASE D	**CPD**	*1.3*	1, 2E-01	761, 1	1012, 4	282, 7	315, 0	**2.2**	**0, 0335**	**54, 8**	**118, 9**	**47, 3**	**51, 3**

Fold change (FC) for the highest and significantly upregulated genes (p ≤ 0.05) upon LPS treatment (+LPS) compared to control (-LPS) in adipose tissue (AT), n = 7 and liver tissue (LT) n = 5 are indicated in bold and are underlined. FCs for not significantly changed genes in both tissues (p > 0.05) are in italics. Additionally, information about average (avg) gene expression value and it's standard deviation (std) for both AT and LT is given.

### Transcriptomics and proteomics data comparison and candidate biomarkers identification

In order to validate biomarkers related to inflamed adipose tissue, we performed a similar experiment using a quantitative proteomics approach (CILAIR), and analyzed the secreted proteins in the adipose tissue culture media (secretome). In the CILAIR experiment we identified 192 proteins with incorporated label in medium of LPS treated tissue and 209 in medium of untreated adipose tissue. 178 proteins had incorporated label in both conditions and could thus be compared quantitatively. The statistical analysis revealed that 23 proteins were significantly changed in abundance in the secretome by LPS treatment. Comparison with the gene expression data for adipose tissue showed excellent correlation between proteomics and transcriptomics data (Pearson's correlation r^2 ^= 0.78; Table 2). Within the 23 significantly affected proteins we selected those which were significantly affected by LPS in adipose tissue, on both gene and protein level, but not changed in the liver tissue transcriptome, and those proteins were considered as the best candidate biomarkers for inflamed adipose tissue. We propose: LIF, PTX3, MMP1, SERPINE1, and CX3CL1 as the top candidate biomarkers related to the inflamed adipose tissue. The results are summarized in Table 2.

**Table 2:** Significantly changed secreted proteins in adipose tissue culture media and the corresponding identified genes in adipose tissue and liver tissue upon LPS-treatment

NAME	SYMBOL	AT FC-protein	avg AT LPS+ protein	avg AT LPS- protein	std AT LPS+ protein	std AT LPS- protein	AT (p val.) FC- transcriptome	LT (p val.) FC- transcriptome
**Leukemia inhibitory factor**	**LIF**	**2.3**	6, 6	2, 9	0, 22	0, 41	**(0, 000) 7, 2**	*(ns) 2.9*
**Fractalkine**	**CX3CL1**	**4.3**	4, 3	1, 0	0, 24	0, 10	**(0, 000) 6.4**	*(ns) 9, 9*
**Tumor necrosis factor**	**TNF**	**3.8**	4, 8	1, 3	0, 95	0, 15	**(0, 000) 6**	*(ns) 1.7*
Plasminogen activator inhibitor 2	SERPINB2	**3.1**	2, 6	0, 8	0, 41	0, 05	**(0, 000) 6**	**(0, 002) 8**
Interleukin-6	IL6	**1.6**	6, 9	4, 7	0, 31	0, 07	**(0, 000) 4.5**	**(0, 01) 17.8**
**Pentraxin-related protein**	**PTX3**	**1.9**	3, 5	1, 8	0, 70	0, 14	**(0, 000) 4**	*(ns)2.1*
**Interstitial collagenase**	**MMP1**	**1.7**	4, 7	2, 7	0, 16	0, 17	**(0, 000) 3.4**	*(ns)-1.1*
Tumor necrosis factor-inducible gene 6 protein	TNFAIP6	**5.4**	4, 8	0, 9	0, 95	0, 20	**(0, 000) 3.1**	**(0, 000)17.9**
**Plasminogen activator inhibitor 1**	**SERPINE1**	**1.7**	4, 0	2, 5	0, 19	0, 12	**(0, 000) 3**	*(ns)1.7*
C-C motif chemokine 2	CCL2	**6.9**	6, 9	1, 0	0, 11	0, 10	**(0, 000) 2.3**	**(0, 004) 3.7**
CD44 antigen	CD44	**2.4**	0, 3	0, 1	0, 01	0, 02	*(ns) 1.7*	**(0, 000) 5.8**
Insulin-like growth factor-binding protein 4	IGFBP4	**-2.5**	1, 4	3, 6	0, 10	0, 26	*(ns)-1.1*	*(ns)1.2*
Adipocyte enhancer-binding protein 1	AEBP1	**-1.4**	0, 2	0, 2	0, 00	0, 01	*(ns)-1.2*	*(ns)1.2*
Cystatin-C	CST3	**-3.1**	0, 8	2, 6	0, 06	0, 17	*(ns)-1.2*	*(ns) -1.1*
Versican core protein	VCAN	**-2**	0, 1	0, 2	0, 01	0, 01	*(ns)-1.6*	*(ns) -1.4*
Collagen alpha-1(VI) chain	COL6A1	**-3.3**	0, 4	1, 3	0, 27	0, 18	*(ns)-1.6*	*(ns) -1.4*
Transforming growth factor-beta-induced protein ig-h3	TGFBI	**-2.5**	0, 1	0, 1	0, 00	0, 01	*(ns)-1.6*	*(ns) 1*
Legumain	LGMN	**-3.1**	0, 9	2, 7	0, 06	0, 17	**(0, 002)-2**	*(ns) 1.2*
Gelsolin	GSN	**-2.5**	0, 1	0, 4	0, 03	0, 02	**(0.002)-2**	*(ns) 1.1*
Cathepsin B	CTSB	**-1.2**	2, 2	2, 7	0, 04	0, 04	***(ns) -2***	*(ns) -1.2*
Lysozyme C	LYZ	**-3.3**	0, 1	0, 3	0, 01	0, 04	**(0, 001) -2.5**	*(ns) -3.3*
Alpha-2-macroglobulin	A2M	**-3.3**	0, 3	1, 1	0, 04	0, 06	**(0, 000) -3.3**	*(ns) -1.6*

Fold change (FC) for the significantly changed proteins (p ≤ 0.05, FC > 1.2) in adipose tissue (AT) n = 1 and the significantly changed genes (p ≤ 0.05, FC > 2, n = 7 for adipose tissue and n = 5 for liver tissue (LT)) is represented in bold, p value is given in brackets (...). The insignificantly affected genes (ns) are depicted in italics. FC was calculated in LPS treated samples (LPS+) compared to control samples (LPS-). Additionally, information about average (avg) value in proteomics experiment representative for protein expression and its standard deviation (std) are depicted in the table. The top candidate biomarkers related to inflamed adipose tissue are depicted in bold and underlined.

## Discussion

In the present study we evoked LPS induced inflammation in adipose and liver tissues *in vitro *in order to mimic IR caused by inflammation *in vivo*. We aimed to compare the changes in the inflamed transcriptomes and secretomes of both tissues in order to (1) better understand contribution of the inflamed adipose and liver tissues to the development of insulin resistance and (2) to identify candidate biomarkers indicative for tissue specific inflammation/IR.

**The gene functional classification analysis **revealed that both adipose and liver tissue share common response mechanisms that are activated during inflammation (chemokine signaling, (anti)apoptosis, extracellular matrix remodelling, adhesion and migration of different immune cells involved in inflammatory reactions). Although functional clustering led to identification of the same functional groups, both tissues had a different set of genes within one functional group, suggesting tissue-specific inflammatory signaling. The significantly upregulated adipose tissue transcriptome contained additional gene functional categories belonging to SOCS and several transporters (Additional file 1, Table S1). The SOCS signaling was shown previously to be involved in induction of insulin resistance during acute inflammation in human adipose tissue [23] and our *ex vivo *data are in line with these *in vivo *findings. The analysis of the down regulated functional groups pointed out towards redox/detoxification processes affected in both tissues and mitochondrial functions observed in liver tissue. These processes could contribute to the enhanced reactive oxygen species (ROS) production recognized as one of the mechanisms implicated in the development of IR/T2D [13]. Furthermore, adipose tissue had downregulated genes involved in the extracellular matrix activity which is involved in multiple processes including modulation of immune responses. In liver tissue downregulation of genes involved in amino acid metabolism and polysaccharide binding were observed. There are reports about changed amino acids concentrations in animal models of obesity and obese humans [24,25], however interpretation of this *ex vivo *finding in relation to these reports is not unequivocal.

**The additional network identification **for the common (overlapping) and differential adipose and liver tissue transcriptomes was in line with the data obtained from the gene functional analysis and distinguished the common and differential networks. Moreover, several of these networks were described previously in the literature for their role in induction of IR thereby supporting our model system to study the inflammation related insulin resistance *in vivo*. For example, in our study we found upregulated chemokine signaling and matrix remodelling in both adipose and liver tissues which were also previously linked to the development of IR in vivo [26,27]. SOCS signaling is implicated in induction of IR [28-30] and similarly it was found by us to be upregulated by LPS in adipose tissue *ex vivo*. The decreased PPARγ expression in adipose tissue is recognized as one of the events associated with IR and occurred in our ex vivo studies as well [31,32]. Similarly upregulated Jak-STAT and NFκB signaling identified previously in IR liver [33] was present in our experiments.

**The GO analysis and gene count **revealed that adipose tissue had more LPS-induced upregulated GO terms and genes related primarily to "inflammation", "angiogenesis", and "development". Moreover, the predicted secretome studies showed that the adipose tissue predicted inflammatory secretome is more abundant compared to the liver tissue secretome. This observation indicates that adipose tissue is more active during inflammation, compared to liver tissue, and supports the hypothesis that adipose tissue plays the major role in the development of inflammation-related IR [2].

The reason for different responses of the adipose and liver tissues could be due to a different expression of TLR4 and other components involved in signal transduction via TLR4 (LBP, CD14, TREM1), but unfortunately in our studies we can not directly compare expression values between the adipose tissue and the liver data (two different DNA microarray platforms were used). Nevertheless, we observed that the expression patterns/ratios of all the TLR4 signaling molecules in both tissues were very similar (data not shown).

### The predicted secretome analysis

The microarray data analysis of both tissues revealed that adipose and liver tissues have numerous overlapping LPS-responsive genes which protein products are predicted to be secreted. Among these genes we identified several known markers associated with insulin resistance such as IL-6, IL-1β, IL-8, and PAI 1. Other proteins known to be upregulated during insulin resistance by adipose tissue [34] such as RANTES, MCP1, PLAUR, CXCL5, were found in our studies to be upregulated in both adipose- and liver tissues. Additionally, in both tissues we found genes, previously shown to be regulated in adipose tissue in relation to insulin resistance: CXCL1, CXCL10, CXCL11, ICAM1, TNFAIP6 [35], FGF2, IL6 [32], and ICAM1, IL-1 [36]. Although TNFα is known to be involved in the development of insulin resistance in both adipose tissue and the liver, it was only significantly upregulated in adipose tissue. However, we observed that 3 out of 5 livers had upregulated expression of TNFα and previously we showed that in liver tissue *in vitro*, TNFα mRNA level was significantly upregulated after 5 hrs while after 24hrs the TNFα mRNA level returned to basal values [15,37]. In order to explain this phenomenon we hypothesized that the TNFα response after LPS treatment could be related to number of Kupffer cells (assessed by CD68 expression) or to the expression of TLR4. Thereby, we looked at correlations between TNFα expression and both CD 68 and TLR4. There was no correlation between TNFα expression and CD68, R^2 ^= 0.0063 (data not shown). The correlation between TNFα and TLR4 indicated on a good positive correlation (R^2 ^= 0.4) between these genes and it could indeed explain the observed differences (data not shown).

Furthermore, the comparative analysis of adipose and liver tissues secretomes *in vitro *provides a source of candidate biomarkers related to tissue specific inflammation/insulin resistance. Similarly to Shah et al. [35], we identified in the inflamed adipose tissue secretome genes such as: SELE, CD274, ORM1, PLA1A, SLAMF1, CX3CL1, OSM, TNF, C19ORF59, PTX3, IER3, CCL8, CXCL2, SERPINE1, BMP2, FAM107A, GPX3. Moreover, we identified genes of yet unknown functions such as: C14ORF162, C20ORF59 or genes implicated in other than insulin resistance inflammatory diseases: epiregulin, IL-19 or sarcoglycan [38-40].

The analysis of the predicted secretome of inflamed liver tissue revealed several significantly changed genes with a known- and an unknown- relationship to insulin resistance. Identification of biomarkers indicative for inflamed liver tissue could be a useful tool in a diagnosis of NAFLD patients, where the only "golden standard" is an invasive liver biopsy [41]. Biomarkers previously associated with liver diseases and identified in our samples were among others: ANGPTL3, IGFBP2, SDC4, IL1RN [7,42]. Examples of other pro-inflammatory proteins affiliated with inflammation but not liver insulin resistance were cathepsin S [34] or granzyme A [43]. In future it has to be validated if the other most differentially regulated genes between both tissues such as: SGCD, LCE3D, EREG, NDP and CXCL9, FSTL3, PDZK1IP1 could be used as biomarkers related to insulin resistance of adipose or liver tissues respectively.

### Comparison of transcriptomics and proteomics data

Finally, the transcriptome data encoding for the adipose tissue inflammatory secretome was validated and compared with the protein data of the inflamed adipose tissue culture medium. The analysis showed that the transcriptome data were in line with the proteomics data, in respect to observed upwards and downwards fold changes (FC) for genes and their corresponding protein products. However, the FC derived from the proteomics experiment cannot be directly compared with the FC of the transcriptome experiment due to substantial technical differences between both technologies. By combination of the comparative transcriptome analysis and proteomics technology we identified leukemia inhibitory factor (LIF), matrix metalopeptidase-1(MMP-1), pentraxin related gene product (PTX3), fractalkine (CX3CL1), and PAI 1 as the potential set of biomarkers for the inflamed adipose tissue.

Chronic LIF exposure in cardiomyocytes was linked to insulin resistance [44], however the role of LIF in induction of IR in adipose tissue is not known. Proteins of the matrix metalloproteinase (MMP) family are involved in the breakdown of extracellular matrix in normal physiological processes, such as embryonic development, reproduction, and tissue remodelling, as well as in disease processes, such as arthritis and metastasis [45-47]. MMP1 was not reported earlier as a biomarker of IR/T2D and its role in adipose tissue is not known.

Pentraxin related gene (PTX3) plays a role in innate immunity, inflammation, vascular integrity, fertility, pregnancy, and also in the central nervous system. The PTX3 could influence the development of autoimmune reactions and vascular disorders in humans [48,49]. Recently pentraxin was also associated with obesity and metabolic syndrome [50,51] and it was shown to be secreted by adipocytes [52].

Moreover, very recently CX3CL1 (fractalkine) was proposed as a novel human adipochemokine associated with T2D in humans [53].

Other proposed by us candidate biomarkers such as TNFα and SERPINE 1 (PAI) are commonly associated with inflammation, IR, and T2D and are known to be secreted by the stromal vascular fraction of adipose tissue [54].

In summary, based on the obtained data we postulate that during inflammation related to IR the target peripheral tissues (adipose tissue, liver) secret a set of unique proteins which could serve as tissue-specific biomarkers related to the investigated pathology. We believe that our approach of using multiple biomarkers could result in more specific diagnosis for a tissue specific insulin resistance related to inflammation, than the use of single biomarkers.

One of the shortcomings of our study is the use of two different DNA microarray platforms, since the data used here were generated in two different laboratories. However, previous studies comparing human Affymetrix and Illumina platforms show that the obtained results, using the same human material, are highly comparable, especially for genes which are predicted to be differentially expressed [55]. Furthermore, in our studies we compared only genes which were significantly affected and present on both platforms; therefore genes which were not present on both platforms were excluded from the analysis and we did not compare intensities of corresponding genes since they would be different due to the platform specific design. Another possible disadvantage of our studies is application of patients with different gender (the adipose tissue was obtained form females and the liver was derived form males), BMI, age, and other anthropometric and biochemical parameters. However, due to limited access to human tissues we could not control all the parameters according to the proper experimental design. Nevertheless, we are confident that the results presented provide a good basis for future in vivo validation studies.

## Conclusions

In summary, our *in vitro *approach showed that LPS-induced inflammation in adipose and liver tissues, results in upregulation of inflammatory processes and downregulation of metabolic pathways and redox/detoxification reactions. These processes could synergistically contribute to the deregulation of energy homeostasis leading to insulin resistance. Furthermore, our study implies that adipose tissue is more active during inflammation compared to the liver, based on identification of higher number of GO terms and genes involved in inflammation and angiogenesis, and a number of genes predicted to encode for secreted proteins. Whether the identified tissue-specific molecular pathways and the identified candidate biomarkers can be used for tissue-specific diagnosis of insulin resistance in patients awaits further validated *in vivo*. We believe that this approach may facilitate more targeted treatment of insulin resistance.

## Competing interests

The authors declare that they have no competing interests.

## Authors' contributions

ESZ designed the study, wrote the manuscript, performed the statistical analysis, performed adipose tissue culture and adipose tissue and liver tissue processing, MD performed statistical analysis, developed bioinformatics tools, MGLE performed liver tissue slices culture, was involved in data analysis and manuscript revision, DW was involved in proteomics experiments and adipose tissue culture, MdV performed mass spectrometry analysis for protein identification, MB performed Illumina DNA microarrays and was involved in Illumina data processing, AH provided the human adipose tissue, HR was involved in the CILAIR experiment, GMMG revised the manuscript and was involved in the experiments preformed with human liver slices, RJV revised the manuscript. All authors read and approved the final manuscript.

## Pre-publication history

The pre-publication history for this paper can be accessed here:

http://www.biomedcentral.com/1755-8794/4/71/prepub

## Supplementary Material

Additional file 1 **Gene functional classification**. Table S1. **Gene functional classification-the upregulated adipose tissue transcriptome**. Gene functional classification for the upregulated transcripts in adipose tissue (n = 7) based on the Database for Annotation, Visualization, and Integrated Discovery (DAVID). Table S2. **Gene functional classification-the downregulated adipose tissue transcriptome**. Gene functional classification for the downregulated transcripts in adipose tissue (n = 7) based on the Database for Annotation, Visualization, and Integrated Discovery (DAVID). Table S3. **Gene functional classification-the upregulated liver tissue transcriptome**. Gene functional classification for the upregulated transcripts in liver tissue (n = 5) based on the Database for Annotation, Visualization, and Integrated Discovery (DAVID). Table S4. **Gene functional classification-the downregulated liver tissue transcriptome**. Gene functional classification for the downregulated transcripts in liver tissue (n = 5) based on the Database for Annotation, Visualization, and Integrated Discovery (DAVID). Click here for file

Additional file 2 **GO analysis**. Table S1. **GO analysis for the upregulated adipose tissue transcriptome**. The significantly upregulated GO terms in adipose tissue (n = 7) identified by DAVID. The GO terms were categorized into broader GO categories such as: inflammation, development, signaling, metal ion homeostasis, secretion, and angiogenesis. Table S2. **GO analysis for the downregulated adipose tissue transcriptome**. The significantly downregulated GO terms in adipose tissue (n = 7) identified by DAVID. The GO terms belonged to GO category extracellular matrix. Table S3. **GO analysis for the upregulated liver tissue transcriptome**. The significantly upregulated GO terms in liver tissue (n = 5) identified by DAVID. The GO terms were categorized into broader GO categories such as: inflammation, development, and angiogenesis. Table S4. GO analysis for the downregulated liver tissue transcriptome. The significantly downregulated GO terms in liver tissue (n = 5) identified by DAVID. The GO terms belonged to GO categories: amino acid metabolism and inflammation/binding. Click here for file

Additional file 3 **Gene count analysis for the identified GO categories**. Table S1. **Gene count analysis for the identified GO categories in the significant adipose tissue transcriptome**. The significantly upregulated (up) and downregulated (down) genes in adipose tissue (AT), (n = 7) within the defined GO categories (inflammation, development, signaling, metal ion homeostasis, secretion, angiogenesis, and extracellular region. Table S2. **Gene count analysis for the identified GO categories in the significant liver tissue transcriptome**. The significantly upregulated (up) and downregulated (down) genes in liver tissue (LT), (n = 5) within the defined GO categories (inflammation, development, signaling, angiogenesis, amino acid metabolism, and inflammation/binding). Click here for file

Additional file 4 **Secretome prediction**. Table S1. **The common (overlapping) adipose tissue and liver tissue predicted secretome**. The common (overlapping) adipose tissue (AT), (n = 7) and liver tissue (LT), (n = 5) predicted secretome. The presented genes were significantly changed, p ≤ 0.05 in both tissues. In the last two columns fold changes (FC) in AT and LT are given. Table S2. **The adipose tissue predicted secretome**. The adipose tissue (AT) predicted secretome, (n = 7). Genes present in AT were significantly changed (p ≤ 0.05, FC > 2) while the corresponding genes in liver tissue (LT), (n = 5) were not significantly affected (p > 0.05). Table S3. **The liver tissue predicted secretome**. The liver tissue (LT) predicted secretome, n = 5. Genes present in LT were significantly changed (p ≤ 0.05, FC > 2) while the corresponding genes in adipose tissue (AT), (n = 7) were not significantly affected (p > 0.05). Click here for file
