# Supplementary material for: Comparative analysis of the human hepatic and adipose tissue transcriptomes during LPS-induced inflammation leads to the identification of differential biological pathways and candidate biomarkers
Source: BMC Med Genomics. 2011 Oct 6;4:71. doi: 10.1186/1755-8794-4-71 (PMC3196688; doi:10.1186/1755-8794-4-71)
Supplement: Additional file 1 — Gene functional classification. Table S1. Gene functional classification-the upregulated adipose tissue transcriptome. Gene functional classification for the upregulated transcripts in adipose tissue (n = 7) based on the Database for Annotation, Visualization, and Integrated Discovery (DAVID). Table S2. Gene functional classification-the downregulated adipose tissue transcriptome. Gene functional classification for the downregulated transcripts in adipose tissue (n = 7) based on the Database for Annotation, Visualization, and Integrated Discovery (DAVID). Table S3. Gene functional classification-the upregulated liver tissue transcriptome. Gene functional classification for the upregulated transcripts in liver tissue (n = 5) based on the Database for Annotation, Visualization, and Integrated Discovery (DAVID). Table S4. Gene functional classification-the downregulated liver tissue transcriptome. Gene functional classification for the downregulated transcripts in liver tissue (n = 5) based on the Database for Annotation, Visualization, and Integrated Discovery (DAVID). [file 1755-8794-4-71-S1.DOC]

### Additional file 1 – Gene functional classification

Additional file 1, Table S1

**Gene functional classification for the upregulated adipose tissue transcriptome**

| **Functional group** | **Enrichment Score** | **Gene Symbol** | **Full Gene Name** |
| --- | --- | --- | --- |
| chemokine | 12.51 | CCL20 | CHEMOKINE (C-C MOTIF) LIGAND 20 |
|  |  | CCL2 | CHEMOKINE (C-C MOTIF) LIGAND 2 |
|  |  | CCL3 | CHEMOKINE (C-C MOTIF) LIGAND 3 |
|  |  | CX3CL1 | CHEMOKINE (C-X3-C MOTIF) LIGAND 1 |
|  |  | IL8 | INTERLEUKIN 8 |
|  |  | CXCL1 | CHEMOKINE (C-X-C MOTIF) LIGAND 1 |
|  |  | CXCL2 | CHEMOKINE (C-X-C MOTIF) LIGAND 2 |
|  |  | CXCL6 | CHEMOKINE (C-X-C MOTIF) LIGAND 6 |
|  |  | CCL8 | CHEMOKINE (C-C MOTIF) LIGAND 8 |
|  |  | CCL5 | CHEMOKINE (C-C MOTIF) LIGAND 5 |
|  |  | CXCL10 | CHEMOKINE (C-X-C MOTIF) LIGAND 10 |
|  |  | CXCL11 | CHEMOKINE (C-X-C MOTIF) LIGAND 11 |
|  |  | CCL4L2 | CHEMOKINE (C-C MOTIF) LIGAND 4 |
|  |  | CXCL5 | CHEMOKINE (C-X-C MOTIF) LIGAND 5 |
| growth and differentiation of hematopoietic precursors/cytokine | 7.23 | CSF2 | COLONY STIMULATING FACTOR 2 |
|  |  | CSF3 | COLONY STIMULATING FACTOR 3 |
|  |  | IL19 | INTERLEUKIN 19 |
|  |  | IL11 | INTERLEUKIN 11 |
|  |  | LIF | LEUKEMIA INHIBITORY FACTOR |
| (anti)apoptosis | 7.21 | IER3 | IMMEDIATE EARLY RESPONSE 3 |
|  |  | SERPINB2 | SERPIN PEPTIDASE INHIBITOR, CLADE B (OVALBUMIN), MEMBER 2 |
|  |  | TNFRSF6B | TUMOR NECROSIS FACTOR RECEPTOR SUPERFAMILY, MEMBER 6B, DECOY |
|  |  | BCL2A1 | BCL2-RELATED PROTEIN A1 |
| modulation of immune response | 3.8 | SERPINB4 | SERPIN PEPTIDASE INHIBITOR, CLADE B (OVALBUMIN), MEMBER 3 |
|  |  | SERPINA1 | SERPIN PEPTIDASE INHIBITOR, CLADE A (ALPHA-1 ANTIPROTEINASE, ANTITRYPSIN), MEMBER 1 |
|  |  | SERPINB2 | SERPIN PEPTIDASE INHIBITOR, CLADE B (OVALBUMIN), MEMBER 2 |
|  |  | SERPINB7 | SERPIN PEPTIDASE INHIBITOR, CLADE B (OVALBUMIN), MEMBER 7 |
| extracellular matrix modulation(T, B, leukocytes, NK-cells activation ) | 3.54 | GPR64 | G PROTEIN-COUPLED RECEPTOR 64 |
|  |  | ITGA2 | INTEGRIN, ALPHA 2 (CD49B, ALPHA 2 SUBUNIT OF VLA-2 RECEPTOR) |
|  |  | P2RY6 | PYRIMIDINERGIC RECEPTOR P2Y, G-PROTEIN COUPLED, 6 |
|  |  | CD44 | CD44 ANTIGEN (INDIAN BLOOD GROUP) |
|  |  | CD38 | CD38 ANTIGEN (P45) |
|  |  | OSMR | ONCOSTATIN M RECEPTOR |
|  |  | OR4Q3 | OLFACTORY RECEPTOR, FAMILY 4, SUBFAMILY Q, MEMBER 3 |
|  |  | ZP3 | ZONA PELLUCIDA GLYCOPROTEIN 3 (SPERM RECEPTOR) |
|  |  | SLAMF7 | SLAM FAMILY MEMBER 7 |
|  |  | LILRA3 | LEUKOCYTE IMMUNOGLOBULIN-LIKE RECEPTOR, SUBFAMILY A (WITHOUT TM DOMAIN), MEMBER 3 |
|  |  | ITGB8 | INTEGRIN, BETA 8 |
|  |  | CCR7 | CHEMOKINE (C-C MOTIF) RECEPTOR 7 |
|  |  | SLAMF1 | SIGNALING LYMPHOCYTIC ACTIVATION MOLECULE FAMILY MEMBER 1 |
|  |  | IL7R | INTERLEUKIN 7 RECEPTOR |
|  |  | MRGPRX3 | MAS-RELATED GPR, MEMBER X3 |
|  |  | IL15RA | INTERLEUKIN 15 RECEPTOR, ALPHA |
|  |  | TNFRSF1B | TUMOR NECROSIS FACTOR RECEPTOR SUPERFAMILY, MEMBER 1B |
|  |  | IL2RA | INTERLEUKIN 2 RECEPTOR, ALPHA |
|  |  | GPR84 | G PROTEIN-COUPLED RECEPTOR 84 |
|  |  | EDG2 | ENDOTHELIAL DIFFERENTIATION, LYSOPHOSPHATIDIC ACID G-PROTEIN-COUPLED RECEPTOR, 2 |
|  |  | ICAM1 | INTERCELLULAR ADHESION MOLECULE 1 (CD54), HUMAN RHINOVIRUS RECEPTOR |
|  |  | LRIG1 | LEUCINE-RICH REPEATS AND IMMUNOGLOBULIN-LIKE DOMAINS 1 |
|  |  | CD82 | CD82 ANTIGEN |
|  |  | PANX1 | PANNEXIN 1 |
|  |  | CD48 | CD48 ANTIGEN (B-CELL MEMBRANE PROTEIN) |
|  |  | KREMEN1 | KRINGLE CONTAINING TRANSMEMBRANE PROTEIN 1 |
|  |  | LTB | LYMPHOTOXIN BETA (TNF SUPERFAMILY, MEMBER 3) |
|  |  | IER3 | IMMEDIATE EARLY RESPONSE 3 |
|  |  | MPZL1 | MYELIN PROTEIN ZERO-LIKE 1 |
|  |  | DKFZP564O0823 | DKFZP564O0823 PROTEIN |
|  |  | C6ORF128 | CHROMOSOME 6 OPEN READING FRAME 128 |
|  |  | C19ORF59 | MAST CELL-EXPRESSED MEMBRANE PROTEIN 1 |
| regulation of cytokine signaling | 3.16 | SOCS3 | SUPPRESSOR OF CYTOKINE SIGNALING 3 |
|  |  | OSM | ONCOSTATIN M |
|  |  | CLCF1 | CARDIOTROPHIN-LIKE CYTOKINE FACTOR 1 |
|  |  | SOCS1 | SUPPRESSOR OF CYTOKINE SIGNALING 1 |
| apoptosis | 2.91 | CASP4 | CASPASE 4, APOPTOSIS-RELATED CYSTEINE PEPTIDASE |
|  |  | CASP5 | CASPASE 5, APOPTOSIS-RELATED CYSTEINE PEPTIDASE |
|  |  | CRADD | CASP2 AND RIPK1 DOMAIN CONTAINING ADAPTOR WITH DEATH DOMAIN |
|  |  | IL19 | INTERLEUKIN 19 |
| extracellular matrix remodeling | 2.11 | MMP1 | MATRIX METALLOPEPTIDASE 1 (INTERSTITIAL COLLAGENASE) |
|  |  | MMP8 | MATRIX METALLOPEPTIDASE 8 (NEUTROPHIL COLLAGENASE) |
|  |  | MMP3 | MATRIX METALLOPEPTIDASE 3 (STROMELYSIN 1, PROGELATINASE) |
|  |  | MMP12 | MATRIX METALLOPEPTIDASE 12 (MACROPHAGE ELASTASE) |
| (aa) transporters | 2.02 | SLC1A2 | SOLUTE CARRIER FAMILY 1 (GLIAL HIGH AFFINITY GLUTAMATE TRANSPORTER), MEMBER 2 |
|  |  | PANX1 | PANNEXIN 1 |
|  |  | SLC7A2 | SOLUTE CARRIER FAMILY 7 (CATIONIC AMINO ACID TRANSPORTER, Y+ SYSTEM), MEMBER 2 |
|  |  | SLCO4A1 | SOLUTE CARRIER ORGANIC ANION TRANSPORTER FAMILY, MEMBER 4A1 |
|  |  | CD82 | CD82 ANTIGEN |
|  |  | DKFZP564O0823 | DKFZP564O0823 PROTEIN |
|  |  | C20ORF59 | CHROMOSOME 20 OPEN READING FRAME 59 |
|  |  | SLC7A5 | SOLUTE CARRIER FAMILY 7 (CATIONIC AMINO ACID TRANSPORTER, Y+ SYSTEM), MEMBER 5 |
|  |  | SLC7A11 | SOLUTE CARRIER FAMILY 7, (CATIONIC AMINO ACID TRANSPORTER, Y+ SYSTEM) MEMBER 11 |
|  |  | SLC16A3 | SOLUTE CARRIER FAMILY 16 (MONOCARBOXYLIC ACID TRANSPORTERS), MEMBER 3 |
|  |  | SLC2A6 | SOLUTE CARRIER FAMILY 2 (FACILITATED GLUCOSE TRANSPORTER), MEMBER 6 |

Gene functional classification for the upregulated transcripts in adipose tissue (n=7) based on the Database for Annotation, Visualization, and Integrated Discovery (DAVID).

Additional file 1, Table S2

**Gene functional classification for the downregulated adipose tissue transcriptome.**

| **Functional group** | **Enrichment Score** | **Gene Symbol** | **Full Gene Name** |
| --- | --- | --- | --- |
| lysosom/endosomal system activity | 4.01 | DNASE2 | DEOXYRIBONUCLEASE II, LYSOSOMAL |
|  |  | LGMN | LEGUMAIN |
|  |  | CTSB | CATHEPSIN B |
|  |  | ACP5 | ACID PHOSPHATASE 5, TARTRATE RESISTANT |
|  |  | TINAGL1 | TUBULOINTERSTITIAL NEPHRITIS ANTIGEN-LIKE 1 |
|  |  | FUCA1 | FUCOSIDASE, ALPHA-L- 1, TISSUE |
|  |  | IFI30 | INTERFERON, GAMMA-INDUCIBLE PROTEIN 30 |
| extracellular matrix-basement membrane components | 3.8 | COL4A5 | COLLAGEN, TYPE IV, ALPHA 5 (ALPORT SYNDROME) |
|  |  | COL8A1 | COLLAGEN, TYPE VIII, ALPHA 1 |
|  |  | C1QTNF5 | DKFZP586B0621 PROTEIN |
|  |  | EMID1 | EMI DOMAIN CONTAINING 1 |
|  |  | C1QA | COMPLEMENT COMPONENT 1, Q SUBCOMPONENT, A CHAIN |
| extracellular matrix components | 3.16 | GAS6 | GROWTH ARREST-SPECIFIC 6 |
|  |  | EFEMP1 | EGF-CONTAINING FIBULIN-LIKE EXTRACELLULAR MATRIX PROTEIN 1 |
|  |  | FBLN5 | FIBULIN 5 |
|  |  | CD248 | CD248 ANTIGEN, ENDOSIALIN |
|  |  | FBLN1 | FIBULIN 1 |
| Extracellular matrix-cell adhesion and migration | 2.41 | GHR | GROWTH HORMONE RECEPTOR |
|  |  | STAB1 | STABILIN 1 |
|  |  | MSR1 | MACROPHAGE SCAVENGER RECEPTOR 1 |
|  |  | ASTN2 | ASTROTACTIN 2 |
|  |  | IL11RA | INTERLEUKIN 11 RECEPTOR, ALPHA |
|  |  | TREM2 | TRIGGERING RECEPTOR EXPRESSED ON MYELOID CELLS 2 |
|  |  | PTPRF | PROTEIN TYROSINE PHOSPHATASE, RECEPTOR TYPE, F |
|  |  | LRRN2 | LEUCINE RICH REPEAT NEURONAL 5 |
|  |  | AQP1 | AQUAPORIN 1 (COLTON BLOOD GROUP) |
|  |  | CD36 | CD36 ANTIGEN (COLLAGEN TYPE I RECEPTOR, THROMBOSPONDIN RECEPTOR) |
|  |  | STXBP6 | SYNTAXIN BINDING PROTEIN 6 (AMISYN) |
|  |  | MEST | MESODERM SPECIFIC TRANSCRIPT HOMOLOG (MOUSE) |
|  |  | HSD3B7 | HYDROXY-DELTA-5-STEROID DEHYDROGENASE, 3 BETA- AND STEROID DELTA-ISOMERASE 7 |
|  |  | ALDH3A2 | ALDEHYDE DEHYDROGENASE 3 FAMILY, MEMBER A2 |
|  |  | EMCN | ENDOMUCIN |
|  |  | PCDH18 | PROTOCADHERIN 18 |
|  |  | SGCD | SARCOGLYCAN, DELTA (35KDA DYSTROPHIN-ASSOCIATED GLYCOPROTEIN) |
|  |  | EPHA3 | EPH RECEPTOR A3 |
|  |  | MFNG | MANIC FRINGE HOMOLOG (DROSOPHILA) |
|  |  | GPM6A | GLYCOPROTEIN M6A |
|  |  | FPRL2 | FORMYL PEPTIDE RECEPTOR-LIKE 2 |
|  |  | SLCO2B1 | SOLUTE CARRIER ORGANIC ANION TRANSPORTER FAMILY, MEMBER 2B1 |
|  |  | PCDH7 | BH-PROTOCADHERIN (BRAIN-HEART) |
|  |  | C5ORF4 | CHROMOSOME 5 OPEN READING FRAME 4 |
|  |  | CLEC14A | C-TYPE LECTIN DOMAIN FAMILY 14, MEMBER A |
|  |  | SCARB1 | SCAVENGER RECEPTOR CLASS B, MEMBER 1 |
|  |  | PXMP2 | PEROXISOMAL MEMBRANE PROTEIN 2, 22KDA |
|  |  | LYVE1 | EXTRACELLULAR LINK DOMAIN CONTAINING 1 |
|  |  | CD248 | CD248 ANTIGEN, ENDOSIALIN |
|  |  | CD302 | CD302 ANTIGEN |
|  |  | GIMAP1 | GTPASE, IMAP FAMILY MEMBER 1 |
|  |  | PCDH17 | PROTOCADHERIN 17 |
|  |  | FLJ23834 | HYPOTHETICAL PROTEIN FLJ23834 |
|  |  | GPR1 | G PROTEIN-COUPLED RECEPTOR 1 |
|  |  | CCBP2 | CHEMOKINE BINDING PROTEIN 2 |
|  |  | TSPAN7 | TETRASPANIN 7 |
|  |  | GPR34 | G PROTEIN-COUPLED RECEPTOR 34 |
|  |  | GPER | G PROTEIN-COUPLED RECEPTOR 30 |
|  |  | SCARA3 | SCAVENGER RECEPTOR CLASS A, MEMBER 3 |
|  |  | ARL6IP5 | ADP-RIBOSYLATION-LIKE FACTOR 6 INTERACTING PROTEIN 5 |
|  |  | FATE1 | FETAL AND ADULT TESTIS EXPRESSED 1 |
|  |  | PLXDC2 | PLEXIN DOMAIN CONTAINING 2 |
|  |  | FAM26B | FAMILY WITH SEQUENCE SIMILARITY 26, MEMBER B |
|  |  | GPR146 | G PROTEIN-COUPLED RECEPTOR 146 |
|  |  | C20ORF103 | CHROMOSOME 20 OPEN READING FRAME 103 |
|  |  | THSD1 | THROMBOSPONDIN, TYPE I, DOMAIN CONTAINING 1 |
|  |  | MRGPRF | MAS-RELATED GPR, MEMBER F |
|  |  | PLVAP | PLASMALEMMA VESICLE ASSOCIATED PROTEIN |
|  |  | GLT8D2 | GLYCOSYLTRANSFERASE 8 DOMAIN CONTAINING 2 |
|  |  | GPR81 | G PROTEIN-COUPLED RECEPTOR 81 |
|  |  | MS4A6A | CD20-LIKE PRECUSOR |
|  |  | CYYR1 | CYSTEINE/TYROSINE-RICH 1 |
| deoxy-/ribo-nucleases activity | 2.37 | RNASE1 | RIBONUCLEASE, RNASE A FAMILY, 1 (PANCREATIC) |
|  |  | DNASE2 | DEOXYRIBONUCLEASE II, LYSOSOMAL |
|  |  | RNASET2 | RIBONUCLEASE T2 |
|  |  | DNASE1L1 | DEOXYRIBONUCLEASE I-LIKE 1 |
| detoxification | 1.94 | GSTM4 | GLUTATHIONE S-TRANSFERASE M4 |
|  |  | GSTA4 | GLUTATHIONE S-TRANSFERASE A4 |
|  |  | GSTM2 | GLUTATHIONE S-TRANSFERASE M2 (MUSCLE) |
|  |  | GSTM5 | GLUTATHIONE S-TRANSFERASE M5 |

Gene functional classification for the downregulated transcripts in adipose tissue (n=7) based on the Database for Annotation, Visualization, and Integrated Discovery (DAVID).

Additional file 1, Table S3

**Gene functional classification for the upregulated liver tissue transcriptome**

| **Functional group** | **Enrichment Score** | **Gene Symbol** | **Full Gene Name** |
| --- | --- | --- | --- |
| chemokine | 9.75 | CCL20 | CHEMOKINE (C-C MOTIF) LIGAND 20 |
|  |  | CCL2 | CHEMOKINE (C-C MOTIF) LIGAND 2 |
|  |  | CCL3 | CHEMOKINE (C-C MOTIF) LIGAND 3 |
|  |  | IL8 | INTERLEUKIN 8 |
|  |  | CXCL1 | CHEMOKINE (C-X-C MOTIF) LIGAND 1 (MELANOMA GROWTH STIMULATING ACTIVITY, ALPHA) |
|  |  | CXCL3 | CHEMOKINE (C-X-C MOTIF) LIGAND 3 |
|  |  | CXCL6 | CHEMOKINE (C-X-C MOTIF) LIGAND 6 (GRANULOCYTE CHEMOTACTIC PROTEIN 2) |
|  |  | CCL7 | CHEMOKINE (C-C MOTIF) LIGAND 7 |
|  |  | CXCL9 | CHEMOKINE (C-X-C MOTIF) LIGAND 9 |
|  |  | CCL3L3 | CHEMOKINE (C-C MOTIF) LIGAND 3-LIKE 1 |
|  |  | CCL5 | CHEMOKINE (C-C MOTIF) LIGAND 5 |
|  |  | CXCL10 | CHEMOKINE (C-X-C MOTIF) LIGAND 10 |
|  |  | CXCL11 | CHEMOKINE (C-X-C MOTIF) LIGAND 11 |
|  |  | CXCL5 | CHEMOKINE (C-X-C MOTIF) LIGAND 5 |
| matrix remodeling | 3.39 | PI3 | PEPTIDASE INHIBITOR 3, SKIN-DERIVED (SKALP) |
|  |  | SERPINB2 | SERPIN PEPTIDASE INHIBITOR, CLADE B (OVALBUMIN), MEMBER 2 |
|  |  | SLPI | SECRETORY LEUKOCYTE PEPTIDASE INHIBITOR |
|  |  | TFPI2 | TISSUE FACTOR PATHWAY INHIBITOR 2 |
| (anti)apoptosis | 2.73 | SERPINB2 | SERPIN PEPTIDASE INHIBITOR, CLADE B (OVALBUMIN), MEMBER 2 |
|  |  | BCL2L11 | BCL2-LIKE 11 (APOPTOSIS FACILITATOR) |
|  |  | TNFAIP8 | TUMOR NECROSIS FACTOR, ALPHA-INDUCED PROTEIN 8 |
|  |  | BCL2A1 | BCL2-RELATED PROTEIN A1 |
| cell adhesion and migration | 2.36 | P2RY6 | PYRIMIDINERGIC RECEPTOR P2Y, G-PROTEIN COUPLED, 6 |
|  |  | CD44 | CD44 ANTIGEN (INDIAN BLOOD GROUP) |
|  |  | IL7R | INTERLEUKIN 7 RECEPTOR |
|  |  | ZP3 | ZONA PELLUCIDA GLYCOPROTEIN 3 (SPERM RECEPTOR) |
|  |  | ITGB8 | INTEGRIN, BETA 8 |
|  |  | PTGFR | PROSTAGLANDIN F RECEPTOR (FP) |
|  |  | TNFRSF1B | TUMOR NECROSIS FACTOR RECEPTOR SUPERFAMILY, MEMBER 1B |
|  |  | ICAM1 | INTERCELLULAR ADHESION MOLECULE 1 (CD54), HUMAN RHINOVIRUS RECEPTOR |
|  |  | CD47 | CD47 ANTIGEN (RH-RELATED ANTIGEN, INTEGRIN-ASSOCIATED SIGNAL TRANSDUCER) |
|  |  | TPBG | TROPHOBLAST GLYCOPROTEIN |
|  |  | HLA-F | MAJOR HISTOCOMPATIBILITY COMPLEX, CLASS I, F |
|  |  | SDC4 | SYNDECAN 4 (AMPHIGLYCAN, RYUDOCAN) |
|  |  | MPZL1 | MYELIN PROTEIN ZERO-LIKE 1 |
| T cells and NK cells acivity | 2.09 | CTSS | CATHEPSIN S |
|  |  | SERPINB2 | SERPIN PEPTIDASE INHIBITOR, CLADE B (OVALBUMIN), MEMBER 2 |
|  |  | GZMA | GRANZYME A (GRANZYME 1, CYTOTOXIC T-LYMPHOCYTE-ASSOCIATED SERINE ESTERASE 3) |
|  |  | GZMB | GRANZYME B (GRANZYME 2, CYTOTOXIC T-LYMPHOCYTE-ASSOCIATED SERINE ESTERASE 1) |
| breakdown of extracellular matrix/tisssue remodeling | 1.66 | MMP7 | MATRIX METALLOPEPTIDASE 7 (MATRILYSIN, UTERINE) |
|  |  | MMP3 | MATRIX METALLOPEPTIDASE 3 (STROMELYSIN 1, PROGELATINASE) |
|  |  | ADAM17 | ADAM METALLOPEPTIDASE DOMAIN 17 (TUMOR NECROSIS FACTOR, ALPHA, CONVERTING ENZYME) |
|  |  | MMP12 | MATRIX METALLOPEPTIDASE 12 (MACROPHAGE ELASTASE) |

Gene functional classification for the upregulated transcripts in liver tissue (n=5) based on the Database for Annotation, Visualization, and Integrated Discovery (DAVID).

Additional file 1, Table S4

**Gene functional classification for the downregulated liver tissue transcriptome**

| **Functional group** | **Enrichment Score** | **Gene Symbol** | **Full Gene Name** |
| --- | --- | --- | --- |
| aa metabolism | 4.13 | SHMT1 | SERINE HYDROXYMETHYLTRANSFERASE 1 (SOLUBLE) |
|  |  | HAL | HISTIDINE AMMONIA-LYASE |
|  |  | GCSH | GLYCINE CLEAVAGE SYSTEM PROTEIN H (AMINOMETHYL CARRIER) |
|  |  | HIBCH | 3-HYDROXYISOBUTYRYL-COENZYME A HYDROLASE |
| Extracellular matrix-membrane activity | 2.58 | SLC45A3 | SOLUTE CARRIER FAMILY 45, MEMBER 3 |
|  |  | AIG1 | ANDROGEN-INDUCED 1 |
|  |  | PQLC1 | PQ LOOP REPEAT CONTAINING 1 |
|  |  | CLEC4G | C-TYPE LECTIN SUPERFAMILY 4, MEMBER G |
|  |  | TMEM86B | TRANSMEMBRANE PROTEIN 86B |
|  |  | MEST | MESODERM SPECIFIC TRANSCRIPT HOMOLOG (MOUSE) |
|  |  | REEP6 | RECEPTOR ACCESSORY PROTEIN 6 |
| redox/detoxification reactions | 2.19 | CYP3A4 | CYTOCHROME P450, SUBFAMILY IIIA (NIPHEDIPINE OXIDASE), POLYPEPTIDE 3 |
|  |  | RDH16 | RETINOL DEHYDROGENASE 16 (ALL-TRANS AND 13-CIS) |
|  |  | AADAC | ARYLACETAMIDE DEACETYLASE (ESTERASE) |
|  |  | UGT2B15 | UDP GLUCURONOSYLTRANSFERASE 2 FAMILY, POLYPEPTIDE B15 |
| Extracellular matrix-cell adhesion and migration | 2.17 | GPNMB | GLYCOPROTEIN (TRANSMEMBRANE) NMB |
|  |  | ICAM2 | INTERCELLULAR ADHESION MOLECULE 2 |
|  |  | SLC2A2 | SOLUTE CARRIER FAMILY 2 (FACILITATED GLUCOSE TRANSPORTER), MEMBER 2 |
|  |  | TM4SF4 | TRANSMEMBRANE 4 L SIX FAMILY MEMBER 4 |
|  |  | SDC1 | SYNDECAN 1 |
|  |  | RAMP1 | RECEPTOR (CALCITONIN) ACTIVITY MODIFYING PROTEIN 1 |
| mitochondrial functions | 2.01 | CRLS1 | CHROMOSOME 20 OPEN READING FRAME 155 |
|  |  | AKAP1 | A KINASE (PRKA) ANCHOR PROTEIN 1 |
|  |  | C14orf68 | CHROMOSOME 14 OPEN READING FRAME 68 |
|  |  | SFXN3 | SIDEROFLEXIN 3 |

Gene functional classification for the downregulated transcripts in liver tissue (n=5) based on the Database for Annotation, Visualization, and Integrated Discovery (DAVID).
