# Supplementary material for: Comparative analysis of the human hepatic and adipose tissue transcriptomes during LPS-induced inflammation leads to the identification of differential biological pathways and candidate biomarkers
Source: BMC Med Genomics. 2011 Oct 6;4:71. doi: 10.1186/1755-8794-4-71 (PMC3196688; doi:10.1186/1755-8794-4-71)
Supplement: Additional file 2 — GO analysis. Table S1. GO analysis for the upregulated adipose tissue transcriptome. The significantly upregulated GO terms in adipose tissue (n = 7) identified by DAVID. The GO terms were categorized into broader GO categories such as: inflammation, development, signaling, metal ion homeostasis, secretion, and angiogenesis. Table S2. GO analysis for the downregulated adipose tissue transcriptome. The significantly downregulated GO terms in adipose tissue (n = 7) identified by DAVID. The GO terms belonged to GO category extracellular matrix. Table S3. GO analysis for the upregulated liver tissue transcriptome. The significantly upregulated GO terms in liver tissue (n = 5) identified by DAVID. The GO terms were categorized into broader GO categories such as: inflammation, development, and angiogenesis. Table S4. GO analysis for the downregulated liver tissue transcriptome. The significantly downregulated GO terms in liver tissue (n = 5) identified by DAVID. The GO terms belonged to GO categories: amino acid metabolism and inflammation/binding. [file 1755-8794-4-71-S2.DOC]

### Additional file 2 – GO analysis

Additional file 2, Table S1

**GO analysis for the upregulated adipose tissue transcriptome**

| **inflammation** | Fold Enrichment | Benjamini |
| --- | --- | --- |
| GO:0005615~extracellular space | 5.9 | 6.6E-22 |
| GO:0005125~cytokine activity | 8.9 | 5.6E-19 |
| GO:0044421~extracellular region part | 4.4 | 2.5E-19 |
| GO:0005576~extracellular region | 3.4 | 9.7E-17 |
| GO:0006955~immune response | 4.4 | 4.8E-14 |
| GO:0002376~immune system process | 3.7 | 9.7E-14 |
| GO:0005102~receptor binding | 3.9 | 9.5E-13 |
| GO:0007267~cell-cell signaling | 3.7 | 2.8E-10 |
| GO:0006954~inflammatory response | 8.0 | 1.1E-21 |
| GO:0006952~defense response | 5.6 | 8.0E-22 |
| GO:0009611~response to wounding | 6.5 | 5.9E-22 |
| GO:0009605~response to external stimulus | 5.0 | 3.1E-20 |
| GO:0006935~chemotaxis | 9.5 | 2.9E-13 |
| GO:0042330~taxis | 9.5 | 2.9E-13 |
| GO:0006950~response to stress | 3.2 | 7.7E-13 |
| GO:0007626~locomotory behavior | 7.4 | 1.8E-11 |
| GO:0050896~response to stimulus | 2.1 | 2.4E-10 |
| GO:0007610~behavior | 4.8 | 2.5E-08 |
| GO:0042221~response to chemical stimulus | 3.2 | 3.9E-06 |
| GO:0005125~cytokine activity | 8.9 | 5.6E-19 |
| GO:0006935~chemotaxis | 9.5 | 2.9E-13 |
| GO:0042330~taxis | 9.5 | 2.9E-13 |
| GO:0007626~locomotory behavior | 7.4 | 1.8E-11 |
| GO:0008009~chemokine activity | 17.6 | 5.2E-10 |
| GO:0042379~chemokine receptor binding | 17.2 | 5.2E-10 |
| GO:0001664~G-protein-coupled receptor binding | 12.8 | 7.0E-10 |
| GO:0007610~behavior | 4.8 | 2.5E-08 |
| GO:0042221~response to chemical stimulus | 3.2 | 3.9E-06 |
| GO:0001871~pattern binding | 6.2 | 1.5E-03 |
| GO:0030247~polysaccharide binding | 5.7 | 1.8E-02 |
| GO:0005539~glycosaminoglycan binding | 5.3 | 7.1E-02 |
| GO:0007155~cell adhesion | 2.1 | 4.5E-02 |
| GO:0022610~biological adhesion | 2.1 | 4.5E-02 |
| GO:0030595~leukocyte chemotaxis | 12.8 | 3.1E-02 |
| GO:0031347~regulation of defense response | 11.7 | 9.9E-03 |
| GO:0050727~regulation of inflammatory response | 11.7 | 9.9E-03 |
| GO:0048583~regulation of response to stimulus | 8.7 | 3.3E-02 |
| GO:0001775~cell activation | 3.9 | 1.4E-03 |
| GO:0045321~leukocyte activation | 4.1 | 1.6E-03 |
| GO:0051239~regulation of multicellular organismal process | 3.3 | 4.4E-03 |
| GO:0046649~lymphocyte activation | 3.8 | 2.1E-02 |
| GO:0051250~negative regulation of lymphocyte activation | 13.5 | 2.6E-02 |
| GO:0050868~negative regulation of T cell activation | 17.4 | 6.9E-02 |
| GO:0006953~acute-phase response | 10.1 | 6.9E-02 |
| GO:0002526~acute inflammatory response | 5.6 | 7.6E-02 |
| **development** | Fold Enrichment | Benjamini |
| GO:0008219~cell death | 3.1 | 2.0E-08 |
| GO:0016265~death | 3.1 | 2.0E-08 |
| GO:0012501~programmed cell death | 3.1 | 4.1E-08 |
| GO:0048468~cell development | 2.5 | 7.5E-08 |
| GO:0006915~apoptosis | 3.0 | 1.0E-07 |
| GO:0043067~regulation of programmed cell death | 3.5 | 2.2E-07 |
| GO:0042981~regulation of apoptosis | 3.5 | 6.0E-07 |
| GO:0048869~cellular developmental process | 2.1 | 5.8E-07 |
| GO:0030154~cell differentiation | 2.1 | 5.8E-07 |
| GO:0043066~negative regulation of apoptosis | 4.3 | 3.1E-04 |
| GO:0043069~negative regulation of programmed cell death | 4.2 | 3.3E-04 |
| GO:0048523~negative regulation of cellular process | 2.2 | 3.5E-04 |
| GO:0006916~anti-apoptosis | 4.5 | 3.3E-03 |
| GO:0042127~regulation of cell proliferation | 3.5 | 1.6E-06 |
| GO:0048518~positive regulation of biological process | 2.4 | 7.3E-06 |
| GO:0048522~positive regulation of cellular process | 2.5 | 8.0E-06 |
| GO:0008284~positive regulation of cell proliferation | 4.6 | 2.5E-05 |
| GO:0008283~cell proliferation | 2.6 | 3.2E-05 |
| GO:0032502~developmental process | 1.8 | 2.9E-08 |
| GO:0048513~organ development | 2.1 | 3.1E-04 |
| GO:0009653~anatomical structure morphogenesis | 2.2 | 3.1E-04 |
| GO:0048856~anatomical structure development | 1.8 | 3.3E-04 |
| GO:0048731~system development | 1.8 | 2.1E-03 |
| GO:0032501~multicellular organismal process | 1.5 | 3.3E-03 |
| GO:0007275~multicellular organismal development | 1.6 | 1.6E-02 |
| GO:0043067~regulation of programmed cell death | 3.5 | 2.2E-07 |
| GO:0042981~regulation of apoptosis | 3.5 | 6.0E-07 |
| GO:0043065~positive regulation of apoptosis | 2.9 | 8.1E-02 |
| GO:0043068~positive regulation of programmed cell death | 2.9 | 8.5E-02 |
| GO:0035295~tube development | 4.3 | 1.5E-02 |
| **signaling** | Fold Enrichment | Benjamini |
| GO:0007154~cell communication | 1.7 | 4.1E-07 |
| GO:0007165~signal transduction | 1.6 | 1.5E-05 |
| GO:0007166~cell surface receptor linked signal transduction | 1.7 | 6.1E-03 |
| GO:0065007~biological regulation | 1.5 | 3.6E-05 |
| GO:0050789~regulation of biological process | 1.4 | 6.0E-04 |
| GO:0050794~regulation of cellular process | 1.4 | 1.6E-02 |
| GO:0060089~molecular transducer activity | 1.7 | 1.5E-02 |
| GO:0004871~signal transducer activity | 1.7 | 1.5E-02 |
| **metal ion homeostasis** | Fold Enrichment | Benjamini |
| GO:0065008~regulation of biological quality | 2.9 | 3.5E-07 |
| GO:0055074~calcium ion homeostasis | 6.2 | 1.6E-04 |
| GO:0006874~cellular calcium ion homeostasis | 6.2 | 1.6E-04 |
| GO:0055065~metal ion homeostasis | 5.8 | 3.1E-04 |
| GO:0006875~cellular metal ion homeostasis | 5.8 | 3.1E-04 |
| GO:0030005~cellular di-, tri-valent inorganic cation homeostasis | 4.9 | 1.4E-03 |
| GO:0055066~di-, tri-valent inorganic cation homeostasis | 4.9 | 1.5E-03 |
| GO:0030003~cellular cation homeostasis | 4.3 | 4.3E-03 |
| GO:0055080~cation homeostasis | 4.3 | 4.4E-03 |
| GO:0055082~cellular chemical homeostasis | 3.9 | 5.2E-03 |
| GO:0006873~cellular ion homeostasis | 3.9 | 5.2E-03 |
| GO:0007204~elevation of cytosolic calcium ion concentration | 6.9 | 1.1E-02 |
| GO:0051480~cytosolic calcium ion homeostasis | 6.9 | 1.1E-02 |
| GO:0050801~ion homeostasis | 3.5 | 1.1E-02 |
| GO:0042592~homeostatic process | 2.7 | 1.3E-02 |
| GO:0019725~cellular homeostasis | 3.1 | 1.3E-02 |
| GO:0048878~chemical homeostasis | 3.2 | 1.4E-02 |
| **secretion** | Fold Enrichment | Benjamini |
| GO:0051046~regulation of secretion | 9.0 | 2.0E-04 |
| GO:0051048~negative regulation of secretion | 19.9 | 9.0E-04 |
| GO:0046888~negative regulation of hormone secretion | 23.6 | 3.7E-03 |
| GO:0003001~generation of a signal involved in cell-cell signaling | 5.9 | 2.4E-02 |
| GO:0046883~regulation of hormone secretion | 12.8 | 3.1E-02 |
| GO:0046879~hormone secretion | 8.7 | 3.3E-02 |
| **angiogenesis** | Fold Enrichment | Benjamini |
| GO:0048514~blood vessel morphogenesis | 4.5 | 2.8E-03 |
| GO:0001525~angiogenesis | 4.8 | 3.3E-03 |
| GO:0048646~anatomical structure formation | 4.2 | 4.9E-03 |
| GO:0001568~blood vessel development | 4.0 | 7.6E-03 |
| GO:0001944~vasculature development | 3.9 | 8.8E-03 |
| GO:0009887~organ morphogenesis | 2.7 | 1.8E-02 |

The significantly upregulated GO terms in adipose tissue (n=7) identified by DAVID. The GO terms were categorized into broader GO categories such as: inflammation, development, signaling, metal ion homeostasis, secretion, and angiogenesis.

Additional file 2, Table S2

**GO analysis for the downregulated adipose tissue transcriptome**

| **extracellular matrix** | Fold Enrichment | Benjamini |
| --- | --- | --- |
| GO:0005576~extracellular region | 2.5 | 1.3E-06 |
| GO:0009605~response to external stimulus | 2.6 | 1.5E-02 |

The significantly downregulated GO terms in adipose tissue (n=7) identified by DAVID. The GO terms belonged to GO category extracellular matrix.

Additional file 2, Table S3

**GO analysis for the upregulated liver tissue transcriptome**

| **inflammation** | Fold Enrichment | Benjamini |
| --- | --- | --- |
| GO:0002376~immune system process | 4.5 | 8.1E-14 |
| GO:0006955~immune response | 5.1 | 1.2E-12 |
| GO:0008009~chemokine activity | 26.2 | 9.3E-12 |
| GO:0042379~chemokine receptor binding | 25.6 | 6.2E-12 |
| GO:0006954~inflammatory response | 7.5 | 1.1E-11 |
| GO:0042330~taxis | 11.3 | 6.9E-11 |
| GO:0006935~chemotaxis | 11.3 | 6.9E-11 |
| GO:0005125~cytokine activity | 8.5 | 1.1E-10 |
| GO:0006952~defense response | 5.0 | 1.4E-10 |
| GO:0001664~G-protein-coupled receptor binding | 17.3 | 7.4E-10 |
| GO:0009611~response to wounding | 5.5 | 1.8E-09 |
| GO:0007626~locomotory behavior | 8.5 | 4.7E-09 |
| GO:0009605~response to external stimulus | 4.1 | 8.2E-08 |
| GO:0044421~extracellular region part | 3.5 | 6.7E-07 |
| GO:0005615~extracellular space | 4.2 | 7.9E-07 |
| GO:0005102~receptor binding | 3.6 | 2.8E-06 |
| GO:0005576~extracellular region | 2.8 | 2.7E-06 |
| GO:0050896~response to stimulus | 2.1 | 4.9E-06 |
| GO:0007610~behavior | 5.1 | 1.5E-05 |
| GO:0042221~response to chemical stimulus | 3.8 | 1.6E-05 |
| GO:0006950~response to stress | 2.6 | 8.0E-04 |
| GO:0007267~cell-cell signaling | 2.9 | 6.6E-03 |
| GO:0051674~localization of cell | 3.2 | 5.1E-02 |
| GO:0006928~cell motility | 3.2 | 5.1E-02 |
| **development** | Fold Enrichment | Benjamini |
| GO:0016265~death | 2.6 | 6.8E-03 |
| GO:0008219~cell death | 2.6 | 6.8E-03 |
| GO:0012501~programmed cell death | 2.6 | 8.4E-03 |
| GO:0048468~cell development | 2.2 | 1.3E-02 |
| GO:0006915~apoptosis | 2.6 | 1.9E-02 |
| GO:0030154~cell differentiation | 1.9 | 2.4E-02 |
| GO:0048869~cellular developmental process | 1.9 | 2.4E-02 |
| GO:0042127~regulation of cell proliferation | 4.0 | 2.4E-05 |
| GO:0008283~cell proliferation | 2.8 | 1.3E-03 |
| GO:0008284~positive regulation of cell proliferation | 4.3 | 2.3E-02 |
| GO:0008285~negative regulation of cell proliferation | 4.3 | 2.3E-02 |
| **angiogenesis** | Fold Enrichment | Benjamini |
| GO:0001525~angiogenesis | 5.4 | 4.8E-02 |

The significantly upregulated GO terms in liver tissue (n=5) identified by DAVID. The GO terms were categorized into broader GO categories such as: inflammation, development, and angiogenesis.

Additional file 2, Table S4

**GO analysis for the downregulated liver tissue transcriptome**

| **amino acid metabolism** | Fold Enrichment | Benjamini |
| --- | --- | --- |
| GO:0009308~amine metabolic process | 4.9 | 3.3E-05 |
| GO:0006519~amino acid and derivative metabolic process | 5.5 | 2.4E-05 |
| GO:0006520~amino acid metabolic process | 6.0 | 3.3E-05 |
| GO:0006807~nitrogen compound metabolic process | 4.6 | 2.8E-05 |
| GO:0009063~amino acid catabolic process | 15.5 | 9.9E-05 |
| GO:0019752~carboxylic acid metabolic process | 4.0 | 1.9E-04 |
| GO:0006082~organic acid metabolic process | 4.0 | 1.8E-04 |
| GO:0009310~amine catabolic process | 13.7 | 1.7E-04 |
| GO:0044270~nitrogen compound catabolic process | 13.3 | 1.9E-04 |
| **inflammation/binding** | Fold Enrichment | Benjamini |
| GO:0030246~carbohydrate binding | 6.1 | 4.2E-05 |
| GO:0005539~glycosaminoglycan binding | 9.4 | 6.7E-03 |
| GO:0030247~polysaccharide binding | 9.1 | 4.5E-03 |
| GO:0001871~pattern binding | 8.2 | 7.4E-03 |
| GO:0048037~cofactor binding | 5.9 | 5.1E-03 |

The significantly downregulated GO terms in liver tissue (n=5) identified by DAVID. The GO terms belonged to GO categories: amino acid metabolism and inflammation/binding.
