# Supplementary material for: Comparative analysis of the human hepatic and adipose tissue transcriptomes during LPS-induced inflammation leads to the identification of differential biological pathways and candidate biomarkers
Source: BMC Med Genomics. 2011 Oct 6;4:71. doi: 10.1186/1755-8794-4-71 (PMC3196688; doi:10.1186/1755-8794-4-71)
Supplement: Additional file 3 — Gene count analysis for the identified GO categories. Table S1. Gene count analysis for the identified GO categories in the significant adipose tissue transcriptome. The significantly upregulated (up) and downregulated (down) genes in adipose tissue (AT), (n = 7) within the defined GO categories (inflammation, development, signaling, metal ion homeostasis, secretion, angiogenesis, and extracellular region. Table S2. Gene count analysis for the identified GO categories in the significant liver tissue transcriptome. The significantly upregulated (up) and downregulated (down) genes in liver tissue (LT), (n = 5) within the defined GO categories (inflammation, development, signaling, angiogenesis, amino acid metabolism, and inflammation/binding). [file 1755-8794-4-71-S3.DOC]

### Additional file 3 – Gene count analysis for the identified GO categories

###

Additional file 3, Table S1

**Gene count analysis for the identified GO categories in the significant adipose tissue transcriptome**

| inflammation AT up | development AT up | signaling AT up | metal ion homeostasis AT up | secreion AT up | angiogenesis AT up | extracellular region AT down |
| --- | --- | --- | --- | --- | --- | --- |
| CXCL2 | ABL1 | CD55 | NRP2 | SAA1 | NRP2 | SFRP2 |
| IL1B | ADORA2A | SERPINA1 | POU3F1 | OSM | IL1B | RNASET2 |
| IL11 | ANGPTL4 | CASP4 | GNA15 | IL11 | TGM2 | DKK1 |
| SERPINA1 | ANPEP | PTX3 | ATP2A2 | INHBA | ANPEP | SLIT3 |
| IL23A | AQP9 | DIO2 | SOCS3 | NGFB | TGFA | FLJ23834 |
| EREG | ARID5A | ARID5A | IL1B | NOD2 | TNFAIP2 | RNASE1 |
| CSF3 | ATP2A2 | AQP9 | NRG1 | SRGN | EREG | C20ORF103 |
| C8A | ATXN7 | MSX2 | IL11 | LIF | EDN1 | CD302 |
| CXCL5 | BCL2A1 | ATF3 | CD55 | NLRP3 | CYR61 | IGFL2 |
| CXCL6 | BMP2 | SOX11 | SERPINA1 | EDN1 | SERPINE1 | THSD1 |
| CCL8 | C8A | CASP5 | WISP1 | SYN1 | IL8 | DKFZP586H2123 |
| IL19 | CA2 | FOXD1 | PLAUR | TBX3 | FGF2 | LGMN |
| CCL3 | CASP4 | SERPINB2 | SOCS1 | SOCS1 | ANGPTL4 | PDGFD |
| LTB | CASP5 | ELL2 | SGMS1 |  | NR4A3 | FBLN1 |
| IL1RN | CCL2 | IRF6 | SERPINE1 |  | CCL2 | WNT4 |
| EDN1 | CCL4L2 | SERPINB4 | OSM |  | NDP | MMP28 |
| EBI3 | CD274 | RHCG | MCL1 |  | BMP2 | CD109 |
| CX3CL1 | CD38 | CYP27B1 | CCL5 |  | TBX3 | FOLR2 |
| CSF2 | CD44 | BCL2A1 | RHCG |  | LIF | EMCN |
| HBEGF | CD55 | ZNF281 | ITGA2 |  | ITGA2 | EMID1 |
| CCL20 | CDKN1A | SRGN | CYP27B1 |  | MCL1 | NOV |
| OSM | CHGA | RFX3 | EDG2 |  | ZNF281 | HTRA1 |
| S100A9 | CHST2 | SGMS1 | CCL2 |  | ZIC2 | DNASE2 |
| FGF2 | CLCF1 | NRCAM | SLC22A4 |  | FCRLA | TCN2 |
| CCL5 | CLCN1 | C8A | CDKN1A |  | WNT2 | EPHA3 |
| CXCL11 | CLDN14 | SLC22A4 | WNT2 |  | PRRX1 | PTPRF |
| LIF | CRADD | TGIF1 | IL1A |  | WNT5A | METTL7A |
| CCL4L2 | CSF2 | CYR61 | TGM2 |  | MSX2 | EFEMP1 |
| ORM1 | CSF3 | SAA1 | MAPKAPK2 |  |  | WISP2 |
| CCL2 | CX3CL1 | CHGA | CCL3 |  |  | LOXL4 |
| NDP | CXCL1 | MAFF | AQP9 |  |  | COL4A5 |
| IL1A | CXCL10 | PTGS2 | EDN1 |  |  | COL8A1 |
| IL10 | CXCL5 | TNIP1 | CCR7 |  |  | PLXDC2 |
| IL24 | CYR61 | PRRX1 | CYR61 |  |  | ACP5 |
| BMP2 | DNER | SERPINE1 | SAA1 |  |  | METTL7B |
| TNFSF15 | EBI3 | SP6 | INHBA |  |  | CA11 |
| CXCL1 | EDG2 | PLK3 | CHGA |  |  | PCDH7 |
| S100A8 | EDN1 | PRDM8 | TNFRSF1B |  |  | CYYR1 |
| IL8 | EREG | POU2F2 | PTGS2 |  |  | LIPA |
| INHBA | FCRLA | IER3 | ADORA2A |  |  | CPVL |
| CXCL10 | FGF2 | MTF1 | IL2RA |  |  | C1QTNF5 |
| WNT2 | GCH1 | CXCL2 | GCH1 |  |  | CXORF36 |
| ANGPTL4 | GZMB | GZMB | P2RY6 |  |  | LRRN2 |
| MMP12 | HBEGF | PITPNC1 | DGKG |  |  | TINAGL1 |
| PTX3 | HCK | LTB | CA8 |  |  | GHR |
| CYR61 | HSD11B1 | CA8 | IL8 |  |  | KAZALD1 |
| SAA1 | IER3 | MPZL1 |  |  |  | FUCA1 |
| TNFAIP6 | IL10 | CXCL11 |  |  |  | CD248 |
| SERPINE1 | IL11 | KREMEN1 |  |  |  | FGL2 |
| SERPINB4 | IL15RA | NDP |  |  |  | BCHE |
| HPSE | IL19 | PDE4B |  |  |  | SEMA5A |
| LILRA3 | IL1A | CXCL6 |  |  |  | CLEC14A |
| PLAUR | IL1B | PTGES |  |  |  | ADAMTSL3 |
| IL7R | IL23A | TNFAIP6 |  |  |  | DNASE1L1 |
| TNFRSF1B | IL24 | CCL20 |  |  |  | ASTN2 |
| CD55 | IL2RA | TNC |  |  |  | C8ORF55 |
| NOD2 | IL7R | HCK |  |  |  | GAS6 |
| INDO | IL8 | SLC1A2 |  |  |  | PCDH18 |
| GBP5 | INHBA | PDE4D |  |  |  | PCDH17 |
| SLAMF7 | ITGA2 | ANPEP |  |  |  | OLFML3 |
| POU2F2 | ITGB8 | FCRLA |  |  |  | IL11RA |
| AQP9 | LAMB3 | ZP3 |  |  |  | CPA4 |
| GBP1 | LAMC2 | IL1RN |  |  |  | CDON |
| PDCD1LG2 | LCE3D | PDCD1LG2 |  |  |  | EPHB6 |
| PAG1 | LIF | CD44 |  |  |  | ISLR |
| CD274 | MAFF | FCAMR |  |  |  | SULF2 |
| IL2RA | MAP2K3 | WNT2 |  |  |  | OLFML1 |
| CDKN1A | MCL1 | NLRP3 |  |  |  | PDGFRL |
| RIPK2 | MMP1 | CCR7 |  |  |  | LRRC17 |
| CCR7 | MMP3 | IRAK2 |  |  |  | MFNG |
| DNER | MMP8 | NR3C1 |  |  |  | FCN3 |
| ADORA2A | MSX2 | TRAF3IP2 |  |  |  | DLC1 |
| NRCAM | NDP | IL2RA |  |  |  | SGCD |
| CD48 | NGFB | PLAUR |  |  |  | MEST |
| PTGS2 | NLRP3 | RGS16 |  |  |  | ST3GAL5 |
| NRP2 | NOD2 | IL7R |  |  |  | GPR1 |
| ITGA2 | NR3C1 | CD38 |  |  |  | KCNA4 |
| SELE | NR4A3 | CCL2 |  |  |  | KCNA5 |
| OR4Q3 | NRCAM | IL1A |  |  |  | PLVAP |
| ABL1 | NRG1 | RIPK2 |  |  |  | GLT8D2 |
| CHST2 | NRP2 | PAG1 |  |  |  | SCARB1 |
| TGFA | OR4Q3 | TNFRSF1B |  |  |  | AQP1 |
| SRGN | OSM | ADORA2A |  |  |  | ANXA4 |
| IL33 | OSMR | CD274 |  |  |  | GPR146 |
| TNFAIP2 | PAG1 | GNA15 |  |  |  | MSR1 |
| MMP8 | PBEF1 | IL1B |  |  |  | MAN1C1 |
| CHGA | PDE4D | TGFA |  |  |  | PPAP2A |
| WNT5A | PHLDA1 | RGS3 |  |  |  | GPER |
| MMP1 | PIM2 | CCL3 |  |  |  | TSPAN7 |
| CLCF1 | PLAUR | HBEGF |  |  |  | MRGPRF |
| MMP3 | POU2F2 | CCL5 |  |  |  | GPM6A |
| NRG1 | POU3F1 | ITGA2 |  |  |  | GAL3ST4 |
| PBEF1 | PRRX1 | EDG2 |  |  |  | GALNTL2 |
| ZP3 | PTGS2 | MAPKAPK2 |  |  |  | GPR81 |
| LAMC2 | PTX3 | TRAF1 |  |  |  | GPR34 |
| TNC | RAC2 | INHBA |  |  |  | SLCO2B1 |
| LAMB3 | RFX3 | NGFB |  |  |  | FAIM2 |
| WISP1 | RGS16 | P2RY6 |  |  |  | COX4I2 |
| IL15RA | RGS3 | OR4Q3 |  |  |  | PTP4A3 |
| MUCL1 | RHCG | SRC |  |  |  | TFF3 |
| FCRLA | RIPK2 | ICAM1 |  |  |  | HLA-DMA |
| METRNL | RTTN | MRGPRX3 |  |  |  | HLA-DMB |
| SERPINB2 | SAA1 | IL15RA |  |  |  | STAB1 |
| TNFRSF6B | SERPINA1 | CCL8 |  |  |  | MMRN1 |
| NGFB | SERPINB2 | PILRA |  |  |  | FBN1 |
| PLA1A | SERPINE1 | SLAMF7 |  |  |  | CCL21 |
| LIPG | SGMS1 | PTPN2 |  |  |  | HAMP |
| SLAMF1 | SLAMF1 | EBI3 |  |  |  | CA4 |
| OSMR | SLAMF7 | SLAMF1 |  |  |  | FBLN5 |
| SOCS1 | SLC1A2 | NR4A3 |  |  |  | ANG |
| POU3F1 | SLC22A4 | NRP2 |  |  |  | A2M |
| SYN1 | SOCS1 | OSMR |  |  |  | LYZ |
| SLC1A2 | SOCS3 | LILRA3 |  |  |  | LYVE1 |
| TBX3 | SOX11 | GPR64 |  |  |  | CTSB |
| MPZL1 | SRGN | WNT5A |  |  |  | TLR7 |
| GCH1 | SYN1 | S100A9 |  |  |  | TREM2 |
| TDO2 | TBX3 | CCL4L2 |  |  |  | INHBB |
| GZMB | TDO2 | GPR84 |  |  |  | GPX3 |
| ICAM1 | TGFA | TNFRSF6B |  |  |  | FCN1 |
| LRIG1 | TGIF1 | ITGB8 |  |  |  | C1QA |
| DKFZP564O0823 | TGM2 | DNER |  |  |  | F8 |
| CD44 | TMEM166 |  |  |  |  | LY86 |
| GPR64 | TNC |  |  |  |  | IFI30 |
| KREMEN1 | TNFAIP2 |  |  |  |  | CCL15 |
| LRRN3 | TNFRSF1B |  |  |  |  | RELN |
| IL4I1 | TNFRSF6B |  |  |  |  | COL1A1 |
| ITGB8 | TNFSF15 |  |  |  |  | SEPP1 |
| P2RY6 | TRAF1 |  |  |  |  | GM2A |
| SLC7A5 | TRAF3IP2 |  |  |  |  | LIPE |
| HSD11B1 | WISP1 |  |  |  |  | TNFSF13B |
| C19ORF59 | WNT2 |  |  |  |  | F13A1 |
| SLC2A6 | WNT5A |  |  |  |  | GNGT2 |
| GPR84 | ZIC2 |  |  |  |  | FPRL2 |
| CD38 | ZNF281 |  |  |  |  | CD36 |
| CD82 |  |  |  |  |  | CCBP2 |
| SLCO4A1 |  |  |  |  |  | ACCN2 |
| SLC7A2 |  |  |  |  |  | ADRA1B |
| ANPEP |  |  |  |  |  | SCARA3 |
| ENTPD7 |  |  |  |  |  | BLNK |
| SLC7A11 |  |  |  |  |  | TBXAS1 |
| C6ORF128 |  |  |  |  |  | PPARG |
| EDG2 |  |  |  |  |  | EPHX2 |
| IER3 |  |  |  |  |  | PDE6B |
| TGM2 |  |  |  |  |  | AOX1 |
| NLRP3 |  |  |  |  |  | AIF1 |
| IRAK2 |  |  |  |  |  | FABP4 |
| NR3C1 |  |  |  |  |  | TLR5 |
| HIST1H2BJ |  |  |  |  |  | GSTA4 |
| DNAJC3 |  |  |  |  |  | PHF17 |
| HIST1H2BK |  |  |  |  |  | GNG7 |
| TNIP1 |  |  |  |  |  | NDRG4 |
| RAC2 |  |  |  |  |  | FANCM |
| MAFF |  |  |  |  |  | KCNJ8 |
| DNAJB5 |  |  |  |  |  | MNDA |
| INSIG2 |  |  |  |  |  | ARHGDIB |
| ACSL4 |  |  |  |  |  | ZNF179 |
| MTF1 |  |  |  |  |  | LST1 |
| PLA2G4A |  |  |  |  |  | RNF125 |
| MAP2K3 |  |  |  |  |  | NOS3 |
| ATP2A2 |  |  |  |  |  | MAOA |
| CLDN14 |  |  |  |  |  |  |
| ZIC2 |  |  |  |  |  |  |

The significantly upregulated (up) and downregulated (down) genes in adipose tissue (AT), (n=7) within the defined GO categories (inflammation, development, signaling, metal ion homeostasis, secretion, angiogenesis, and extracellular region).

Additional file 3, Table S2

**Gene count analysis for the identified GO categories in the significant liver tissue transcriptome**

| inflammation LT up | development LT up | signaling LT up | angiogenesis LT up | amino acid metabolism LT down | inflammation/binding LT down |
| --- | --- | --- | --- | --- | --- |
| ADA | TNFAIP3 | AQP9 | NRP2 | PSAT1 | LYVE1 |
| CCL7 | IL1B | SERPINB2 | PML | AGMAT | CLEC4M |
| IL1B | PIM1 | SERPINB4 | IL8 | LYVE1 | FCN2 |
| IL32 | SERPINB2 | BCL2A1 | SPHK1 | GAMT | MRC1L1 |
| TAP1 | TNFAIP8 | NRCAM | IL1B | ACY1 | KNG1 |
| CCL20 | NFKB1 | PTGS2 | FGF2 | PRODH2 | SERPIND1 |
| BPGM | BCL2L11 | TNIP1 | TNFAIP2 | SHMT1 | CCL15 |
| IFITM1 | KRAS | PRRX1 | JAG1 | SLC7A9 | HABP2 |
| PSMB9 | B4GALT1 | GZMB | B4GALT1 | THNSL1 | HRG |
| B4GALT1 | TLR2 | MPZL1 | CCL2 | GCAT | GPNMB |
| TLR2 | CD40 | CXCL11 | CSF1 | FTCD | MRC1 |
| CD40 | STAT1 | CXCL6 | KRAS | HIBCH | FCN3 |
| CD47 | SPHK1 | TNFAIP6 | NRCAM | MAT1A | STAB1 |
| IL7R | FGF2 | CCL20 | ABTB2 | HAL | MBL2 |
| SERPINB4 | CCL2 | TNC | EHF | ITIH2 | POSTN |
| GBP4 | BCL2A1 | ZP3 | DFNA5 | CPS1 | CLEC4G |
| CCL5 | IL1A | IL1RN | CD44 | DDAH1 | ASGR1 |
| LYN | PML | CD44 |  | ATP2B2 | CLDN7 |
| TAPBP | JAK2 | IRAK2 |  | GOT1 | F8 |
| GBP5 | BID | TRAF3IP2 |  | PAH | RELN |
| CTSS | TNFRSF1B | IL7R |  | GCSH | ANGPTL3 |
| CXCL11 | NRP2 | CCL2 |  | ACOX2 | CDH1 |
| CXCL5 | NRCAM | IL1A |  | SLC27A5 | ICAM2 |
| CXCL3 | RND1 | TNFRSF1B |  | PFKFB1 | PSAT1 |
| CCL2 | JAG1 | IL1B |  | TPD52L1 | ACOX2 |
| PSMB10 | EHF | CCL3 |  | DNASE1L3 | RETSAT |
| CXCL6 | LYN | CCL5 |  | GBA3 | SHMT1 |
| IL1A | CSF1 | ICAM1 |  | HSD17B6 | GOT1 |
| CCL3 | PTGS2 | EBI3 |  | ANGPTL3 | THNSL1 |
| HLA-F | TNIP1 | NRP2 |  | CES2 | GCAT |
| CXCL9 | CYLD | S100A9 |  |  | GCDH |
| AQP9 | BTG3 | ITGB8 |  |  | ALB |
| IL1RN | IFITM1 |  |  |  | PAH |
| JAK2 | FSTL3 |  |  |  | GCSH |
| CSF1 | RARRES1 |  |  |  | PNPO |
| CCL3L3 | AVIL |  |  |  | FTCD |
| EBI3 | CCL3L3 |  |  |  | RBP1 |
| CXCL1 | CXCL1 |  |  |  |  |
| IL8 | IL8 |  |  |  |  |
| RNF19B | MXD1 |  |  |  |  |
| TAP2 | ABTB2 |  |  |  |  |
| CXCL10 | PRRX1 |  |  |  |  |
| GZMA | IL7R |  |  |  |  |
| JAG1 | CXCL10 |  |  |  |  |
| TNFAIP6 | SYN1 |  |  |  |  |
| NFKB1 | AQP9 |  |  |  |  |
| S100A9 | EBI3 |  |  |  |  |
| IRAK2 | SNCAIP |  |  |  |  |
| TNFRSF1B | TFPI2 |  |  |  |  |
| PTGS2 | CD47 |  |  |  |  |
| NRP2 | GAD1 |  |  |  |  |
| KRAS | DRAM |  |  |  |  |
| FGF2 | GZMB |  |  |  |  |
| JAK1 | OPTN |  |  |  |  |
| TNIP1 | GZMA |  |  |  |  |
| NLRC5 | BPGM |  |  |  |  |
| TFPI2 | TNC |  |  |  |  |
| PI3 | TUBD1 |  |  |  |  |
| MMP7 | TNFAIP2 |  |  |  |  |
| TNC | DFNA5 |  |  |  |  |
| LAMB3 | NAV1 |  |  |  |  |
| CTHRC1 | STS |  |  |  |  |
| FSTL3 | HSD11B1 |  |  |  |  |
| ZP3 | CD44 |  |  |  |  |
| TNFAIP2 | SMTN |  |  |  |  |
| MMP3 | LAMB3 |  |  |  |  |
| MMP12 | DUOX2 |  |  |  |  |
| BID | ITGB8 |  |  |  |  |
| GABBR1 | PHC1 |  |  |  |  |
| SERPINB2 | CYP1B1 |  |  |  |  |
| SLPI | MMP7 |  |  |  |  |
| MUCL1 | LOC441453 |  |  |  |  |
| LCN2 | MMP3 |  |  |  |  |
| C12orf39 | CXCL5 |  |  |  |  |
| BTN3A3 | CDC25B |  |  |  |  |
| GZMB | TRAF3IP2 |  |  |  |  |
| PSMA6 | GABBR1 |  |  |  |  |
| ICAM1 | ICAM1 |  |  |  |  |
| TPBG | NCOA7 |  |  |  |  |
| STAT1 | TRIB2 |  |  |  |  |
| STS | CCL3 |  |  |  |  |
| ADAM17 | NHEDC2 |  |  |  |  |
| MPZL1 | IRAK2 |  |  |  |  |
| CD44 | CCL7 |  |  |  |  |
| LAP3 | SERPINB4 |  |  |  |  |
| PDZK1IP1 | CCL5 |  |  |  |  |
| CYP1B1 | SP100 |  |  |  |  |
| PML | WTAP |  |  |  |  |
| DUOX2 |  |  |  |  |  |
| LOC441453 |  |  |  |  |  |
| AVIL |  |  |  |  |  |
| MAP3K8 |  |  |  |  |  |
| SYN1 |  |  |  |  |  |
| SNCAIP |  |  |  |  |  |
| GAD1 |  |  |  |  |  |
| MARCKS |  |  |  |  |  |
| SPHK1 |  |  |  |  |  |
| NRCAM |  |  |  |  |  |

The significantly upregulated (up) and downregulated (down) genes in liver tissue (LT), (n=5) within the defined GO categories (inflammation, development, signaling, angiogenesis, amino acid metabolism, and inflammation/binding).
