# Supplementary material for: Comparative analysis of the human hepatic and adipose tissue transcriptomes during LPS-induced inflammation leads to the identification of differential biological pathways and candidate biomarkers
Source: BMC Med Genomics. 2011 Oct 6;4:71. doi: 10.1186/1755-8794-4-71 (PMC3196688; doi:10.1186/1755-8794-4-71)
Supplement: Additional file 4 — Secretome prediction. Table S1. The common (overlapping) adipose tissue and liver tissue predicted secretome. The common (overlapping) adipose tissue (AT), (n = 7) and liver tissue (LT), (n = 5) predicted secretome. The presented genes were significantly changed, p ≤ 0.05 in both tissues. In the last two columns fold changes (FC) in AT and LT are given. Table S2. The adipose tissue predicted secretome. The adipose tissue (AT) predicted secretome, (n = 7). Genes present in AT were significantly changed (p ≤ 0.05, FC > 2) while the corresponding genes in liver tissue (LT), (n = 5) were not significantly affected (p > 0.05). Table S3. The liver tissue predicted secretome. The liver tissue (LT) predicted secretome, n = 5. Genes present in LT were significantly changed (p ≤ 0.05, FC > 2) while the corresponding genes in adipose tissue (AT), (n = 7) were not significantly affected (p > 0.05). [file 1755-8794-4-71-S4.DOC]

### Additional file 4 – Secretome prediction

Additional file 4, Table S1

The common (overlapping) adipose tissue and liver tissue predicted secretome.

| ACCESSION | NAME | SYMBOL | FC AT | FC LT |
| --- | --- | --- | --- | --- |
| P01584 | INTERLEUKIN 1, BETA | IL1B | 20 | 100 |
| P10147 | CHEMOKINE (C-C MOTIF) LIGAND 3 | CCL3 | 19.8 | 10.7 |
| Q96DR8 | SMALL BREAST EPITHELIAL MUCIN | MUCL1 | 17.4 | 5.8 |
| Q9C002 | CHROMOSOME 15 OPEN READING FRAME 48 | C15ORF48 | 13.9 | 5.6 |
| P18510 | INTERLEUKIN 1 RECEPTOR ANTAGONIST | IL1RN | 12.5 | 6.5 |
| P78556 | CHEMOKINE (C-C MOTIF) LIGAND 20 | CCL20 | 11.4 | 7.5 |
| Q8WWV6 | FC RECEPTOR, IGA, IGM, HIGH AFFINITY | FCAMR | 10.3 | 13.8 |
| Q92823 | NEURONAL CELL ADHESION MOLECULE | NRCAM | 9.4 | 3.3 |
| P26012 | INTEGRIN, BETA 8 | ITGB8 | 8.6 | 3.7 |
| P42830 | CHEMOKINE (C-X-C MOTIF) LIGAND 5 | CXCL5 | 7.8 | 30.3 |
| Q86SG4 | CHROMOSOME 15 OPEN READING FRAME 21 | C15ORF21 | 7 | 3.8 |
| P35354 | PROSTAGLANDIN-ENDOPEROXIDE SYNTHASE 2 (PROSTAGLANDIN G/H SYNTHASE AND CYCLOOXYGENASE) | PTGS2 | 6.8 | 7.9 |
| P13501 | CHEMOKINE (C-C MOTIF) LIGAND 5/RANTES | CCL5 | 6.3 | 68 |
| Q14213 | EPSTEIN-BARR VIRUS INDUCED GENE 3 | EBI3 | 6.3 | 2.6 |
| P05120 | SERPIN PEPTIDASE INHIBITOR, CLADE B (OVALBUMIN), MEMBER 2 | SERPINB2 | 6 | 8 |
| P01583 | INTERLEUKIN 1, ALPHA | IL1A | 5.8 | 4.3 |
| P16871 | INTERLEUKIN 7 RECEPTOR | IL7R | 5.8 | 5.2 |
| O14625 | CHEMOKINE (C-X-C MOTIF) LIGAND 11 | CXCL11 | 5.4 | 20.4 |
| Q8N9Z2 | HYPOTHETICAL PROTEIN FLJ36031 | FLJ36031 | 4.8 | 2.1 |
| P05231 | INTERLEUKIN 6 (INTERFERON, BETA 2) | IL6 | 4.5 | 17.8 |
| P08254 | MATRIX METALLOPEPTIDASE 3 (STROMELYSIN 1, PROGELATINASE) | MMP3 | 4 | 20.6 |
| P09038 | FIBROBLAST GROWTH FACTOR 2 (BASIC) | FGF2 | 3.7 | 3.5 |
| P39900 | MATRIX METALLOPEPTIDASE 12 (MACROPHAGE ELASTASE) | MMP12 | 3.7 | 5.8 |
| O43315 | AQUAPORIN 9 | AQP9 | 3.4 | 2.9 |
| Q15077 | PYRIMIDINERGIC RECEPTOR P2Y, G-PROTEIN COUPLED, 6 | P2RY6 | 3.4 | 5.6 |
| P15144 | ALANYL (MEMBRANE) AMINOPEPTIDASE (AMINOPEPTIDASE N, AMINOPEPTIDASE M, MICROSOMAL AMINOPEPTIDASE, CD13, P150) | ANPEP | 3.2 | -3.6 |
| P53671 | LIM DOMAIN KINASE 2 | LIMK2 | 3.2 | 3.1 |
| P98066 | TUMOR NECROSIS FACTOR, ALPHA-INDUCED PROTEIN 6 | TNFAIP6 | 3.1 | 18 |
| P21754 | ZONA PELLUCIDA GLYCOPROTEIN 3 (SPERM RECEPTOR) | ZP3 | 3.1 | 3.8 |
| P09341 | CHEMOKINE (C-X-C MOTIF) LIGAND 1 (MELANOMA GROWTH STIMULATING ACTIVITY, ALPHA) | CXCL1 | 3 | 23.5 |
| P02778 | CHEMOKINE (C-X-C MOTIF) LIGAND 10 | CXCL10 | 2.9 | 17 |
| Q13751 | LAMININ, BETA 3 | LAMB3 | 2.9 | 10.4 |
| O95297 | MYELIN PROTEIN ZERO-LIKE 1 | MPZL1 | 2.9 | 2.5 |
| P54821 | PAIRED RELATED HOMEOBOX 1 | PRRX1 | 2.9 | 3.9 |
| P24821 | TENASCIN C (HEXABRACHION) | TNC | 2.9 | 2.9 |
| P16070 | CD44 ANTIGEN (INDIAN BLOOD GROUP) | CD44 | 2.8 | 3.5 |
| P80162 | CHEMOKINE (C-X-C MOTIF) LIGAND 6 (GRANULOCYTE CHEMOTACTIC PROTEIN 2) | CXCL6 | 2.8 | 22 |
| P10144 | GRANZYME B (GRANZYME 2, CYTOTOXIC T-LYMPHOCYTE-ASSOCIATED SERINE ESTERASE 1) | GZMB | 2.8 | 6.1 |
| O60462 | NEUROPILIN 2 | NRP2 | 2.6 | 2.6 |
| P10145 | INTERLEUKIN 8 | IL8 | 2.5 | 6 |
| P28845 | HYDROXYSTEROID (11-BETA) DEHYDROGENASE 1 | HSD11B1 | 2.4 | 2.5 |
| P05362 | INTERCELLULAR ADHESION MOLECULE 1 (CD54), HUMAN RHINOVIRUS RECEPTOR | ICAM1 | 2.4 | 4.8 |
| P20333 | TUMOR NECROSIS FACTOR RECEPTOR SUPERFAMILY, MEMBER 1B | TNFRSF1B | 2.4 | 2.5 |
| P13500 | CHEMOKINE (C-C MOTIF) LIGAND 2 | CCL2 | 2.3 | 3.7 |
| P17706 | PROTEIN TYROSINE PHOSPHATASE, NON-RECEPTOR TYPE 2 | PTPN2 | 2.3 | 2.1 |
| O14684 | PROSTAGLANDIN E SYNTHASE | PTGES | 2.2 | 4.2 |
| Q99424 | ACYL-COENZYME A OXIDASE 2, BRANCHED CHAIN | ACOX2 | -2.1 | -13.1 |
| Q9NR77 | PEROXISOMAL MEMBRANE PROTEIN 2, 22KDA | PXMP2 | -2.1 | -3.2 |
| Q92506 | HYDROXYSTEROID (17-BETA) DEHYDROGENASE 8 | HSD17B8 | -2.2 | -2.5 |
| O00587 | MANIC FRINGE HOMOLOG (DROSOPHILA) | MFNG | -2.2 | -2.1 |
| Q9NY15 | STABILIN 1 | STAB1 | -2.2 | -3 |
| P15090 | FATTY ACID BINDING PROTEIN 4, ADIPOCYTE | FABP4 | -2.4 | -7 |
| Q14353 | GUANIDINOACETATE N-METHYLTRANSFERASE | GAMT | -2.4 | -4 |
| O43679 | LIM DOMAIN BINDING 2 | LDB2 | -2.4 | -3.4 |
| Q00013 | MEMBRANE PROTEIN, PALMITOYLATED 1, 55KDA | MPP1 | -2.4 | -2.6 |
| P00451 | COAGULATION FACTOR VIII, PROCOAGULANT COMPONENT (HEMOPHILIA A) | F8 | -2.5 | -2.6 |
| P78509 | REELIN | RELN | -2.9 | -5 |
| Q16890 | TUMOR PROTEIN D52-LIKE 1 | TPD52L1 | -3.2 | -2 |
| P49908 | SELENOPROTEIN P, PLASMA, 1 | SEPP1 | -3.8 | -3.2 |
| Q6UX53 | METHYLTRANSFERASE LIKE 7B | METTL7B | -5.3 | -7.3 |
| P13686 | ACID PHOSPHATASE 5, TARTRATE RESISTANT | ACP5 | -5.6 | -6.1 |
| Q5EB52 | MESODERM SPECIFIC TRANSCRIPT HOMOLOG (MOUSE) | MEST | -6.2 | -2.6 |
| P07998 | RIBONUCLEASE, RNASE A FAMILY, 1 (PANCREATIC) | RNASE1 | -6.2 | -4.6 |
| Q9Y5Y7 | EXTRACELLULAR LINK DOMAIN CONTAINING 1 | LYVE1 | -7.3 | -7.9 |
| P06276 | BUTYRYLCHOLINESTERASE | BCHE | -7.7 | -6.1 |
| Q16663 | CHEMOKINE (C-C MOTIF) LIGAND 15 | CCL15 | -10.2 | -10.6 |

The common (overlapping) adipose tissue (AT), (n=7) and liver tissue (LT), (n=5) predicted secretome. The presented genes were significantly changed, p≤0.05 in both tissues. In the last two columns fold changes (FC) in AT and LT are given.

Additional file 4, Table S2

**The significant adipose tissue predicted secretome.**

| ACCESSION | NAME | SYMBOL | FC AT | FC LT |
| --- | --- | --- | --- | --- |
| Q8NH05 | OLFACTORY RECEPTOR, FAMILY 4, SUBFAMILY Q, MEMBER 3 | OR4Q3 | 199.5 | probe not present |
| P16581 | SELECTIN E (ENDOTHELIAL ADHESION MOLECULE 1) | SELE | 105.1 | -2.5 |
| P04141 | COLONY STIMULATING FACTOR 2 (GRANULOCYTE-MACROPHAGE) | CSF2 | 82.5 | 1 |
| Q92629 | SARCOGLYCAN, DELTA (35KDA DYSTROPHIN-ASSOCIATED GLYCOPROTEIN) | SGCD | 58.8 | -2.5 |
| Q9BYE3 | LATE CORNIFIED ENVELOPE 3D | LCE3D | 46 | -1.4 |
| Q9NZQ7 | CD274 ANTIGEN | CD274 | 30.1 | 6.1 |
| P10645 | CHROMOGRANIN A (PARATHYROID SECRETORY PROTEIN 1) | CHGA | 27.2 | -2 |
| P02763 | OROSOMUCOID 1 | ORM1 | 26.6 | 1 |
| O14944 | EPIREGULIN | EREG | 25 | -1.2 |
| P22894 | MATRIX METALLOPEPTIDASE 8 (NEUTROPHIL COLLAGENASE) | MMP8 | 22.2 | 1.3 |
| Q13007 | INTERLEUKIN 24 | IL24 | 21.6 | 4.8 |
| Q00604 | NORRIE DISEASE (PSEUDOGLIOMA) | NDP | 19.5 | -1.1 |
| Q3SY56 | SP6 TRANSCRIPTION FACTOR | SP6 | 19.5 | 1.1 |
| P07357 | COMPLEMENT COMPONENT 8, ALPHA POLYPEPTIDE | C8A | 18.6 | 1 |
| Q8N6C8 | LEUKOCYTE IMMUNOGLOBULIN-LIKE RECEPTOR, SUBFAMILY A (WITHOUT TM DOMAIN), MEMBER 3 | LILRA3 | 17.4 | 2.2 |
| P09919 | COLONY STIMULATING FACTOR 3 (GRANULOCYTE) | CSF3 | 16.3 | -1.1 |
| Q53H76 | PHOSPHOLIPASE A1 MEMBER A | PLA1A | 14 | 2.6 |
| Q9NQS5 | G PROTEIN-COUPLED RECEPTOR 84 | GPR84 | 10.9 | 60 |
| Q8NHW4 | CHEMOKINE (C-C MOTIF) LIGAND 4 | CCL4L2 | 10.8 | probe not present |
| Q06643 | LYMPHOTOXIN BETA (TNF SUPERFAMILY, MEMBER 3) | LTB | 9.6 | -1.2 |
| Q13291 | SIGNALING LYMPHOCYTIC ACTIVATION MOLECULE FAMILY MEMBER 1 | SLAMF1 | 9.6 | 1 |
| Q7L513 | FC RECEPTOR-LIKE AND MUCIN-LIKE 1 | FCRLA | 8.7 | 2 |
| P15018 | LEUKEMIA INHIBITORY FACTOR (CHOLINERGIC DIFFERENTIATION FACTOR) | LIF | 7.1 | 2.3 |
| Q8TAE6 | PROTEIN PHOSPHATASE 1, REGULATORY (INHIBITOR) SUBUNIT 14C | PPP1R14C | 7 | 3.2 |
| Q15491 | NEUREGULIN 1 | NRG1 | 6.8 | 1.4 |
| P78423 | CHEMOKINE (C-X3-C MOTIF) LIGAND 1 | CX3CL1 | 6.4 | 14 |
| P22301 | INTERLEUKIN 10 | IL10 | 6.4 | 4.3 |
| P01589 | INTERLEUKIN 2 RECEPTOR, ALPHA | IL2RA | 6.2 | 8.1 |
| P13725 | ONCOSTATIN M | OSM | 6 | 3.9 |
| Q9UPY5 | SOLUTE CARRIER FAMILY 7, (CATIONIC AMINO ACID TRANSPORTER, Y+ SYSTEM) MEMBER 11 | SLC7A11 | 6 | 1.2 |
| P01375 | TUMOR NECROSIS FACTOR (TNF SUPERFAMILY, MEMBER 2) | TNF | 6 | 1.7 |
| Q6P656 | CHROMOSOME 15 OPEN READING FRAME 26 | C15ORF26 | 5.9 | 1 |
| P29274 | ADENOSINE A2A RECEPTOR | ADORA2A | 5.7 | 1.3 |
| Q9UHD0 | INTERLEUKIN 19 | IL19 | 5.6 | 1.2 |
| Q8IX19 | MAST CELL-EXPRESSED MEMBRANE PROTEIN 1 | C19ORF59 | 5.4 | 2.1 |
| O14896 | INTERFERON REGULATORY FACTOR 6 | IRF6 | 5.3 | -1.4 |
| O15524 | SUPPRESSOR OF CYTOKINE SIGNALING 1 | SOCS1 | 5.3 | 1.3 |
| P28907 | CD38 ANTIGEN (P45) | CD38 | 5.2 | 4.7 |
| Q92813 | DEIODINASE, IODOTHYRONINE, TYPE II | DIO2 | 5 | -1.1 |
| Q9BQ51 | PROGRAMMED CELL DEATH 1 LIGAND 2 | PDCD1LG2 | 4.9 | 1 |
| Q86VV8 | ROTATIN | RTTN | 4.7 | 1.2 |
| Q9NPF7 | INTERLEUKIN 23, ALPHA SUBUNIT P19 | IL23A | 4.6 | 1.1 |
| Q9UBD6 | RH FAMILY, C GLYCOPROTEIN | RHCG | 4.6 | 6.3 |
| P35548 | MSH HOMEOBOX HOMOLOG 2 (DROSOPHILA) | MSX2 | 4.4 | 1 |
| Q8IUE6 | HISTONE 2, H2AB | HIST2H2AB | 4.3 | probe not present |
| P20809 | INTERLEUKIN 11 | IL11 | 4.3 | 3.5 |
| P27701 | CD82 ANTIGEN | CD82 | 4.2 | 1.5 |
| P41221 | WINGLESS-TYPE MMTV INTEGRATION SITE FAMILY, MEMBER 5A | WNT5A | 4.1 | 2 |
| P04150 | NUCLEAR RECEPTOR SUBFAMILY 3, GROUP C, MEMBER 1 (GLUCOCORTICOID RECEPTOR) | NR3C1 | 4 | 1.1 |
| P26022 | PENTRAXIN-RELATED GENE, RAPIDLY INDUCED BY IL-1 BETA | PTX3 | 4 | 1.5 |
| Q9NQZ7 | ECTONUCLEOSIDE TRIPHOSPHATE DIPHOSPHOHYDROLASE 7 | ENTPD7 | 3.9 | 1 |
| O95407 | TUMOR NECROSIS FACTOR RECEPTOR SUPERFAMILY, MEMBER 6B, DECOY | TNFRSF6B | 3.9 | 1.2 |
| P32189 | GLYCEROL KINASE | GK | 3.8 | 1.4 |
| Q9UKJ1 | PAIRED IMMUNOGLOBIN-LIKE TYPE 2 RECEPTOR ALPHA | PILRA | 3.8 | 1.5 |
| Q9NQ25 | SLAM FAMILY MEMBER 7 | SLAMF7 | 3.8 | -1.1 |
| P30793 | GTP CYCLOHYDROLASE 1 (DOPA-RESPONSIVE DYSTONIA) | GCH1 | 3.7 | 2.2 |
| Q9H015 | SOLUTE CARRIER FAMILY 22 (ORGANIC CATION TRANSPORTER), MEMBER 4 | SLC22A4 | 3.7 | 1.9 |
| Q96MU8 | KRINGLE CONTAINING TRANSMEMBRANE PROTEIN 1 | KREMEN1 | 3.5 | 1 |
| P03956 | MATRIX METALLOPEPTIDASE 1 (INTERSTITIAL COLLAGENASE) | MMP1 | 3.4 | -1.1 |
| Q9P1W9 | PIM-2 ONCOGENE | PIM2 | 3.4 | 5.7 |
| P01009 | SERPIN PEPTIDASE INHIBITOR, CLADE A (ALPHA-1 ANTIPROTEINASE, ANTITRYPSIN), MEMBER 1 | SERPINA1 | 3.3 | 1 |
| Q15583 | TGFB-INDUCED FACTOR (TALE FAMILY HOMEOBOX) | TGIF1 | 3.3 | -1.2 |
| P03950 | ANGIOGENIN, RIBONUCLEASE, RNASE A FAMILY, 5 | ANG | 3.2 | -2 |
| Q9BY76 | ANGIOPOIETIN-LIKE 4 | ANGPTL4 | 3.2 | 1 |
| P46695 | IMMEDIATE EARLY RESPONSE 3 | IER3 | 3.2 | 2 |
| P09544 | WINGLESS-TYPE MMTV INTEGRATION SITE FAMILY MEMBER 2 | WNT2 | 3.2 | -1.4 |
| O95388 | WNT1 INDUCIBLE SIGNALING PATHWAY PROTEIN 1 | WISP1 | 3.2 | 1.9 |
| P35219 | CARBONIC ANHYDRASE VIII | CA8 | 3.1 | 2 |
| P80075 | CHEMOKINE (C-C MOTIF) LIGAND 8 | CCL8 | 3.1 | 6 |
| P19875 | CHEMOKINE (C-X-C MOTIF) LIGAND 2 | CXCL2 | 3.1 | 1.6 |
| Q96NY7 | CHLORIDE INTRACELLULAR CHANNEL 6 | CLIC6 | 3 | -1.1 |
| P08476 | INHIBIN, BETA A (ACTIVIN A, ACTIVIN AB ALPHA POLYPEPTIDE) | INHBA | 3 | 2.2 |
| Q9H3W5 | LEUCINE RICH REPEAT NEURONAL 3 | LRRN3 | 3 | 1.8 |
| P05121 | SERPIN PEPTIDASE INHIBITOR, CLADE E (NEXIN, PLASMINOGEN ACTIVATOR INHIBITOR TYPE 1), MEMBER 1 | SERPINE1/pai1 | 3 | 1.6 |
| Q13077 | TNF RECEPTOR-ASSOCIATED FACTOR 1 | TRAF1 | 3 | 6.2 |
| P51878 | CASPASE 5, APOPTOSIS-RELATED CYSTEINE PEPTIDASE | CASP5 | 2.9 | 2.1 |
| Q8NFT8 | DELTA-NOTCH-LIKE EGF REPEAT-CONTAINING TRANSMEMBRANE | DNER | 2.9 | 1.3 |
| P52569 | SOLUTE CARRIER FAMILY 7 (CATIONIC AMINO ACID TRANSPORTER, Y+ SYSTEM), MEMBER 2 | SLC7A2 | 2.9 | 1.1 |
| P32248 | CHEMOKINE (C-C MOTIF) RECEPTOR 7 | CCR7 | 2.8 | 3 |
| O95500 | CLAUDIN 14 | CLDN14 | 2.8 | -1.1 |
| P09326 | CD48 ANTIGEN (B-CELL MEMBRANE PROTEIN) | CD48 | 2.7 | 1.7 |
| P08174 | CD55 ANTIGEN, DECAY ACCELERATING FACTOR FOR COMPLEMENT (CROMER BLOOD GROUP) | CD55 | 2.7 | 1.2 |
| P10124 | PROTEOGLYCAN 1, SECRETORY GRANULE | SRGN | 2.7 | 1 |
| Q96BD0 | SOLUTE CARRIER ORGANIC ANION TRANSPORTER FAMILY, MEMBER 4A1 | SLCO4A1 | 2.7 | 3.1 |
| Q9UBD9 | CARDIOTROPHIN-LIKE CYTOKINE FACTOR 1 | CLCF1 | 2.6 | 1.7 |
| Q9BYT1 | CHROMOSOME 20 OPEN READING FRAME 59 | C20ORF59 | 2.6 | -1.2 |
| Q07820 | MYELOID CELL LEUKEMIA SEQUENCE 1 (BCL2-RELATED) | MCL1 | 2.6 | -1.2 |
| Q15043 | SOLUTE CARRIER FAMILY 39 (ZINC TRANSPORTER), MEMBER 14 | SLC39A14 | 2.6 | 1.1 |
| Q6UWI2 | DKFZP564O0823 PROTEIN | DKFZP564O0823 | 2.5 | -1.6 |
| P17301 | INTEGRIN, ALPHA 2 (CD49B, ALPHA 2 SUBUNIT OF VLA-2 RECEPTOR) | ITGA2 | 2.5 | -1.2 |
| P13796 | LYMPHOCYTE CYTOSOLIC PROTEIN 1 (L-PLASTIN) | LCP1 | 2.5 | 1.1 |
| Q641Q3 | METEORIN, GLIAL CELL DIFFERENTIATION REGULATOR-LIKE | METRNL | 2.5 | 1 |
| Q01650 | SOLUTE CARRIER FAMILY 7 (CATIONIC AMINO ACID TRANSPORTER, Y+ SYSTEM), MEMBER 5 | SLC7A5 | 2.5 | 1.2 |
| Q8N7H1 | CHROMOSOME 12 OPEN READING FRAME 61 | C12ORF61 | 2.4 | -1.6 |
| Q96RQ9 | INTERLEUKIN 4 INDUCED 1 | IL4I1 | 2.4 | 1 |
| P18847 | ACTIVATING TRANSCRIPTION FACTOR 3 | ATF3 | 2.3 | 1.3 |
| Q9Y251 | HEPARANASE | HPSE | 2.3 | -1.2 |
| Q5VYS4 | HYPOTHETICAL PROTEIN FLJ14834 | C13ORF33 | 2.3 | 1.7 |
| Q9Y5U4 | INSULIN INDUCED GENE 2 | INSIG2 | 2.3 | -1.2 |
| Q13261 | INTERLEUKIN 15 RECEPTOR, ALPHA | IL15RA | 2.3 | 1.6 |
| Q9Y616 | INTERLEUKIN-1 RECEPTOR-ASSOCIATED KINASE 3 | IRAK3 | 2.3 | 1.1 |
| Q9Y5X9 | LIPASE, ENDOTHELIAL | LIPG | 2.3 | 2.2 |
| Q8NA29 | MAJOR FACILITATOR SUPERFAMILY DOMAIN CONTAINING 2 | MFSD2 | 2.3 | 1 |
| Q9H1K6 | MESODERM DEVELOPMENT CANDIDATE 1 | MESDC1 | 2.3 | 1.2 |
| Q99650 | ONCOSTATIN M RECEPTOR | OSMR | 2.3 | 1.2 |
| P15153 | RAS-RELATED C3 BOTULINUM TOXIN SUBSTRATE 2 (RHO FAMILY, SMALL GTP BINDING PROTEIN RAC2) | RAC2 | 2.3 | 1.2 |
| P48380 | REGULATORY FACTOR X, 3 (INFLUENCES HLA CLASS II EXPRESSION) | RFX3 | 2.3 | 2.5 |
| Q8IZD6 | SOLUTE CARRIER FAMILY 22 (ORGANIC CATION TRANSPORTER), MEMBER 15 | SLC22A15 | 2.3 | -1.1 |
| O60488 | ACYL-COA SYNTHETASE LONG-CHAIN FAMILY MEMBER 4 | ACSL4 | 2.2 | 2.4 |
| P16615 | ATPASE, CA++ TRANSPORTING, CARDIAC MUSCLE, SLOW TWITCH 2 | ATP2A2 | 2.2 | -1.2 |
| P12643 | BONE MORPHOGENETIC PROTEIN 2 | BMP2 | 2.2 | 1.5 |
| P78560 | CASP2 AND RIPK1 DOMAIN CONTAINING ADAPTOR WITH DEATH DOMAIN | CRADD | 2.2 | -1.4 |
| Q9NQR7 | CHROMOSOME 14 OPEN READING FRAME 162 | C14ORF162 | 2.2 | 1 |
| Q8N7C4 | CHROMOSOME 6 OPEN READING FRAME 128 | C6ORF128 | 2.2 | 2.5 |
| Q6ICB0 | DNA SEGMENT, CHR 15, WAYNE STATE UNIVERSITY 75, EXPRESSED | D15WSU75E | 2.2 | -1.2 |
| O75953 | DNAJ (HSP40) HOMOLOG, SUBFAMILY B, MEMBER 5 | DNAJB5 | 2.2 | 1 |
| Q96ET8 | FAMILY WITH SEQUENCE SIMILARITY 18, MEMBER B2 | FAM18B2 | 2.2 | -1.1 |
| Q99075 | HEPARIN-BINDING EGF-LIKE GROWTH FACTOR | HBEGF | 2.2 | 1 |
| Q13753 | LAMININ, GAMMA 2 | LAMC2 | 2.2 | 2.9 |
| P01138 | NERVE GROWTH FACTOR, BETA POLYPEPTIDE | NGFB | 2.2 | 1.3 |
| Q03405 | PLASMINOGEN ACTIVATOR, UROKINASE RECEPTOR | PLAUR | 2.2 | 1.4 |
| P02735 | SERUM AMYLOID A1 | SAA1 | 2.2 | 1.1 |
| O14543 | SUPPRESSOR OF CYTOKINE SIGNALING 3 | SOCS3 | 2.2 | 1 |
| Q9ULX9 | V-MAF MUSCULOAPONEUROTIC FIBROSARCOMA ONCOGENE HOMOLOG F (AVIAN) | MAFF | 2.2 | 1.4 |
| P32929 | CYSTATHIONASE (CYSTATHIONINE GAMMA-LYASE) | CTH | 2.1 | -1.4 |
| O00622 | CYSTEINE-RICH, ANGIOGENIC INDUCER, 61 | CYR61 | 2.1 | -1.1 |
| P05305 | ENDOTHELIN 1 | EDN1 | 2.1 | 1 |
| P30679 | GUANINE NUCLEOTIDE BINDING PROTEIN (G PROTEIN), ALPHA 15 (GQ CLASS) | GNA15 | 2.1 | 1.3 |
| O95394 | PHOSPHOGLUCOMUTASE 3 | PGM3 | 2.1 | 1 |
| P43004 | SOLUTE CARRIER FAMILY 1 (GLIAL HIGH AFFINITY GLUTAMATE TRANSPORTER), MEMBER 2 | SLC1A2 | 2.1 | 6 |
| P21980 | TRANSGLUTAMINASE 2 (C POLYPEPTIDE, PROTEIN-GLUTAMINE-GAMMA-GLUTAMYLTRANSFERASE) | TGM2 | 2.1 | 1 |
| Q9Y4C5 | CARBOHYDRATE (N-ACETYLGLUCOSAMINE-6-O) SULFOTRANSFERASE 2 | CHST2 | 2 | 1.4 |
| P35523 | CHLORIDE CHANNEL 1, SKELETAL MUSCLE (THOMSEN DISEASE, AUTOSOMAL DOMINANT) | CLCN1 | 2 | 1.4 |
| P38936 | CYCLIN-DEPENDENT KINASE INHIBITOR 1A (P21, CIP1) | CDKN1A | 2 | 2.1 |
| Q13217 | DNAJ (HSP40) HOMOLOG, SUBFAMILY C, MEMBER 3 | DNAJC3 | 2 | -1.1 |
| Q9H8M9 | HYPOTHETICAL PROTEIN FLJ13391 | TMEM166 | 2 | -2.5 |
| Q96JA1 | LEUCINE-RICH REPEATS AND IMMUNOGLOBULIN-LIKE DOMAINS 1 | LRIG1 | 2 | -2.5 |
| Q9NWQ8 | PHOSPHOPROTEIN ASSOCIATED WITH GLYCOSPHINGOLIPID MICRODOMAINS 1 | PAG1 | 2 | -5 |
| P01135 | TRANSFORMING GROWTH FACTOR, ALPHA | TGFA | 2 | 1.5 |
| O75915 | ADP-RIBOSYLATION-LIKE FACTOR 6 INTERACTING PROTEIN 5 | ARL6IP5 | -2 | 1.1 |
| Q8IX05 | CD302 ANTIGEN | CD302 | -2 | -1.6 |
| Q8N0X4 | CITRATE LYASE BETA LIKE | CLYBL | -2 | -2 |
| Q86T13 | C-TYPE LECTIN DOMAIN FAMILY 14, MEMBER A | CLEC14A | -2 | 10 |
| O00115 | DEOXYRIBONUCLEASE II, LYSOSOMAL | DNASE2 | -2 | 1 |
| P49184 | DEOXYRIBONUCLEASE I-LIKE 1 | DNASE1L1 | -2 | -1.1 |
| Q9BXJ0 | DKFZP586B0621 PROTEIN | C1QTNF5 | -2 | -2.5 |
| Q9UBX5 | FIBULIN 5 | FBLN5 | -2 | 11 |
| Q03013 | GLUTATHIONE S-TRANSFERASE M4 | GSTM4 | -2 | -3 |
| Q14393 | GROWTH ARREST-SPECIFIC 6 | GAS6 | -2 | 1.2 |
| Q8N4P3 | HD DOMAIN CONTAINING 3 | HDDC3 | -2 | -1.6 |
| Q96EF6 | HYPOTHETICAL PROTEIN FLJ11798 | FBXO17 | -2 | -5 |
| O60551 | N-MYRISTOYLTRANSFERASE 2 | NMT2 | -2 | -1.4 |
| P41247 | PATATIN-LIKE PHOSPHOLIPASE DOMAIN CONTAINING 4 | PNPLA4 | -2 | -2.5 |
| Q86VD9 | PHOSPHATIDYLINOSITOL GLYCAN, CLASS Z | PIGZ | -2 | -1.4 |
| Q96PX1 | RING FINGER PROTEIN 157 | RNF157 | -2 | 1 |
| Q8WTV0 | SCAVENGER RECEPTOR CLASS B, MEMBER 1 | SCARB1 | -2 | -1.1 |
| Q9BV40 | VESICLE-ASSOCIATED MEMBRANE PROTEIN 8 (ENDOBREVIN) | VAMP8 | -2 | -1.1 |
| Q96IU2 | ZINC FINGER, BED-TYPE CONTAINING 3 | ZBED3 | -2 | -2.5 |
| P30043 | BILIVERDIN REDUCTASE B (FLAVIN REDUCTASE (NADPH)) | BLVRB | -2.1 | -2 |
| P07858 | CATHEPSIN B | CTSB | -2.1 | -1.1 |
| P22570 | FERREDOXIN REDUCTASE | FDXR | -2.1 | -1.6 |
| Q8ND71 | GTPASE, IMAP FAMILY MEMBER 8 | GIMAP8 | -2.1 | -22 |
| Q4G0N7 | HYPOTHETICAL PROTEIN LOC619208 | LOC619208 | -2.1 | 1 |
| Q9BSF0 | HYPOTHETICAL PROTEIN MGC13057 | MGC13057 | -2.1 | -3.4 |
| P13284 | INTERFERON, GAMMA-INDUCIBLE PROTEIN 30 | IFI30 | -2.1 | 1 |
| Q8ND94 | SIMILAR TO HYPOTHETICAL PROTEIN | LOC221091 | -2.1 | 0.2 |
| Q9NS62 | THROMBOSPONDIN, TYPE I, DOMAIN CONTAINING 1 | THSD1 | -2.1 | -5 |
| Q96LW9 | ZINC FINGER PROTEIN 323 | ZNF323 | -2.1 | -1.2 |
| Q8WUY1 | CHROMOSOME 8 OPEN READING FRAME 55 | C8ORF55 | -2.2 | -10 |
| Q9H7Y0 | CHROMOSOME X OPEN READING FRAME 36 | CXORF36 | -2.2 | 1.2 |
| Q96J86 | CYSTEINE/TYROSINE-RICH 1 | CYYR1 | -2.2 | -2.5 |
| P53355 | DEATH-ASSOCIATED PROTEIN KINASE 1 | DAPK1 | -2.2 | 1 |
| P35555 | FIBRILLIN 1 (MARFAN SYNDROME) | FBN1 | -2.2 | -1.2 |
| P22352 | GLUTATHIONE PEROXIDASE 3 (PLASMA) | GPX3 | -2.2 | -1.4 |
| Q99538 | LEGUMAIN | LGMN | -2.2 | 1.2 |
| O14917 | PROTOCADHERIN 17 | PCDH17 | -2.2 | -1.6 |
| Q96EQ8 | RING FINGER PROTEIN 125 | RNF125 | -2.2 | -2.5 |
| Q9UNP4 | ST3 BETA-GALACTOSIDE ALPHA-2,3-SIALYLTRANSFERASE 5 | ST3GAL5 | -2.2 | -1.2 |
| P55008 | ALLOGRAFT INFLAMMATORY FACTOR 1 | AIF1 | -2.3 | 1 |
| Q6YHK3 | CD109 ANTIGEN (GOV PLATELET ALLOANTIGENS) | CD109 | -2.3 | 1.1 |
| Q96GN5 | CELL DIVISION CYCLE ASSOCIATED 7-LIKE | CDCA7L | -2.3 | -1.4 |
| Q96CH1 | G PROTEIN-COUPLED RECEPTOR 146 | GPR146 | -2.3 | -2.5 |
| P17900 | GM2 GANGLIOSIDE ACTIVATOR | GM2A | -2.3 | -1.4 |
| P30047 | GTP CYCLOHYDROLASE I FEEDBACK REGULATOR | GCHFR | -2.3 | -2 |
| Q658N2 | KIAA0523 PROTEIN | WSCD1 | -2.3 | -3.3 |
| Q9GZN7 | LEUCINE ZIPPER DOMAIN PROTEIN | ROGDI | -2.3 | -23 |
| Q9BX97 | PLASMALEMMA VESICLE ASSOCIATED PROTEIN | PLVAP | -2.3 | -1.1 |
| Q9UHI5 | SOLUTE CARRIER FAMILY 7 (CATIONIC AMINO ACID TRANSPORTER, Y+ SYSTEM), MEMBER 8 | SLC7A8 | -2.3 | -2 |
| Q5SZD1 | CHROMOSOME 6 OPEN READING FRAME 141 | C6ORF141 | -2.4 | -2 |
| Q9HA72 | FAMILY WITH SEQUENCE SIMILARITY 26, MEMBER B | FAM26B | -2.4 | -22 |
| Q13642 | FOUR AND A HALF LIM DOMAINS 1 | FHL1 | -2.4 | -1.6 |
| P46439 | GLUTATHIONE S-TRANSFERASE M5 | GSTM5 | -2.4 | -1.1 |
| O60262 | GUANINE NUCLEOTIDE BINDING PROTEIN (G PROTEIN), GAMMA 7 | GNG7 | -2.4 | -1.1 |
| Q9UBK5 | HEMATOPOIETIC CELL SIGNAL TRANSDUCER | HCST | -2.4 | -1.4 |
| Q14626 | INTERLEUKIN 11 RECEPTOR, ALPHA | IL11RA | -2.4 | -1.4 |
| Q9ULP0 | NDRG FAMILY MEMBER 4 | NDRG4 | -2.4 | 1 |
| Q68BL7 | OLFACTOMEDIN-LIKE 2A | OLFML2A | -2.4 | -3.3 |
| O75192 | PEROXISOMAL BIOGENESIS FACTOR 11A | PEX11A | -2.4 | -1.1 |
| O75365 | PROTEIN TYROSINE PHOSPHATASE TYPE IVA, MEMBER 3 | PTP4A3 | -2.4 | -1.4 |
| Q9BV35 | SOLUTE CARRIER FAMILY 25 (MITOCHONDRIAL CARRIER; PHOSPHATE CARRIER), MEMBER 23 | SLC25A23 | -2.4 | 1.4 |
| Q8IWU5 | SULFATASE 2 | SULF2 | -2.4 | -1.2 |
| Q9H3G5 | CARBOXYPEPTIDASE, VITELLOGENIC-LIKE | CPVL | -2.5 | -2.5 |
| Q9Y2V0 | CHROMOSOME 15 OPEN READING FRAME 41 | C15ORF41 | -2.5 | -3.3 |
| P27658 | COLLAGEN, TYPE VIII, ALPHA 1 | COL8A1 | -2.5 | -1.2 |
| Q9NRT5 | COLLAGEN, TYPE VIII, ALPHA 1 | COL8A1 | -2.5 | 1.3 |
| Q9ULC0 | ENDOMUCIN | EMCN | -2.5 | -3.3 |
| P23142 | FIBULIN 1 | FBLN1 | -2.5 | 1 |
| P17481 | HOMEOBOX B8 | HOXB8 | -2.5 | -1.2 |
| Q9NWW0 | HOST CELL FACTOR C1 REGULATOR 1 (XPO1 DEPENDENT) | HCFC1R1 | -2.5 | 1 |
| P21757 | MACROPHAGE SCAVENGER RECEPTOR 1 | MSR1 | -2.5 | -1.2 |
| Q9H8H3 | METHYLTRANSFERASE LIKE 7A | METTL7A | -2.5 | -1.2 |
| P48745 | NEPHROBLASTOMA OVEREXPRESSED GENE | NOV | -2.5 | 1.3 |
| O00584 | RIBONUCLEASE T2 | RNASET2 | -2.5 | -2 |
| Q8NFX7 | SYNTAXIN BINDING PROTEIN 6 (AMISYN) | STXBP6 | -2.5 | 0.2 |
| P02452 | COLLAGEN, TYPE I, ALPHA 1 | COL1A1 | -2.6 | 1.2 |
| O14645 | DYNEIN, AXONEMAL, LIGHT INTERMEDIATE POLYPEPTIDE 1 | DNALI1 | -2.6 | -1.6 |
| Q92743 | HTRA SERINE PEPTIDASE 1 | HTRA1 | -2.6 | -1.4 |
| O00534 | LOSS OF HETEROZYGOSITY, 11, CHROMOSOMAL REGION 2, GENE A | LOH11CR2A | -2.6 | -1.6 |
| P61626 | LYSOZYME (RENAL AMYLOIDOSIS) | LYZ | -2.6 | 1 |
| Q96JB6 | LYSYL OXIDASE-LIKE 4 | LOXL4 | -2.6 | 3 |
| Q5SRE7 | PHYTANOYL-COA DIOXYGENASE DOMAIN CONTAINING 1 | PHYHD1 | -2.6 | -5 |
| Q6UXH9 | REGENERATION ASSOCIATED MUSCLE PROTEASE | DKFZP586H2123 | -2.6 | 1 |
| P26447 | S100 CALCIUM BINDING PROTEIN A4 (CALCIUM PROTEIN, CALVASCULIN, METASTASIN, MURINE PLACENTAL HOMOLOG) | S100A4 | -2.6 | -1.6 |
| Q6PJ69 | TRIPARTITE MOTIF-CONTAINING 65 | TRIM65 | -2.6 | 1 |
| P48728 | AMINOMETHYLTRANSFERASE (GLYCINE CLEAVAGE SYSTEM PROTEIN T) | AMT | -2.7 | -1.1 |
| O75493 | CARBONIC ANHYDRASE XI | CA11 | -2.7 | -1.6 |
| P34913 | EPOXIDE HYDROLASE 2, CYTOPLASMIC | EPHX2 | -2.7 | -5 |
| P10912 | GROWTH HORMONE RECEPTOR | GHR | -2.7 | -1.4 |
| Q9BSA9 | HYPOTHETICAL PROTEIN MGC4618 | TMEM175 | -2.7 | 1.6 |
| Q06455 | RUNT-RELATED TRANSCRIPTION FACTOR 1; TRANSLOCATED TO, 1 (CYCLIN D-RELATED) | RUNX1T1 | -2.7 | 1.2 |
| Q7L0X0 | KIAA0644 GENE PRODUCT | KIAA0644 | -2.8 | -1.4 |
| Q9NR34 | MANNOSIDASE, ALPHA, CLASS 1C, MEMBER 1 | MAN1C1 | -2.8 | -1.2 |
| Q9HCL0 | PROTOCADHERIN 18 | PCDH18 | -2.8 | -1.2 |
| Q96R05 | RETINOL BINDING PROTEIN 7, CELLULAR | RBP7 | -2.8 | -1.2 |
| Q9GZM7 | TUBULOINTERSTITIAL NEPHRITIS ANTIGEN-LIKE 1 | TINAGL1 | -2.8 | -5 |
| Q9H665 | U2(RNU2) SMALL NUCLEAR RNA AUXILIARY FACTOR 1-LIKE 4 | TMEM149 | -2.8 | -23 |
| P29972 | AQUAPORIN 1 (COLTON BLOOD GROUP) | AQP1 | -2.9 | 0.2 |
| Q9GZN8 | CHROMOSOME 20 OPEN READING FRAME 27 | C20ORF27 | -2.9 | -1.6 |
| O95990 | FAMILY WITH SEQUENCE SIMILARITY 107, MEMBER A | FAM107A | -2.9 | 1.2 |
| Q5XXA6 | TRANSMEMBRANE PROTEIN 16A | TMEM16A | -2.9 | 1.1 |
| P16671 | CD36 ANTIGEN (COLLAGEN TYPE I RECEPTOR, THROMBOSPONDIN RECEPTOR) | CD36 | -3 | 1.1 |
| Q14314 | FIBRINOGEN-LIKE 2 | FGL2 | -3 | -1.6 |
| Q96RP7 | GALACTOSE-3-O-SULFOTRANSFERASE 4 | GAL3ST4 | -3 | -2 |
| Q6PII5 | HYDROXYACYLGLUTATHIONE HYDROLASE-LIKE | HAGHL | -3 | 1.1 |
| P38571 | LIPASE A, LYSOSOMAL ACID, CHOLESTEROL ESTERASE (WOLMAN DISEASE) | LIPA | -3 | 1 |
| Q6UX71 | PLEXIN DOMAIN CONTAINING 2 | PLXDC2 | -3 | -1.6 |
| P10586 | PROTEIN TYROSINE PHOSPHATASE, RECEPTOR TYPE, F | PTPRF | -3 | 1 |
| P20062 | TRANSCOBALAMIN II; MACROCYTIC ANEMIA | TCN2 | -3 | -3.3 |
| Q8NHE4 | ATPASE, H+ TRANSPORTING V0 SUBUNIT E2-LIKE (RAT) | ATP6V0E2 | -3.1 | -3.3 |
| Q9HCU0 | CD248 ANTIGEN, ENDOSIALIN | CD248 | -3.1 | 1.1 |
| P50440 | GLYCINE AMIDINOTRANSFERASE (L-ARGININE:GLYCINE AMIDINOTRANSFERASE) | GATM | -3.1 | -2.5 |
| Q9H1C3 | GLYCOSYLTRANSFERASE 8 DOMAIN CONTAINING 2 | GLT8D2 | -3.1 | 1 |
| Q7Z5S9 | HYPOTHETICAL PROTEIN FLJ11155 | TMEM144 | -3.1 | 1.2 |
| P53779 | MITOGEN-ACTIVATED PROTEIN KINASE 10 | MAPK10 | -3.1 | -2 |
| P01023 | ALPHA-2-MACROGLOBULIN | A2M | -3.2 | -1.2 |
| P29400 | COLLAGEN, TYPE IV, ALPHA 5 (ALPORT SYNDROME) | COL4A5 | -3.2 | 1 |
| P82987 | ADAMTS-LIKE 3 | ADAMTSL3 | -3.3 | 1 |
| Q96AM1 | MAS-RELATED GPR, MEMBER F | MRGPRF | -3.3 | 1.2 |
| Q15198 | PLATELET-DERIVED GROWTH FACTOR RECEPTOR-LIKE | PDGFRL | -3.3 | 1.3 |
| O75094 | SLIT HOMOLOG 3 (DROSOPHILA) | SLIT3 | -3.3 | -3.3 |
| Q8WV28 | B-CELL LINKER | BLNK | -3.4 | -1.2 |
| P04066 | FUCOSIDASE, ALPHA-L- 1, TISSUE | FUCA1 | -3.4 | -2 |
| P28067 | MAJOR HISTOCOMPATIBILITY COMPLEX, CLASS II, DM ALPHA | HLA-DMA | -3.4 | 1.2 |
| Q9Y275 | TUMOR NECROSIS FACTOR (LIGAND) SUPERFAMILY, MEMBER 13B | TNFSF13B | -3.4 | -1.6 |
| O14498 | IMMUNOGLOBULIN SUPERFAMILY CONTAINING LEUCINE-RICH REPEAT | ISLR | -3.5 | 1.4 |
| O14494 | PHOSPHATIDIC ACID PHOSPHATASE TYPE 2A | PPAP2A | -3.5 | -1.4 |
| O75129 | ASTROTACTIN 2 | ASTN2 | -3.6 | 1 |
| P81172 | HEPCIDIN ANTIMICROBIAL PEPTIDE | HAMP | -3.6 | 1.1 |
| P09529 | INHIBIN, BETA B (ACTIVIN AB BETA POLYPEPTIDE) | INHBB | -3.6 | -2.5 |
| Q96I82 | KAZAL-TYPE SERINE PEPTIDASE INHIBITOR DOMAIN 1 | KAZALD1 | -3.6 | 1 |
| P22459 | POTASSIUM VOLTAGE-GATED CHANNEL, SHAKER-RELATED SUBFAMILY, MEMBER 4 | KCNA4 | -3.6 | -1.1 |
| Q9H902 | RECEPTOR ACCESSORY PROTEIN 1 | REEP1 | -3.6 | -1.6 |
| P24557 | THROMBOXANE A SYNTHASE 1 (PLATELET, CYTOCHROME P450, FAMILY 5, SUBFAMILY A) | TBXAS1 | -3.6 | -1.1 |
| P08319 | ALCOHOL DEHYDROGENASE 4 (CLASS II), PI POLYPEPTIDE | ADH4 | -3.7 | -10 |
| P41732 | TETRASPANIN 7 | TSPAN7 | -3.7 | -5 |
| Q8TB45 | DEP DOMAIN CONTAINING 6 | DEPDC6 | -3.8 | -1.6 |
| P30154 | PROTEIN PHOSPHATASE 2 (FORMERLY 2A), REGULATORY SUBUNIT A (PR 65), BETA ISOFORM | PPP2R1B | -3.8 | 1.3 |
| O94907 | DICKKOPF HOMOLOG 1 (XENOPUS LAEVIS) | DKK1 | -3.9 | 1.6 |
| Q9NRN5 | OLFACTOMEDIN-LIKE 3 | OLFML3 | -3.9 | -2 |
| Q8N6Y2 | LEUCINE RICH REPEAT CONTAINING 17 | LRRC17 | -4 | 1 |
| Q9GZP0 | PLATELET DERIVED GROWTH FACTOR D | PDGFD | -4 | 1 |
| Q13591 | SEMA DOMAIN, SEVEN THROMBOSPONDIN REPEATS (TYPE 1 AND TYPE 1-LIKE), TRANSMEMBRANE DOMAIN (TM) AND SHORT CYTOPLASMIC DOMAIN, (SEMAPHORIN) 5A | SEMA5A | -4.2 | 1 |
| Q969F0 | FETAL AND ADULT TESTIS EXPRESSED 1 | FATE1 | -4.3 | -8.8 |
| Q96HF1 | SECRETED FRIZZLED-RELATED PROTEIN 2 | SFRP2 | -4.4 | 1.2 |
| Q8N3T1 | UDP-N-ACETYL-ALPHA-D-GALACTOSAMINE:POLYPEPTIDE N-ACETYLGALACTOSAMINYLTRANSFERASE-LIKE 2 | GALNTL2 | -4.4 | 1 |
| P42330 | ALDO-KETO REDUCTASE FAMILY 1, MEMBER C3 (3-ALPHA HYDROXYSTEROID DEHYDROGENASE, TYPE II) | AKR1C3 | -4.6 | 1 |
| Q12805 | EGF-CONTAINING FIBULIN-LIKE EXTRACELLULAR MATRIX PROTEIN 1 | EFEMP1 | -4.6 | -2 |
| P29320 | EPH RECEPTOR A3 | EPHA3 | -4.8 | 1.7 |
| Q16647 | PROSTAGLANDIN I2 (PROSTACYCLIN) SYNTHASE | PTGIS | -4.9 | -2 |
| P02745 | COMPLEMENT COMPONENT 1, Q SUBCOMPONENT, A CHAIN | C1QA | -5 | -2 |
| Q9HCS2 | HYPOTHETICAL PROTEIN SIMILAR TO RAT CYP4F1 | CYP4F12 | -5.1 | -1.6 |
| P22748 | CARBONIC ANHYDRASE IV | CA4 | -5.3 | 2 |
| O00453 | LEUKOCYTE-SPECIFIC TRANSCRIPT 1 | LST1 | -5.3 | -2.5 |
| O76076 | WNT1 INDUCIBLE SIGNALING PATHWAY PROTEIN 2 | WISP2 | -5.3 | 1 |
| Q9H239 | MATRIX METALLOPEPTIDASE 28 | MMP28 | -5.5 | -1.1 |
| P28068 | MAJOR HISTOCOMPATIBILITY COMPLEX, CLASS II, DM BETA | HLA-DMB | -5.6 | -1.2 |
| O75325 | LEUCINE RICH REPEAT NEURONAL 5 | LRRN2 | -6 | -1.1 |
| Q8NAJ2 | CHROMOSOME 9 OPEN READING FRAME 106 | C9ORF106 | -6.2 | PROBE NOT PRESENT |
| Q6UWY5 | OLFACTOMEDIN-LIKE 1 | OLFML1 | -6.4 | 1 |
| P56705 | WINGLESS-TYPE MMTV INTEGRATION SITE FAMILY, MEMBER 4 | WNT4 | -6.7 | 1.2 |
| Q8N6D5 | ANKYRIN REPEAT DOMAIN 29 | ANKRD29 | -7.3 | -1.2 |
| Q6ZTQ4 | HYPOTHETICAL PROTEIN FLJ23834 | FLJ23834 | -7.4 | -2.5 |
| Q8WXS4 | TRANSMEMBRANE PROTEIN 37 | TMEM37 | -7.5 | -1.6 |
| Q8IV16 | HIGH DENSITY LIPOPROTEIN-BINDING PROTEIN | LOC338328 | -7.6 | 1 |
| Q9Y6A2 | CYTOCHROME P450, FAMILY 46, SUBFAMILY A, POLYPEPTIDE 1 | CYP46A1 | -7.7 | 1.2 |
| Q8TAX0 | ODD-SKIPPED RELATED 1 (DROSOPHILA) | OSR1 | -7.9 | -1.1 |
| Q9NR80 | RHO GUANINE NUCLEOTIDE EXCHANGE FACTOR (GEF) 4 | ARHGEF4 | -8 | 1 |
| Q07654 | TREFOIL FACTOR 3 (INTESTINAL) | TFF3 | -8.2 | 1.9 |
| O95050 | THIOESTER S-METHYLTRANSFERASE-LIKE | INMT | -9.1 | 1 |
| O60245 | BH-PROTOCADHERIN (BRAIN-HEART) | PCDH7 | -9.2 | -1.1 |
| Q9UI42 | CARBOXYPEPTIDASE A4 | CPA4 | -9.8 | 1.6 |
| Q96A84 | EMI DOMAIN CONTAINING 1 | EMID1 | -10 | -1.1 |
| O00602 | FICOLIN (COLLAGEN/FIBRINOGEN DOMAIN CONTAINING) 1 | FCN1 | -10.2 | -1.4 |
| O95711 | LYMPHOCYTE ANTIGEN 86 | LY86 | -10.2 | -1.6 |
| Q8TAV5 | CHROMOSOME 11 OPEN READING FRAME 45 | C11ORF45 | -11.1 | -1.6 |
| Q8IVN8 | RPE-SPONDIN | RPESP | -11.7 | 3 |
| Q9BWQ8 | FAS APOPTOTIC INHIBITORY MOLECULE 2 | FAIM2 | -12 | -3 |
| P14207 | FOLATE RECEPTOR 2 (FETAL) | FOLR2 | -12.3 | -2.5 |
| O00585 | CHEMOKINE (C-C MOTIF) LIGAND 21 | CCL21 | -12.7 | 1 |
| Q13201 | MULTIMERIN 1 | MMRN1 | -13.2 | -3.3 |
| Q86WI1 | POLYCYSTIC KIDNEY AND HEPATIC DISEASE 1 (AUTOSOMAL RECESSIVE)-LIKE 1 | PKHD1L1 | -13.4 | 1 |
| P46091 | G PROTEIN-COUPLED RECEPTOR 1 | GPR1 | -14.8 | 1 |
| Q7Z7D3 | V-SET DOMAIN CONTAINING T CELL ACTIVATION INHIBITOR 1 | VTCN1 | -14.9 | -6.6 |
| Q4KMG0 | CDON HOMOLOG (MOUSE) | CDON | -18 | 1.4 |
| O14610 | GUANINE NUCLEOTIDE BINDING PROTEIN (G PROTEIN), GAMMA TRANSDUCING ACTIVITY POLYPEPTIDE 2 | GNGT2 | -20 | 1.3 |
| Q6UWQ7 | INSULIN GROWTH FACTOR-LIKE FAMILY MEMBER 2 | IGFL2 | -20 | 1.4 |
| Q9NZC2 | TRIGGERING RECEPTOR EXPRESSED ON MYELOID CELLS 2 | TREM2 | -25 | 1.7 |
| O15197 | EPH RECEPTOR B6 | EPHB6 | -27.8 | -1.2 |
| Q15493 | REGUCALCIN (SENESCENCE MARKER PROTEIN-30) | RGN | -46.4 | -2 |
| Q9UJQ1 | CHROMOSOME 20 OPEN READING FRAME 103 | C20ORF103 | -50 | -2 |
| Q96KN8 | HRAS-LIKE SUPPRESSOR FAMILY, MEMBER 5 | HRASLS5 | -50 | -1.4 |
| Q9NYK1 | TOLL-LIKE RECEPTOR 7 | TLR7 | -50 | -1.4 |
| O95395 | GLUCOSAMINYL (N-ACETYL) TRANSFERASE 3, MUCIN TYPE | GCNT3 | -56 | 1.8 |
| P35368 | ADRENERGIC, ALPHA-1B-, RECEPTOR | ADRA1B | -100 | -1.1 |

The adipose tissue (AT) predicted secretome, (n=7). Genes present in AT were significantly changed (p≤0.05, FC>2) while the corresponding genes in liver tissue (LT), (n=5) were not significantly affected (p>0.05).

Additional file 4, Table S3

**The significant liver tissue predicted secretome.**

| ACCESSION | NAME | SYMBOL | FC LT | FC AT |
| --- | --- | --- | --- | --- |
| Q07325 | CHEMOKINE (C-X-C MOTIF) LIGAND 9 | CXCL9 | 69.3 | 1.8 |
| P19876 | CHEMOKINE (C-X-C MOTIF) LIGAND 3 | CXCL3 | 20.6 | 3.6 |
| O95633 | FOLLISTATIN-LIKE 3 (SECRETED GLYCOPROTEIN) | FSTL3 | 15.1 | 1 |
| Q13113 | PDZK1 INTERACTING PROTEIN 1 | PDZK1IP1 | 12.9 | 1.9 |
| P16619 | CHEMOKINE (C-C MOTIF) LIGAND 3-LIKE 1 | CCL3L3 | 10.7 | probe is not present |
| P19957 | PEPTIDASE INHIBITOR 3, SKIN-DERIVED (SKALP) | PI3 | 9.9 | 5 |
| O60603 | TOLL-LIKE RECEPTOR 2 | TLR2 | 7.7 | 3.9 |
| Q96CG8 | COLLAGEN TRIPLE HELIX REPEAT CONTAINING 1 | CTHRC1 | 7.3 | 1.1 |
| P80188 | LIPOCALIN 2 (ONCOGENE 24P3) | LCN2 | 7.2 | 3.1 |
| Q8N2G4 | LY6/PLAUR DOMAIN CONTAINING 1 | LYPD1 | 6.7 | 1 |
| Q16678 | CYTOCHROME P450, FAMILY 1, SUBFAMILY B, POLYPEPTIDE 1 | CYP1B1 | 6.6 | -1.6 |
| Q9NRD8 | DUAL OXIDASE 2 | DUOX2 | 5.5 | -2 |
| Q9C0K1 | SOLUTE CARRIER FAMILY 39 (ZINC TRANSPORTER), MEMBER 8 | SLC39A8 | 5.5 | 1.3 |
| Q92730 | RHO FAMILY GTPASE 1 | RND1 | 4.9 | 1.7 |
| Q03518 | TRANSPORTER 1, ATP-BINDING CASSETTE, SUB-FAMILY B (MDR/TAP) | TAP1 | 4.5 | 1.6 |
| Q9UBS5 | GAMMA-AMINOBUTYRIC ACID (GABA) B RECEPTOR, 1 | GABBR1 | 4.4 | -1.1 |
| Q9BT56 | CHROMOSOME 12 OPEN READING FRAME 39 | C12ORF39 | 4.4 | -1.2 |
| Q6P9A2 | UDP-N-ACETYL-ALPHA-D-GALACTOSAMINE:POLYPEPTIDE N-ACETYLGALACTOSAMINYLTRANSFERASE-LIKE 4 | GALNTL4 | 4.1 | -1.4 |
| O43521 | BCL2-LIKE 11 (APOPTOSIS FACILITATOR) | BCL2L11 | 3.9 | 1.9 |
| Q8WWX9 | SELENOPROTEIN M | SELM | 3.9 | 1.6 |
| P78504 | JAGGED 1 (ALAGILLE SYNDROME) | JAG1 | 3.8 | -1.1 |
| Q6PJE2 | POM (POM121 HOMOLOG, RAT) AND ZP3 FUSION | POMZP3 | 3.8 | 1.2 |
| Q8IVJ1 | SOLUTE CARRIER FAMILY 41, MEMBER 1 | SLC41A1 | 3.7 | 1.9 |
| P08842 | STEROID SULFATASE (MICROSOMAL), ARYLSULFATASE C, ISOZYME S | STS | 3.7 | -1.1 |
| P48307 | TISSUE FACTOR PATHWAY INHIBITOR 2 | TFPI2 | 3.7 | 1.9 |
| Q96CG3 | TRAF-INTERACTING PROTEIN WITH A FORKHEAD-ASSOCIATED DOMAIN | TIFA | 3.7 | 1.4 |
| O94808 | GLUTAMINE-FRUCTOSE-6-PHOSPHATE TRANSAMINASE 2 | GFPT2 | 3.6 | 1.7 |
| P13164 | INTERFERON INDUCED TRANSMEMBRANE PROTEIN 1 (9-27) | IFITM1 | 3.5 | -1.1 |
| P28065 | PROTEASOME (PROSOME, MACROPAIN) SUBUNIT, BETA TYPE, 9 (LARGE MULTIFUNCTIONAL PEPTIDASE 2) | PSMB9 | 3.4 | 1.1 |
| P09603 | COLONY STIMULATING FACTOR 1 (MACROPHAGE) | CSF1 | 3.3 | -1.6 |
| P12544 | GRANZYME A (GRANZYME 1, CYTOTOXIC T-LYMPHOCYTE-ASSOCIATED SERINE ESTERASE 3) | GZMA | 3.3 | -1.4 |
| P25774 | CATHEPSIN S | CTSS | 3.2 | 1.4 |
| P01116 | V-HA-RAS HARVEY RAT SARCOMA VIRAL ONCOGENE HOMOLOG | KRAS | 3.1 | 1.6 |
| Q86UD5 | HYPOTHETICAL PROTEIN BC009732 | NHEDC2 | 3.1 | 1.6 |
| P15291 | UDP-GAL:BETAGLCNAC BETA 1,4- GALACTOSYLTRANSFERASE, POLYPEPTIDE 1 | B4GALT1 | 3 | 1.6 |
| P24001 | INTERLEUKIN 32 | IL32 | 3 | 1.6 |
| P49788 | RETINOIC ACID RECEPTOR RESPONDER (TAZAROTENE INDUCED) 1 | RARRES1 | 2.9 | -2 |
| O43570 | CARBONIC ANHYDRASE XII | CA12 | 2.9 | 2.8 |
| P30305 | CELL DIVISION CYCLE 25B | CDC25B | 2.9 | 1 |
| P25942 | CD40 ANTIGEN (TNF RECEPTOR SUPERFAMILY MEMBER 5) | CD40 | 2.8 | 1.8 |
| P80098 | CHEMOKINE (C-C MOTIF) LIGAND 7 | CCL7 | 2.8 | 1.8 |
| Q969K7 | TRANSMEMBRANE PROTEIN 54 | TMEM54 | 2.8 | -1.1 |
| Q13641 | TROPHOBLAST GLYCOPROTEIN | TPBG | 2.8 | 1.2 |
| Q92519 | TRIBBLES HOMOLOG 2 (DROSOPHILA) | TRIB2 | 2.8 | -2 |
| Q8N961 | ANKYRIN REPEAT AND BTB (POZ) DOMAIN CONTAINING 2 | ABTB2 | 2.7 | 1.9 |
| O60443 | DEAFNESS, AUTOSOMAL DOMINANT 5 | DFNA5 | 2.7 | -1.1 |
| Q9Y2P8 | RNA TERMINAL PHOSPHATE CYCLASE-LIKE 1 | RCL1 | 2.7 | 1.3 |
| P31431 | SYNDECAN 4 (AMPHIGLYCAN, RYUDOCAN) | SDC4 | 2.7 | 1.9 |
| P03973 | SECRETORY LEUKOCYTE PEPTIDASE INHIBITOR | SLPI | 2.7 | -1.4 |
| P09237 | MATRIX METALLOPEPTIDASE 7 (MATRILYSIN, UTERINE) | MMP7 | 2.6 | -1.6 |
| P23497 | SP100 NUCLEAR ANTIGEN | SP100 | 2.6 | 1.7 |
| Q14201 | BTG FAMILY, MEMBER 3 | BTG3 | 2.5 | 1.7 |
| O00478 | BUTYROPHILIN, SUBFAMILY 3, MEMBER A3 | BTN3A3 | 2.5 | 1.3 |
| Q8N682 | HYPOTHETICAL PROTEIN FLJ11259 | DRAM | 2.5 | 1.6 |
| Q5VY09 | IMMEDIATE EARLY RESPONSE 5 | IER5 | 2.5 | 1.6 |
| Q92537 | KIAA0247 | KIAA0247 | 2.5 | 1.8 |
| Q96FA3 | PELLINO HOMOLOG 1 (DROSOPHILA) | PELI1 | 2.5 | 1.4 |
| Q96A46 | SOLUTE CARRIER FAMILY 25, MEMBER 28 | SLC25A28 | 2.5 | 1.4 |
| Q9Y3F1 | TRANSPORTER 2, ATP-BINDING CASSETTE, SUB-FAMILY B (MDR/TAP) | TAP2 | 2.5 | 1.5 |
| Q9NZC4 | ETS HOMOLOGOUS FACTOR | EHF | 2.4 | 2.7 |
| P11309 | PIM-1 ONCOGENE | PIM1 | 2.4 | 1 |
| O15533 | TAP BINDING PROTEIN (TAPASIN) | TAPBP | 2.4 | 1.5 |
| Q8NFZ5 | TNFAIP3 INTERACTING PROTEIN 2 | TNIP2 | 2.4 | 1.3 |
| P43897 | TS TRANSLATION ELONGATION FACTOR, MITOCHONDRIAL | TSFM | 2.4 | 1.5 |
| Q6RW13 | ANGIOTENSIN II RECEPTOR-ASSOCIATED PROTEIN | AGTRAP | 2.3 | 1.3 |
| Q08722 | CD47 ANTIGEN (RH-RELATED ANTIGEN, INTEGRIN-ASSOCIATED SIGNAL TRANSDUCER) | CD47 | 2.3 | -1.4 |
| Q07065 | CYTOSKELETON-ASSOCIATED PROTEIN 4 | CKAP4 | 2.3 | 1.2 |
| O75881 | CYTOCHROME P450, FAMILY 7, SUBFAMILY B, POLYPEPTIDE 1 | CYP7B1 | 2.3 | 1.2 |
| Q9UK22 | F-BOX PROTEIN 2 | FBXO2 | 2.3 | -1.2 |
| P78536 | ADAM METALLOPEPTIDASE DOMAIN 17 (TUMOR NECROSIS FACTOR, ALPHA, CONVERTING ENZYME) | ADAM17 | 2.2 | 1.3 |
| O75976 | CARBOXYPEPTIDASE D | CPD | 2.2 | -25 |
| Q9NQC7 | CYLINDROMATOSIS (TURBAN TUMOR SYNDROME) | CYLD | 2.2 | 1.2 |
| P30511 | MAJOR HISTOCOMPATIBILITY COMPLEX, CLASS I, F | HLA-F | 2.1 | 1.7 |
| Q7Z7G0 | ABI GENE FAMILY, MEMBER 3 (NESH) BINDING PROTEIN | ABI3BP | 2.1 | 1.6 |
| P40306 | PROTEASOME (PROSOME, MACROPAIN) SUBUNIT, BETA TYPE, 10 | PSMB10 | 2.1 | 1.1 |
| P49281 | SOLUTE CARRIER FAMILY 11 (PROTON-COUPLED DIVALENT METAL ION TRANSPORTERS), MEMBER 2 | SLC11A2 | 2.1 | 1.6 |
| Q9UJT1 | TUBULIN, DELTA 1 | TUBD1 | 2.1 | 1 |
| P55957 | BH3 INTERACTING DOMAIN DEATH AGONIST | BID | 2 | 1.2 |
| O14569 | CYTOCHROME B-561 DOMAIN CONTAINING 2 | CYB561D2 | 2 | 1.1 |
| Q53QV2 | HYPOTHETICAL PROTEIN DKFZP566J091 | LBH | 2 | -1.4 |
| P31358 | CD52 ANTIGEN (CAMPATH-1 ANTIGEN) | CD52 | -2 | -1.1 |
| Q8N4T8 | CARBONIC REDUCTASE 4 | CBR4 | -2 | -1.4 |
| O95471 | CLAUDIN 7 | CLDN7 | -2 | 1.4 |
| Q14520 | HYALURONAN BINDING PROTEIN 2 | HABP2 | -2 | 1 |
| Q9H6X4 | HYPOTHETICAL PROTEIN FLJ21749 | TMEM134 | -2 | 1 |
| Q9BYT8 | NEUROLYSIN (METALLOPEPTIDASE M3 FAMILY) | NLN | -2.1 | -1.6 |
| Q8N2U9 | PQ LOOP REPEAT CONTAINING 1 | PQLC1 | -2.1 | -1.4 |
| P37173 | TRANSFORMING GROWTH FACTOR, BETA RECEPTOR II (70/80KDA) | TGFBR2 | -2.2 | -1.6 |
| Q8N661 | TRANSMEMBRANE PROTEIN 86B | TMEM86B | -2.2 | 1 |
| Q9Y5U8 | BRAIN PROTEIN 44-LIKE | BRP44L | -2.2 | -1.6 |
| Q96EK6 | GLUCOSAMINE-PHOSPHATE N-ACETYLTRANSFERASE 1 | GNPNAT1 | -2.2 | 1.2 |
| P19823 | INTER-ALPHA (GLOBULIN) INHIBITOR H2 | ITIH2 | -2.2 | -1.2 |
| Q9H477 | RIBOKINASE | RBKS | -2.2 | 1.1 |
| P16035 | TIMP METALLOPEPTIDASE INHIBITOR 2 | TIMP2 | -2.2 | 1.1 |
| Q13885 | TUBULIN, BETA 2A | TUBB2A | -2.2 | -1.2 |
| Q9BVA1 | TUBULIN, BETA 2B | TUBB2B | -2.2 | 1.3 |
| P23526 | S-ADENOSYLHOMOCYSTEINE HYDROLASE | AHCY | -2.3 | -1.2 |
| Q86SX6 | GLUTAREDOXIN 5 HOMOLOG (S. CEREVISIAE) | GLRX5 | -2.3 | -1.2 |
| P02765 | ALPHA-2-HS-GLYCOPROTEIN | AHSG | -2.4 | -1.1 |
| Q08257 | CRYSTALLIN, ZETA (QUINONE REDUCTASE) | CRYZ | -2.4 | -1.4 |
| P23434 | GLYCINE CLEAVAGE SYSTEM PROTEIN H (AMINOMETHYL CARRIER) | GCSH | -2.4 | -1.1 |
| Q6NUM9 | ALL-TRANS-13,14-DIHYDRORETINOL SATURASE | RETSAT | -2.4 | -1.6 |
| P18827 | SYNDECAN 1 | SDC1 | -2.4 | 1 |
| P51795 | CHLORIDE CHANNEL 5 (NEPHROLITHIASIS 2, X-LINKED, DENT DISEASE) | CLCN5 | -2.5 | 1.2 |
| Q92496 | COMPLEMENT FACTOR H-RELATED 4 | CFHR4 | -2.5 | -1.1 |
| O60894 | RECEPTOR (CALCITONIN) ACTIVITY MODIFYING PROTEIN 1 | RAMP1 | -2.6 | 1.1 |
| P51690 | ARYLSULFATASE E (CHONDRODYSPLASIA PUNCTATA 1) | ARSE | -2.6 | 1.2 |
| O00748 | CARBOXYLESTERASE 2 (INTESTINE, LIVER) | CES2 | -2.6 | -1.1 |
| O75600 | GLYCINE C-ACETYLTRANSFERASE (2-AMINO-3-KETOBUTYRATE COENZYME A LIGASE) | GCAT | -2.6 | -1.1 |
| P13598 | INTERCELLULAR ADHESION MOLECULE 2 | ICAM2 | -2.6 | -1.4 |
| Q9NVS9 | PYRIDOXINE 5'-PHOSPHATE OXIDASE | PNPO | -2.6 | -1.4 |
| Q92667 | A KINASE (PRKA) ANCHOR PROTEIN 1 | AKAP1 | -2.7 | 1.2 |
| Q96DC8 | ENOYL COENZYME A HYDRATASE DOMAIN CONTAINING 3 | ECHDC3 | -2.7 | -1.2 |
| Q8IYQ7 | THREONINE SYNTHASE-LIKE 1 (BACTERIAL) | THNSL1 | -2.7 | -1.4 |
| P35625 | TIMP METALLOPEPTIDASE INHIBITOR 3 (SORSBY FUNDUS DYSTROPHY, PSEUDOINFLAMMATORY) | TIMP3 | -2.7 | -1.1 |
| Q6NVY1 | 3-HYDROXYISOBUTYRYL-COENZYME A HYDROLASE | HIBCH | -2.8 | -1.4 |
| Q9NVV5 | ANDROGEN-INDUCED 1 | AIG1 | -3 | -1.6 |
| Q92947 | GLUTARYL-COENZYME A DEHYDROGENASE | GCDH | -3 | -1.2 |
| P30086 | PHOSPHATIDYLETHANOLAMINE BINDING PROTEIN 1 | PEBP1 | -3.1 | -1.4 |
| O15539 | REGULATOR OF G-PROTEIN SIGNALLING 5 | RGS5 | -3.1 | -3.3 |
| Q96JT2 | SOLUTE CARRIER FAMILY 45, MEMBER 3 | SLC45A3 | -3.1 | -1.1 |
| P22607 | FIBROBLAST GROWTH FACTOR RECEPTOR 3 (ACHONDROPLASIA, THANATOPHORIC DWARFISM) | FGFR3 | -3.2 | 1.1 |
| P04196 | HISTIDINE-RICH GLYCOPROTEIN | HRG | -3.2 | 1.1 |
| P16118 | 6-PHOSPHOFRUCTO-2-KINASE/FRUCTOSE-2,6-BIPHOSPHATASE 1 | PFKFB1 | -3.2 | 1 |
| P22760 | ARYLACETAMIDE DEACETYLASE (ESTERASE) | AADAC | -3.4 | -1.4 |
| Q6ZVE7 | GOLGI TRANSPORT 1 HOMOLOG A (S. CEREVISIAE) | GOLT1A | -3.4 | 1 |
| Q15466 | NUCLEAR RECEPTOR SUBFAMILY 0, GROUP B, MEMBER 2 | NR0B2 | -3.5 | 1 |
| Q96B21 | TRANSMEMBRANE PROTEIN 45B | TMEM45B | -3.5 | 1 |
| Q13609 | DEOXYRIBONUCLEASE I-LIKE 3 | DNASE1L3 | -3.5 | -1.6 |
| Q8TEB7 | RING FINGER PROTEIN 128 | RNF128 | -3.5 | 1 |
| P12830 | CADHERIN 1, TYPE 1, E-CADHERIN (EPITHELIAL) | CDH1 | -3.6 | 1.3 |
| P18065 | INSULIN-LIKE GROWTH FACTOR BINDING PROTEIN 2, 36KDA | IGFBP2 | -3.6 | 2.1 |
| Q96I34 | PROTEIN PHOSPHATASE 1, REGULATORY (INHIBITOR) SUBUNIT 16A | PPP1R16A | -3.6 | -1.1 |
| Q9UNA0 | ADAM METALLOPEPTIDASE WITH THROMBOSPONDIN TYPE 1 MOTIF, 5 (AGGRECANASE-2) | ADAMTS5 | -3.7 | -2.5 |
| Q9Y5C1 | ANGIOPOIETIN-LIKE 3 | ANGPTL3 | -3.7 | 1.2 |
| P07306 | ASIALOGLYCOPROTEIN RECEPTOR 1 | ASGR1 | -3.8 | -1.4 |
| Q9Y2P5 | SOLUTE CARRIER FAMILY 27 (FATTY ACID TRANSPORTER), MEMBER 5 | SLC27A5 | -3.8 | -2.5 |
| Q9H0T7 | RAB17, MEMBER RAS ONCOGENE FAMILY | RAB17 | -4 | 1 |
| Q9BSE5 | AGMATINE UREOHYDROLASE (AGMATINASE) | AGMAT | -4.2 | 1.5 |
| P01042 | KININOGEN 1 | KNG1 | -4.2 | 1.2 |
| Q6Q0C1 | CHROMOSOME 14 OPEN READING FRAME 68 | C14ORF68 | -4.3 | 1.2 |
| Q9UHD4 | CELL DEATH-INDUCING DFFA-LIKE EFFECTOR B | CIDEB | -4.3 | -1.2 |
| Q16534 | HEPATIC LEUKEMIA FACTOR | HLF | -4.3 | 1 |
| O75452 | RETINOL DEHYDROGENASE 16 (ALL-TRANS AND 13-CIS) | RDH16 | -4.3 | 1 |
| Q9UHE5 | N-ACETYLTRANSFERASE 8 (CAMELLO LIKE) | NAT8 | -4.4 | -2.53 |
| P08034 | GAP JUNCTION PROTEIN, BETA 1, 32KDA (CONNEXIN 32, CHARCOT-MARIE-TOOTH NEUROPATHY, X-LINKED) | GJB1 | -4.7 | -25 |
| O95154 | ALDO-KETO REDUCTASE FAMILY 7, MEMBER A3 (AFLATOXIN ALDEHYDE REDUCTASE) | AKR7A3 | -5.3 | -1.2 |
| O94911 | ATP-BINDING CASSETTE, SUB-FAMILY A (ABC1), MEMBER 8 | ABCA8 | -5.5 | -23 |
| P11226 | MANNOSE-BINDING LECTIN (PROTEIN C) 2, SOLUBLE (OPSONIC DEFECT) | MBL2 | -5.6 | 1 |
| Q9H2X3 | C-TYPE LECTIN DOMAIN FAMILY 4, MEMBER M | CLEC4M | -5.9 | probe not present |
| P11712 | CYTOCHROME P450, FAMILY 2, SUBFAMILY C, POLYPEPTIDE 9 | CYP2C9 | -6.0 | 1 |
| Q9UF12 | PROLINE DEHYDROGENASE (OXIDASE) 2 | PRODH2 | -6.1 | 3.2 |
| Q6UXB4 | C-TYPE LECTIN SUPERFAMILY 4, MEMBER G | CLEC4G | -6.3 | probe not present |
| Q15063 | PERIOSTIN, OSTEOBLAST SPECIFIC FACTOR | POSTN | -6.4 | -2.5 |
| P05546 | SERPIN PEPTIDASE INHIBITOR, CLADE D (HEPARIN COFACTOR), MEMBER 1 | SERPIND1 | -6.4 | 2.5 |
| O14756 | HYDROXYSTEROID (17-BETA) DEHYDROGENASE 6 | HSD17B6 | -6.7 | -10 |
| Q14956 | GLYCOPROTEIN (TRANSMEMBRANE) NMB | GPNMB | -6.9 | -2.5 |
| P05160 | COAGULATION FACTOR XIII, B POLYPEPTIDE | F13B | -7.0 | -1.1 |
| P22897 | MANNOSE RECEPTOR, C TYPE 1 | MRC1 | -7.2 | 1 |
| Q5VSK2 | MANNOSE RECEPTOR, C TYPE 1-LIKE 1 | MRC1L1 | -7.2 | 1 |
| P54855 | UDP GLUCURONOSYLTRANSFERASE 2 FAMILY, POLYPEPTIDE B15 | UGT2B15 | -7.8 | 1.1 |
| Q15485 | FICOLIN (COLLAGEN/FIBRINOGEN DOMAIN CONTAINING LECTIN) 2 (HUCOLIN) | FCN2 | -10.1 | -1.1 |
| Q8WWZ8 | ONCOPROTEIN INDUCED TRANSCRIPT 3 | OIT3 | -10.3 | -1.4 |
| Q16627 | CHEMOKINE (C-C MOTIF) LIGAND 14 | CCL14 | -10.6 | probe not present |
| Q02338 | 3-HYDROXYBUTYRATE DEHYDROGENASE, TYPE 1 | BDH1 | -11.7 | -1.1 |
| Q969E1 | LIVER-EXPRESSED ANTIMICROBIAL PEPTIDE 2 | LEAP2 | -11.7 | 1.4 |
| Q9Y694 | SOLUTE CARRIER FAMILY 22 (ORGANIC ANION TRANSPORTER), MEMBER 7 | SLC22A7 | -11.9 | 1.3 |
| P08684 | CYTOCHROME P450, SUBFAMILY IIIA (NIPHEDIPINE OXIDASE), POLYPEPTIDE 3 | CYP3A4 | -12.1 | 1 |
| Q9H227 | GLUCOSIDASE, BETA, ACID 3 (CYTOSOLIC) | GBA3 | -12.1 | -1.2 |
| P48230 | TRANSMEMBRANE 4 L SIX FAMILY MEMBER 4 | TM4SF4 | -12.7 | -1.2 |
| Q9UJ72 | ANNEXIN A10 | ANXA10 | -16.0 | 1 |
| P31327 | CARBAMOYL-PHOSPHATE SYNTHETASE 1, MITOCHONDRIAL | CPS1 | -17.9 | -1.4 |
| P02768 | ALBUMIN | ALB | -20.4 | -1.1 |
| P11168 | SOLUTE CARRIER FAMILY 2 (FACILITATED GLUCOSE TRANSPORTER), MEMBER 2 | SLC2A2 | -29.9 | -1.1 |

The liver tissue (LT) predicted secretome, n=5. Genes present in LT were significantly changed ( p≤0.05, FC>2) while the corresponding genes in adipose tissue (AT), (n=7) were not significantly affected (p>0.05).
